# Molecular Phylogeny of the Small Ermine Moth Genus *Yponomeuta* (Lepidoptera, Yponomeutidae) in the Palaearctic

**DOI:** 10.1371/journal.pone.0009933

**Published:** 2010-03-29

**Authors:** Hubert Turner, Niek Lieshout, Wil E. Van Ginkel, Steph B. J. Menken

**Affiliations:** 1 Evolutionary Biology, Institute for Biodiversity and Ecosystem Dynamics, University of Amsterdam, Amsterdam, The Netherlands; 2 Netherlands Centre for Biodiversity Naturalis (section Nationaal Herbarium Nederland), Leiden, The Netherlands; McGill University, Canada

## Abstract

**Background:**

The small ermine moth genus *Yponomeuta* (Lepidoptera, Yponomeutidae) contains 76 species that are specialist feeders on hosts from Celastraceae, Rosaceae, Salicaceae, and several other plant families. The genus is a model for studies in the evolution of phytophagous insects and their host-plant associations. Here, we reconstruct the phylogeny to provide a solid framework for these studies, and to obtain insight into the history of host-plant use and the biogeography of the genus.

**Methodology/Principal Findings:**

DNA sequences from an internal transcribed spacer region (ITS-1) and from the 16S rDNA (16S) and cytochrome oxidase (COII) mitochondrial genes were collected from 20–23 (depending on gene) species and two outgroup taxa to reconstruct the phylogeny of the Palaearctic members of this genus. Sequences were analysed using three different phylogenetic methods (parsimony, likelihood, and Bayesian inference).

**Conclusions/Significance:**

Roughly the same patterns are retrieved irrespective of the method used, and they are similar among the three genes. Monophyly is well supported for a clade consisting of the Japanese (but not the Dutch) population of *Yponomeuta sedellus* and *Y. yanagawanus*, a *Y. kanaiellus–polystictus* clade, and a Rosaceae-feeding, western Palaearctic clade (*Y. cagnagellus–irrorellus* clade). Within these clades, relationships are less well supported, and the patterns between the different gene trees are not so similar. The position of the remaining taxa is also variable among the gene trees and rather weakly supported. The phylogenetic information was used to elucidate patterns of biogeography and resource use. In the Palaearctic, the genus most likely originated in the Far East, feeding on Celastraceae, dispersing to the West concomitant with a shift to Rosaceae and further to Salicaceae. The association of *Y*. *cagnagellus* with *Euonymus europaeus* (Celastraceae), however, is a reversal. The only oligophagous species, *Y*. *padellus*, belongs to the derived western Palaearctic clade, evidence that specialisation is reversible.

## Introduction

The majority of terrestrial species interactions concerns those of insects and plants [1]. Most phytophagous insects are diet specialists, i.e., they feed on one or a few plant species that are closely related (monophagous, host plants within one plant genus, or oligophagous, host plants within one family) [2]. The question of why organisms specialise in their resource use has been the subject of many evolutionary ecological studies (e.g., [3] and references therein). Furthermore, related insects often feed on related plants (so-called phylogenetic conservatism [4], [5]), yet patterns of co-evolution between phytophages and plants are very rare (exceptions are yucca moths of the family Prodoxidae and *Tetraopes* beetles [6], [7]). Instead, insect herbivores are supposed to have evolved against a background of pre-existing plant diversity through host shifts and the subsequent evolution of host races (so-called sequential evolution) [8]–[10]. Host races are populations of a species that are partly reproductively isolated from each other, due to adaptation to different food plants [11].

The small ermine moth genus *Yponomeuta* (Lepidoptera, Yponomeutidae) is widespread across the Palaearctic, from Japan in the East to western Europe and the Canary Islands in the West; it is also present in Africa, Southeast Asia, Australia, and New Zealand, while *Yponomeuta multipunctellus* occurs in North America [12], [13]. Females lay their eggs on twigs of a variety of host plants, mainly from the families Celastraceae and Rosaceae, on which the specialist larvae feed. While the majority is solitary, some species are gregarious, the larvae building large, loose nests from silk produced in glands (e.g., *Y. cagnagellus, Y. padellus*, and *Y. malinellus* [14]). The genus *Yponomeuta* has been a model taxon for multidisciplinary investigations into the evolution of insect–plant associations [9], [15]–[19]. Species are often pests on ornamentals and one is a threat to commercial apples (*Y*. *malinellus* [20]). The genus has a supposed ancestral host-plant association with the spindle tree family Celastraceae [16], [21]. Most present-day species maintain this association, but a number of mostly Central and West European taxa feed on Rosaceae and Salicaceae.

A well-supported phylogeny is indispensable for establishing taxon relationships and patterns of character evolution, resource use, and geographic distribution. Unfortunately, the only *Yponomeuta* ‘trees’ available till now are phenograms based on morphological traits and constructed for a mainly West European subset of the species [22], and the tree presented by Sperling et al. [23] for four species of *Yponomeuta*. Another phenogram, based on allozymes of a similar subset of species, is also available [24]. In this study, the number of taxa is increased by adding a number of East Asian taxa of which we could obtain DNA samples. We sequenced two mitochondrial genes (16S and COII) and one nuclear sequence (ITS-1), and reconstructed gene trees using different analytical approaches (maximum parsimony, maximum likelihood, and Bayesian inference). Mitochondrial genes and the ITS-1 region appear to be very useful markers for the resolution of phylogenetic relations at the within-genus level [e.g., 25].

As we were mostly interested in reconstructing the phylogenetic tree of species, we also analysed the different data sets in a total-evidence approach [26]. The results of our analyses of the three (partial) sequences using the different approaches outlined above are largely congruent, enabling us to derive general conclusions regarding the phylogeny, host switches, and biogeography of *Yponomeuta*.

## Materials and Methods

### Specimens

The species used in this study and their sampling localities are given in Table 1. Specimens were collected as L4 or L5 instars from their respective food plants, reared in the laboratory, and eclosed adults were frozen and stored at −70°C until used for DNA extraction. The European species were collected during 1994–1997, the Japanese species, including the outgroup *Xyrosaris lichneuta*, in 1994, the American species (*Yponomeuta multipunctellus*) was sent to us in 1995 by J.F. Landry (Agriculture Canada, Ottawa, Canada), and the outgroup *Euhyponomeutoides trachydeltus* was sent to us in 1998 by S. Moriuti (Entomological Laboratory, University of Osaka Prefecture, Japan). *Xyrosaris lichneuta* and *E. trachydeltus* belong to genera within the family Yponomeutidae, subfamily Yponomeutinae, and are the closest relatives of *Yponomeuta* that were available. Voucher specimens have been deposited in the collections of the Zoological Museum of the University of Amsterdam.

**Table 1:** Species included in the analyses and Genbank accession numbers for the various sequences.

Species	Origin	Food plant	Voucher	Genbank accession number		
				16S	COII	ITS-1
*Euhyponomeutoides trachydeltus* (Meyrick, 1931)	Nikko, Japan	*Euonymus fortunei*	ZMA.INS.LEPI.1	**AY543628**	**AY551030**	**AY551071**
*Xyrosaris lichneuta* Meyrick, 1918	Towada ko, Japan	*Euonymus alatus*	ZMA.INS.LEPI.2	**AY543619**	**AY551022**	**AY551062**
*Yponomeuta cagnagellus* (Hübner, 1813) 1	Hungary	*Euonymus europaeus*	ZMA.INS.LEPI.3			**AY551032**
*Yponomeuta cagnagellus* 2	Czech Republic	*Euonymus europaeus*	ZMA.INS.LEPI.4			**AY551033**
*Yponomeuta cagnagellus* 3	Wädenswil, Switzerland	*Euonymus europaeus*	ZMA.INS.LEPI.5			**AY551034**
*Yponomeuta cagnagellus* 4	Wädenswil, Switzerland	*Euonymus europaeus*	ZMA.INS.LEPI.6	**AY543605**	**AY551008**	**AY551048**
*Yponomeuta eurinellus* Zagulajev, 1969	Oze, Japan	*Euonymus macropterus*	ZMA.INS.LEPI.7	**AY543618**	**AY551021**	**AY551061**
*Yponomeuta evonymellus* (Linnaeus, 1758)	Kootwijk, Netherlands	*Prunus padus*	ZMA.INS.LEPI.8	**AY543612**	**AY551015**	
*Yponomeuta gigas* Rebel, 1892	La Palma, Spain	*Salix canariensis*	ZMA.INS.LEPI.9		**AY551014**	**AY551054**
*Yponomeuta griseatus* Moriuti, 1977	Senjogakara, Japan	*Euonymus sieboldianus*	ZMA.INS.LEPI.10	**AY543623**		**AY551066**
*Yponomeuta irrorellus* (Hübner, 1796)	Mook, Netherlands	*Euonymus europaeus*	ZMA.INS.LEPI.11	**AY543607**	**AY551010**	**AY551050**
*Yponomeuta kanaiellus* Matsumura, 1931	Oirase-gawa, Japan	*Euonymus alatus*	ZMA.INS.LEPI.12			**AY551067**
*Yponomeuta mahalebellus* Guenée, 1845	Aude, France	*Prunus mahaleb*	ZMA.INS.LEPI.13	**AY543609**	**AY551012**	**AY551052**
*Yponomeuta malinellus* Zeller, 1838	Wageningen, Netherlands	*Malus* spec.	ZMA.INS.LEPI.14	**AY543608**	**AY551011**	**AY551051**
*Yponomeuta meguronis* Matsumura, 1931	Nikko, Japan	*Euonymus fortunei*	ZMA.INS.LEPI.15	**AY543613**	**AY551016**	**AY551056**
*Yponomeuta menkeni* Gershenson & Ulenberg, 1998	Oirase-gawa, Japan	*Euonymus alatus*	ZMA.INS.LEPI.16	**AY543627**	**AY551029**	**AY551070**
*Yponomeuta multipunctellus* Clemens, 1860	Class County, MI, USA	??	ZMA.INS.LEPI.17		**AY551031**	**AY551072**
*Yponomeuta padellus* (Linnaeus, 1758) 1	Malden, Netherlands	*Amelanchier* spec.	ZMA.INS.LEPI.18	**AY543592**		**AY551035**
*Yponomeuta padellus* 2	Malden, Netherlands	*Crataegus* spec.	ZMA.INS.LEPI.19			**AY551036**
*Yponomeuta padellus* 3	Malden, Netherlands	*Prunus spinosa*	ZMA.INS.LEPI.20			**AY551037**
*Yponomeuta padellus* 4	Malden, Netherlands	*Sorbus* spec.	ZMA.INS.LEPI.21			**AY551038**
*Yponomeuta padellus* 5	Malden, Netherlands	*Amelanchier* spec.	ZMA.INS.LEPI.22			**AY551039**
*Yponomeuta padellus* 6	Malden, Netherlands	*Crataegus* spec.	ZMA.INS.LEPI.23			**AY551040**
*Yponomeuta padellus* 7	Malden, Netherlands	*Prunus spinosa*	ZMA.INS.LEPI.24			**AY551041**
*Yponomeuta padellus* 8	Malden, Netherlands	*Sorbus* spec.	ZMA.INS.LEPI.25			**AY551042**
*Yponomeuta padellus* 9	Lithuania	*Crataegus* spec.	ZMA.INS.LEPI.26			**AY551043**
*Yponomeuta padellus* 10	Northern Ireland	*Crataegus* spec.	ZMA.INS.LEPI.27			**AY551044**
*Yponomeuta padellus* 11	De Lutte, Netherlands	*Prunus cerasifera*	ZMA.INS.LEPI.28			**AY551045**
*Yponomeuta padellus* 12	Malden, Netherlands	*Prunus cerasifera*	ZMA.INS.LEPI.29			**AY551046**
*Yponomeuta padellus* 13	Switzerland	*Prunus spinosa*	ZMA.INS.LEPI.30			**AY551047**
*Yponomeuta padellus* 14	Malden, Netherlands	*Crataegus* spec.	ZMA.INS.LEPI.31	**AY543606**	**AY551009**	**AY551049**
*Yponomeuta plumbellus* (Denis & Schiffermüller, 1775)	Bennebroek, Netherlands	*Euonymus europaeus*	ZMA.INS.LEPI.32	**AY543614**	**AY551017**	**AY551057**
*Yponomeuta polystictus* Butler, 1879	Nikko, Japan	*Euonymus sieboldianus*	ZMA.INS.LEPI.33	**AY543626**	**AY551028**	**AY551069**
*Yponomeuta polystigmellus* C. et R. Felder, 1862	Kumoi, Japan	*Euonymus sieboldianus*	ZMA.INS.LEPI.34	**AY543622**	**AY551025**	**AY551065**
*Yponomeuta rorrellus* (Hübner, 1796)	Bergschenhoek, Netherlands	*Salix* spec.	ZMA.INS.LEPI.35	**AY543610**	**AY551013**	**AY551053**
*Yponomeuta sedellus* Treitschke, 1832	Malden, Netherlands	*Sedum telephium*	ZMA.INS.LEPI.36	**AY543615**	**AY551018**	**AY551058**
*Yponomeuta sedellus*	Tokyo, Japan	*Sedum* spec.	ZMA.INS.LEPI.37			**AY551059**
*Yponomeuta sociatus* Moriuti, 1972	Tazawa ko, Japan	*Celastrus orbiculatus*	ZMA.INS.LEPI.38	**AY543621**	**AY551024**	**AY551064**
*Yponomeuta spodocrossus* Meyrick, 1935	Chuzenji-ko, Japan	*Euonymus sacchalinensis*	ZMA.INS.LEPI.39	**AY543620**	**AY551023**	**AY551063**
*Yponomeuta tokyonellus* Matsumura, 1931	Oirase-gawa, Japan	*Euonymus alatus*	ZMA.INS.LEPI.40	**AY543625**	**AY551027**	**AY551068**
*Yponomeuta yanagawanus* Matsumura, 1931	Osaka, Japan	*Euonymus japonica*	ZMA.INS.LEPI.41	**AY543617**	**AY551020**	**AY551060**

### Sequencing

Total DNA was extracted using the protocol of Harrison et al. [27] with the following modifications. Moths were homogenised in 100 µl of a 0.01 M Tris–HCl buffer pH 7.5, containing 0.01 M EDTA, 0.15 M sucrose, and 0.06 M NaCl. Then 100 µl of the following solution was added: 1.25% sodium dodecyl sulphate, 0.1 M EDTA, and 1% v/v of diethylpyrocarbonate in 0.3 M Tris–HCl buffer pH 9. This was incubated for 30 min at 65°C. After this incubation, 40 µl of a 5 M KAc buffer pH = 4.8 was added and the mixture incubated on ice for 45 min. This total DNA was used as template for the amplification of nuclear DNA from the ITS-1 region and for sections of the mitochondrial DNA (mtDNA). From the nuclear DNA, the ITS-1 region was amplified with the primers ‘ITS2’ 5′-GCTGCGTTCTTCATCGATGC-3′ and ‘ITS5’ 5′-GGAAGTAAAAGTCGTAACAAGG-3′. These are universal primers, flanking the ITS-1 in the 5.8S rDNA and the 18S rDNA, respectively [28].

For mtDNA, one section starts with the primer ‘George’ in the cytochrome oxidase subunit I, continues through the tRNA leucine gene and cytochrome oxidase subunit II, and ends in the beginning of the tRNA lysine gene with the primer ‘Eva’. We used the following primer pairs: COI S2792 ‘George’ 5′-ATACCTCGACGTTATTCAGA-3′ combined with COII A3389 ‘Marilyn’ 5′-TCATAAGTTCARTATCATTG-3′, and COII S3138 ‘F’ 5′-GGAGCATCTCCTTTAATAGAACA-3′ with tRNA-Lys A3772 ‘Eva’ 5′-GAGACCATTACTTGCTTTCAGTCATCT-3′. S and A refer to sense and antisense strands, respectively, and numbers refer to the position of the 3′ end. The number is the location on the sequence of *Drosophila yakuba* [29]. Primer ‘F’ was published by Sperling et al. [23] as part of the *Yponomeuta malinellus* sequence; the other three primers were used by Brown et al. [30] and designed by members of the Rick Harrison laboratory at Cornell University on the basis of comparisons of published sequences from *D. yakuba* [29] and *Apis mellifera* [31] as entered in GenBank [32]. For the 16S gene the primers 16Sar 5′-CGCCTGTTTATCAAAAACAT-3′ and 16Sbr 5′-CTCCGGTTTGAACTCAGATC-3′ were used [33].

Using the polymerase chain reaction (PCR) [34], double-stranded amplifications were performed in 25-µl volumes containing the reaction buffer provided with the polymerase, 0.2 mM of each dNTP, 0.2 µM of each primer, 0.1 µg total DNA, and 1 unit Super *Taq* polymerase (Spaero Q) for the mtDNA, and 2.6 units of Expand High Fidelity PCR polymerase (Boehringer Mannheim) for the ITS-1. PCR products were ligated into the pGEM-T Easy vector (Promega) and cloned in *Escherichia coli* JM109 (Promega). Sequencing was performed by using a Hydrolink long-reading gel, with T7 and SP6 as forward and reverse primers, respectively, on a Pharmacia Biotech ALF express automatic sequencer.

### Intraspecific sequence divergence

Intraspecific variability was observed in the ITS-1 sequence, by sequencing it for 14 specimens of *Yponomeuta padellus* and four of *Y. cagnagellus* from different localities and (in the case of the oligophagous *Y. padellus*) different food plants. The variability was restricted to very few differences in sequence length and autapomorphic changes. The data set for the different specimens of these two taxa was phylogenetically completely uninformative, hence the sequence for only one specimen of each taxon is included.

### Analyses

Sequences were entered, edited, and aligned using ClustalX [35], [36]. The final alignments were improved by manual editing. The 16S and COII sequences were easily aligned, with few indels assumed. ITS-1, however, was harder to align because of the much higher variability. It also became clear that part of the sequence had been substituted as a whole in most western Palaearctic taxa. This fragment and the corresponding part in the other taxa were kept as separate blocks in the final alignment, with the characters of one block coded as missing (see ‘*Yponomeuta* nexus file’, Text S1 in the Supplementary Material). No attempt was made to code this substitution event as a separate character, as the final trees for all three sequences already showed these western Palaearctic taxa as a clade. Initial character weights were all equal, except for the 16S sequence. The secondary structure of the 16S rRNA was established by homologising with the published structure of the *Spodoptera frugiperda* large subunit ribosomal RNA [37]. Unpaired bases in all sequences were given a weight of 2, paired bases (with both bases present in the data set) a weight of 1 [38], [39].

Maximum parsimony (MP) analyses were done with PAUP*4.0, version b10 [40]. The search parameters were set to branch-and-bound search or heuristic search with 100 Random Addition Sequences and TBR branch swapping. Bootstrap values were obtained with 1000 replicates with heuristic search and ‘simple’ taxon addition. For the maximum likelihood (ML) analyses, RAxML was used [41], [42] under the default settings for a rapid bootstrap followed by a thorough ML search with 10,000 runs.

For Bayesian inference of the optimal tree topology, the software MrBayes [43], [44], version 3.1.2, was employed. Four runs were made, each to 10^6^ generations, saving every 100th tree. The model used was GTR + Γ + I. Convergence was checked by monitoring the cumulative posterior split probabilities and among-run variability of split frequencies using AWTY on the WWW [45], [46]. The burn-in period was estimated as the period before the average standard deviation of split frequencies decreased to below 0.01, and trees generated during this period were discarded. For all the analyses this moment was reached within 10^6^ generations, except for ITS, which had to be run for 3×10^6^ generations.

The congruence of the different sequences was tested with the Incongruence Length Difference test [[47], [48]; but see [49]] under the same search parameters as for the maximum parsimony analyses, except that 1000 Random Addition Sequences were used and only a single tree was kept.

## Results and Discussion

### Gene trees

#### Maximum parsimony

**Figure 1:**
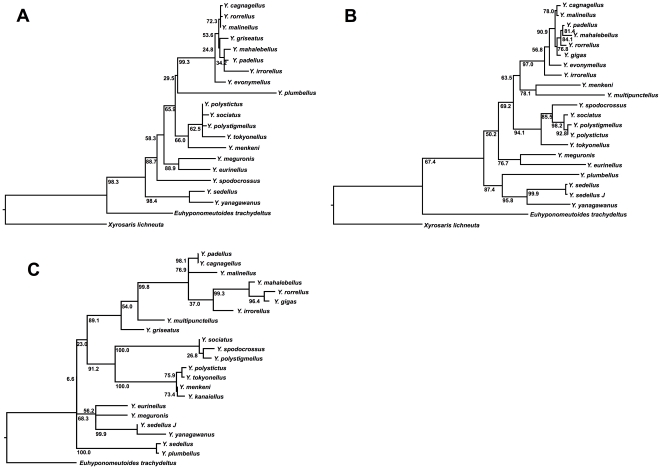
Cladograms of maximum-likelihood gene trees. A, 16S: −ln L = 2823.019097. B, COII: −ln L = 3469.131414. C, ITS-1: −ln L = 3014.056596. Values on branches are bootstrap values.

First, the three sequences were analysed separately (see ‘16S results’ (Figure S1), ‘COII results’ (Figure S2), and ‘ITS-1 results’ (Figure S3) in the Supplementary Material). For 16S (positions 1–576, 58 informative characters, transition/transversion (ti/tv) ratio 0.0000–2.0000; cf. Figure 1 below), the MP analysis resulted in four trees. The majority-rule consensus tree is almost fully resolved; only the relative positions of *Yponomeuta sociatus, Y. polystictus*, and *Y. polystigmellus*, and the resolution within the western Palaearctic clade of Rosaceae-feeding taxa (the *Y. cagnagellus–irrorellus* clade) are not fully determined. Just four of the resolved clades are also supported by relatively high bootstrap values (higher than 70%) [50]: the ingroup, the *Y. sedellus–yanagawanus* clade, the *Y. meguronis–eurinellus* clade, and the *Y. cagnagellus–irrorellus* clade. Upon successive weighting of the characters, four trees were retained. The only changes in the topology of the consensus tree are in the western Palaearctic clade. The same clades as in the unweighted analysis are supported by bootstrapping, in addition to the clade containing *Y. tokyonellus*.

With COII (positions 577–1591, 129 informative characters, ti/tv ratio 0.2000–6.0000), the MP analysis gave eight trees. The only polytomy on the majority-rule consensus tree is an almost basal one between *Y. menkeni*, the *Y. cagnagellus–irrorellus* clade, and a clade consisting of all other ingroup species except *Y. multipunctellus*, which is the most basal species. Unlike in the 16S tree, many branches are supported by bootstrap values higher than 70%. The two specimens of *Y. sedellus* (one from Japan, the other from The Netherlands—only the latter was sequenced for 16S) were each other's sisters, as expected if the species is not paraphyletic. Successive weighting retained one of the eight trees.

Remarkably, the positions of *Y. padellus, Y. cagnagellus*, and *Y. malinellus* do not agree with the dendrogram presented by Sperling et al. [23] based on the same gene, albeit a longer sequence. Re-analysing their data, we found that a parsimony analysis of the full sequences for these three taxa with *Y. multipunctellus* as outgroup gave the tree reported by them, both using their full sequences (2347 bp for the ingroup taxa) and the 1014 bp corresponding to the sequence we analysed. Combining their sequences and ours gave basically the same tree as only our data, but with their *Y. padellus* and *Y. cagnagellus* as a clade in a polytomy with our *Y. malinellus* and *Y. cagnagellus*, and their *Y. malinellus* in a polytomy with *Y. padellus, Y. mahalebellus*, and *Y. rorrellus*, at least in the majority-rule consensus of the 12 most parsimonious trees and the three slightly different topologies obtained after successive weighting. The *Dra*1 restriction site (used by Sperling et al. [23]), which differentiated between their *Y. padellus* and *Y. cagnagellus* on one hand, and *Y. malinellus* on the other (in which it was present), is present in our data in the taxa *Y. padellus, mahalebellus, rorrellus*, and *gigas*, but not in *Y. cagnagellus* or *Y. malinellus*. The *Bcl*1 site (absent in their sequence of *Y. malinellus*) is absent in our *Y. padellus, mahalebellus, rorrellus, yanawaganus*, and *eurinellus*, Thus, for our sequences these two restriction sites are not diagnostic between *Y. malinellus* and *Y. cagnagellus* + *Y. padellus*, but rather between *Y. padellus* and the other two taxa. Assuming the sequences were read correctly by both Sperling et al. and by us, these restriction sites might be more variable in the Old World than in the New, possibly as a result of a founder effect. Only more extensive sampling can confirm this hypothesis.

MP analysis of the ITS-1 sequences (positions 1592–2454, 169 informative characters, ti/tv ratio 0.6000–7.0000) gave 32 trees, one of which was retained after successive weighting. Many branches received high bootstrap support with the unweighted data. The position of the outgroup, at the branch leading to the *Y. polystigmellus* clade, is different from that in the 16S and COII analyses. However, this node is just weakly supported with a bootstrap value of <50%. In the ITS-1 trees, the two *Y. sedellus* specimens do not form a clade or even a grade. This unexpected result might be due to introgression, the more so because the Japanese accession consistently comes out of the analyses as sister to another Japanese species, *Y. yanagawanus*, while the Dutch specimen always groups with the Dutch accession of *Y. plumbellus*.

#### Maximum likelihood

The three sequences were further analysed using maximum likelihood (ML) inference. The model parameters calculated by RAxML are given in the ‘*Yponomeuta* nexus file’ (Text S1) in the Supplementary Material.

The tree topology obtained using the 16S gene (Figure 1A) differs from the MP tree in the position of *Y. spodocrossus* (more basal in the MP result). Other differences are poorly supported in both results. Only six clades are supported by bootstrap values>70%, of which five also have high bootstrap values in the MP analysis.

For COII, the resulting tree (Figure 1B) differs from the MP result only in the position of the outgroup, below the *Y*. *plumbellus–sedellus* clade rather than on the branch leading to *Y. multipunctellus*, and in the resolution of the *Y. meguronis–eurinellus* clade. The ML tree is identical to the reweighted COII MP tree.

With ITS-1, *Xyrosaris lichneuta* consistently shows up as sister to the *Y. cagnagellus–irrorellus* clade (data not shown). Inspection of the tree showed the branch leading to *X. lichneuta* to be very long, so this position is probably the result of long-branch attraction. Bootstrap support for this position is also very low at 14%. Deleting the species from the data set did not change the topology for the remaining species. The resulting tree (Figure 1C) differs from the MP result in the position of *Y. multipunctellus* and *Y. griseatus*, which branch off sequentially rather than as a clade, and in the position of the outgroup, which branches off below the *Y. plumbellus–sedellus* clade rather than below the *Y. polystigmellus–sociatus* clade. However, the position of the clade *Y. kanaiellus–sociatus* in the ML tree is only very weakly supported. Again, the two accessions of *Y. sedellus* do not form a grade or clade. The resolutions of the polytomies of the MP tree are only very weakly supported.

#### Bayesian inference

The sequence data were also subjected to Bayesian inference (BI). The 16S data resulted in the majority-rule consensus tree described in the Supplementary Material (‘16S results’, Figure S1). The runs converged on the same consensus. The tree is very similar in topology to the ML and the MP majority-rule trees. The clade confidence estimates are higher than the MP bootstrap values.

The support for the differently resolved clades in the MP, ML, and BI trees is usually lower than that for the clades on which all three analyses agree (cf. [49]). All analyses agree that *Y. sedellus* and *Y. yanagawanus* form a clade that is sister to the other species. Among the remaining taxa, the *Y. cagnagellus–irrorellus* clade, two nodes in the *Y*. *menkeni–sociatus* clade, and *Y. meguronis* + *Y*. *eurinellus* are always resolved.

For COII and ITS-1, the data were analysed in the same way as for 16S. Again, the trees resemble the MP and the ML results (see Supplementary Material, ‘COII results’, Figure S2 and ‘ITS-1 results’, Figure S3), with the differences being confined to poorly supported clades. With ITS-1, *Xyrosaris lichneuta* showed the same behaviour as before, so it was deleted from the data set.

### Species trees

To test whether the different partitions trace the same evolutionary history (or at least trace statistically indistinguishable histories) and can therefore fruitfully be combined in a total-evidence approach [26], we applied the Incongruence Length Difference test (ILD, [47], [48]) to the MP analysis using heuristic search. To begin with, the ILD test was run on the total data set partitioned into the three different gene sequences. The resulting *p* value (999 randomisations) is 0.001. The same test was then run on the three possible two-sequence subsets of the total data set. Only the subset consisting of the two mitochondrial genes shows some congruence between the partitions (at *p* = 0.045). Therefore, the incongruence can be ascribed to the (nuclear) ITS-1 sequence. Because weighting the characters might influence the congruence of the different partitions [51], [52], the ILD test was repeated with the character weights set to the values obtained after successive weighting of the set of active characters or of the individual sequences. The probability increases from *p* = 0.045 to 0.149 with the successively weighted data. When the weights are set to the values obtained for the individual genes, all combinations remain incongruent at *p* = 0.001, which is not surprising as the incongruence is then reinforced rather than diminished.

Such incongruence does not necessarily indicate different histories for the separate partitions: several authors [53]–[55] have argued that different proportions of uninformative characters in the partitions can lead to the ILD test showing them to be incongruent. We therefore repeated the ILD tests with all uninformative characters deleted, but the results did not change. The complete data set, and both combinations of one mitochondrial gene and the nuclear ITS-1 gene remain incongruent at the 0.1% level, and the *p* value for the mitochondrial genes remains at 0.04 for the unweighted data, but decreases to 0.02 for the successively weighted data.

The MP analysis of the mitochondrial genes together resulted in one tree (see Supplementary Material, ‘Mitochondrial results’, Figure S4), and likewise the total-evidence data set (‘Total-evidence results’, Figure S5). The tree is stable to successive weighting. The pattern shown by the individual sequences is, not surprisingly, confirmed, with the same clades well supported. The result resembles the COII tree more than the 16S tree, which can be ascribed to the larger size of the COII gene.

**Figure 2:**
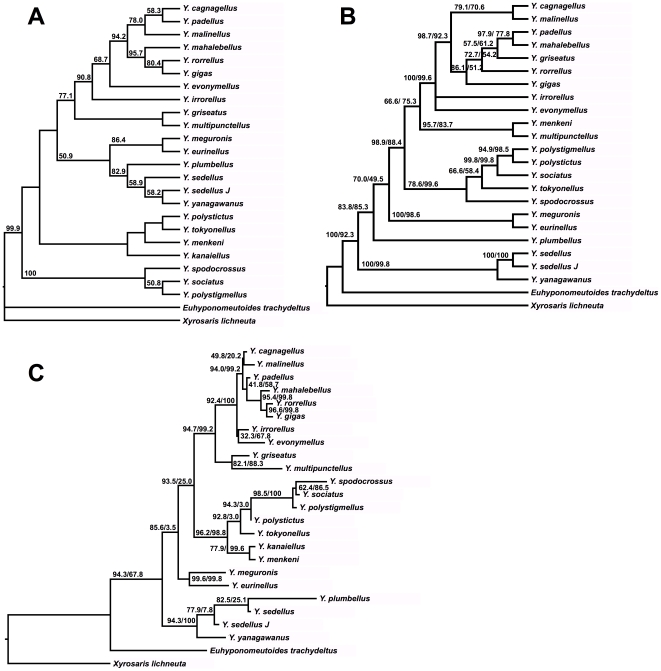
Species trees. A, MP tree; l = 1198, CI = 0.647, RI = 0.641; B, BI mitochondrial tree; C, total-evidence ML tree, −ln L = 17485.271575. Values on branches are MP (A) or ML (B, C) bootstrap values/BI clade confidence values.

An analysis of the total data set, done despite the result of the ILD test [see also 49], [53], [56], resulted in one tree (Figure 2A). The tree is stable to successive weighting. The strongly supported clades are the same as in the mitochondrial and individual sequence analyses, with the exception of a clade consisting of the *Yponomeuta cagnagellus–irrorellus* and a *Y. griseatus–multipunctellus* clade. This clade is also seen in the results for ITS-1, which is the only sequence for which both latter species are accessed. The weakly supported clades are those upon which the individual sequences do not agree either. In particular, the positions of some individual taxa and the relative positions of the *Y. cagnagellus–irrorellus* clade, the *Y*. *meguronis–eurinellus* clade, the *Y*. *plumbellus–sedellus* clade, and the *Y. kanaiellus–tokyonellus* clade, are not well supported.

Applying both ML and BI to the mitochondrial data set resulted in the tree shown in Figure 2B, and applying both to the total data set (without the ITS-1 sequence for *Xyrosaris lichneuta*) gave the tree in Figure 2C. The only differences are that under ML, *Y. padellus* up to *Y. gigas*, and *Y. irrorellus* + *Y. evonymellus* form clades, but with low bootstrap support. The BI search was started using a General Time Reversible + gamma model for all three partitions, which were allowed to evolve independently. For the mitochondrial and the total-evidence trees, the results are almost identical to the MP trees, but in both cases with the outgroups attached to the *Y. yanagawanus–sedellus* clade, and with *Y. tokyonellus* and *Y. spodocrossus* reversed. One possible interpretation of this result is that the outgroup is too distant to resolve the root of the otherwise stable ingroup topology.

### Conclusions on tree topology

**Figure 3:**
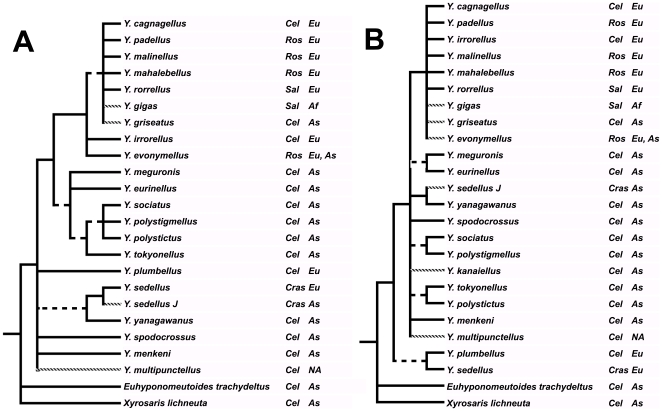
Adams consensus trees of all results (MP, MP with successive reweighting, ML). A, 16S + COII (19 trees), and B, all trees (53 trees). Dashed lines indicate grades; hatched lines show taxa not present in all partitions. Following their names are species' host plant and distribution. Cel  =  Celastraceae, Ros  =  Rosaceae, Sal  =  Salicaceae, Cras  =  Crassulaceae; Eu  =  western Palaearctic, As  =  eastern Palaearctic, Af  =  Africa, NA  =  North America.

The different kinds of analysis for the three sequences all point to similar tree topologies, only differing in the weakly supported placement of some taxa or clades for which the data are ambiguous. The unambiguous parts show up in the Adams consensus trees that we reconstructed from the optimal trees of the different analyses (MP, MP with successive weighting, ML–Figure 3). Adams consensus trees retain the undisputed skeleton of the basal trees; the taxa on which no agreement is reached are placed in polytomies at the base of the clades to which they belong. Such Adams consensus trees should be read as follows: taxa from a ‘soft’ polytomy (one not occurring on all basal trees) upward form a monophyletic clade, while taxa above such a polytomy are either a clade or a paraphyletic grade. In nomenclatorial terms, the taxa in a soft polytomy belong to a clade, but are ‘incertae sedis’ within that clade. Thus, Adams consensus trees are similar to the repeatability criterion developed by Chen et al. [49], but take into account the effect of ‘rogue’ taxa, which break up any strict repeatability of clades.

The Adams consensus of all trees for 16S and COII (taxa that occur in only one of the data sets were placed in the position indicated by the data set in which they are present) gives an estimate of the mitochondrial tree (Figure 3A). Next to the *Y. cagnagellus–irrorellus* clade, there are several grades, among which are a *Y. sedellus–yanagawanus* and a *Y. sociatus*–*Y. polystictus* grade. The Adams consensus tree over all results for all three genes is less resolved, but still retains the Rosaceae-feeding clade (Figure 3B).

### Host-plant associations and biogeography

The evolution of the association with host plants, and of the biogeographic history, can already be reconstructed using the topologies shown in Figures 3A and B, even though these are quite unresolved. Mapping the host-plant association and the biogeography onto the total-evidence cladograms makes no difference for the conclusions. Most species of *Yponomeuta* lay their eggs and in the larval stage feed on Celastraceae. Exceptions are *Y. sedellus*, which feeds on various *Sedum* species (Crassulaceae), and the clade *Y. cagnagellus–irrorellus*, which feeds in large part on Rosaceae or Salicaceae (*Y. rorrellus* and *Y. gigas*); *Y. irrorellus* and *Y. cagnagellus*, however, still (or again) feed on Celastraceae. Mapping the host-plant data shows, despite the polytomies still present in Figures 3A and B, that the ancestral host plants are Celastraceae (this is corroborated by the results by Ulenberg [21] on morphological data for all species in the genus (her Figure 5) and on data for all genera in the subfamily: *Yponomeuta* belongs to a clade of genera whose ancestral host association is Celastraceae). *Yponomeuta sedellus* is unique in having shifted to Crassulaceae. The host shifts that can be reconstructed are from the ancestral Celastraceae to Rosaceae in the ancestor of the *Y*. *cagnagellus–irrorellus* clade, with reversals back to Celastraceae in *Y. cagnagellus* and *Y. irrorellus*. Sensitivity to the plant compound benzaldehyde (common in Rosaceae—Rosaceae-feeders are sensitive to this compound—but absent from Celastraceae—Celastraceae-feeders are insensitive) in *Y*. *cagnagellus* is supportive of a former association with Rosaceae and therefore its present association with Celastraceae might very well be a backshift [57].

Alternatively, the shift to Rosaceae might have taken place after *Y. irrorellus* and the ancestor of the remainder of the clade split up, as parallel developments in *Y. evonymellus* and in the clade *Y. cagnagellus–griseatus*, but still with a reversal in *Y. cagnagellus*. A further shift, to Salicaceae, took place in the common ancestor of *Y. rorrellus* and *Y. gigas* if they really form a clade. Their close relationship is indicated by the ITS-1 data and also proposed by morphological taxonomists [e.g., 13], and further supported by the very low variability levels at allozyme loci, suggesting one or more severe bottlenecks in the common ancestor of the two species (Menken, [58] and unpubl.), as well as the aberrant sex pheromone of *Y*. *rorrellus* (the pheromone of *Y*. *gigas* is unknown) which is also supportive of such a bottleneck [59]. If they form a grade, as shown by the COII data, the shift to Salicaceae either occurred twice, or once in the common ancestor of these two species and *Y. padellus* and *Y. mahalebellus*, resulting in either a polymorphic ancestral species for this trait or in a reversal in the common ancestor of *Y. padellus* and *Y. mahalebellus*.

We also reconstructed the history of the association with the software Lagrange [60], but this program requires fully resolved cladograms. We therefore investigated the Bayesian mitochondrial (see Supplementary Material, ‘Mitochondrial host Lagrange results’, Text S2) and total-evidence (‘Total-evidence host Lagrange results’, Text S3) trees only. The cladograms were coded as ultrametric trees, with branch lengths of (multiples of) one. All host shifts were coded as equally likely over the entire duration of the phylogenetic tree, and ancestral ranges were restricted to a maximum of two hosts. The shift to Rosaceae possibly occurred as early as the common ancestor of the Rosaceae-feeding clade. However, this ancestor being restricted to Celastraceae has a slightly higher likelihood (−lnL 0.3907 vs. 0.3789); the shift is then reconstructed as having taken place in *Y. evonymellus* and in the common ancestor of the *cagnagellus–rorrellus* clade. The shift to Crassulaceae is reconstructed as having taken place (as a broadening of the feeding range) in the common ancestor of *Y. sedellus* (both accessions) and *Y. plumbellus*, each accession having lost one of its ancestral host plants. The shift to Salicaceae is again reconstructed as having taken place in the ancestor of *Y. rorrellus* and *Y. gigas*.

It is clear that at the genus level no co-evolution but sequential evolution occurred (i.e., the tracking of resources); however, co-evolution between *Yponomeuta* species and East Asian *Euonymus* species cannot be excluded. This might be investigated constructing a phylogeny of *Euonymus* and timing the two phylogenies using a molecular clock [e.g., 61].

Mapping the presence of the species in East Asia, the western Palaearctic, and North America onto the cladograms of Figures 3A and B shows that the genus probably originated in East Asia. *Yponomeuta sedellus* and *Y. plumbellus* (or their common ancestor), and the common ancestor of the *Y*. *cagnagellus–irrorellus* clade then dispersed to the western Palaearctic and the Canary Islands (*Y. gigas*), and *Y. multipunctellus* to North America. The alternative, that the common ancestor was widespread, is less parsimonious when one takes into account that the *Y. cagnagellus–irrorellus* clade is never basal, thus requiring several extinctions in the western Palaearctic of clades presently confined to East Asia. The genus is also present in Southeast Asia, Australia, and continental Africa, but results obtained by Ulenberg [21] show that the species occurring in these areas are all members of two basal clades, and that the Eurasian species are an apical, monophyletic group.

An analysis with Lagrange gives more or less the same results: for the mitochondrial tree (see Supplementary Material, ‘Mitochondrial distribution Lagrange results’, Text S4) the most likely scenario is a dispersal of the common ancestor of the *Y*. *cagnagellus–irrorellus* clade to the western Palaearctic, and a subsequent dispersal of *Y. gigas* to the Canary Islands. The common ancestor of *Y. evonymellus* and *Y. irrorellus* expanded its range to include East Asia again, where the latter species went extinct. *Y. griseatus* also dispersed back to East Asia. The ancestor of the two *Y. sedellus* accessions expanded its range, while each of the terminals went extinct in part of the ancestral range. *Yponomeuta plumbellus* dispersed from Asia to the western Palaearctic. Under the total-evidence tree scenario (‘Total-evidence distribution Lagrange results’, Text S5) the most likely reconstruction is an expansion of the range of the common ancestor of the *Y*. *cagnagellus–irrorellus* clade and of the common ancestor of the *Y. plumbellus–sedellus* clade to the western Palaearctic, and subsequent extinction of *Y. irrorellus* and the common ancestors of the *Y. cagnagellus–padellus* clade and *Y. plumbellus + sedellus* in the Far East, and of the Japanese terminal in the western Palaearctic.

In conclusion, palaearctic *Yponomeuta* probably arose as an East Asian clade feeding on Celastraceae, and subsequently expanded its distribution area westward to the western Palaearctic and the Canary Islands, and to North America. One of the species moving west, the ancestor of the *Y. cagnagellus–irrorellus* clade, also broadened its host range to include Rosaceae (and further on to Salicaceae). The sensitivity to benzaldehyde noted above for *Y. cagnagellus* may have arisen in this ancestor, allowing it to radiate on Rosaceae The only oligophagous species, *Y*. *padellus,* belongs to the derived western Palaearctic clade; its position amidst monophagous species is another proof that diet specialisation is not a dead end of evolution (see [62] and references therein).

## Supporting Information

Figure S1 16S results. Results of analyses using 16S not given in main figures. A. 16S majority-rule tree of 4 trees (l = 330, ci = 0.670, ri = 0.671). Above branch: frequency <100%; below branch: bootstrap value >50%. B. 16S successive weighting majority-rule tree of 4 trees (l = 102.09773, ci = −0.883, ri− = 0.847). Above branch: frequency <100%; below branch: bootstrap value >50%. C. 16S Bayesian analysis tree. Below branch: posterior probability >50%. D. 16S Adams consensus tree of parsimony and likelihood results.
(2.22 MB TIF)

Figure S2 COII results. Results of analyses using COII not given in main figures. A. COII majority-rule tree of 8 trees (l = 844, ci = 0.645, ri = 0.691). Above branch: frequency <100%; below branch: bootstrap value >50%. B. COII successive weighting tree (l = 203.456575, ci = −0.861, ri− = 0.865). Below branch: bootstrap value >50%. C. COII Bayesian analysis tree. Below branch: posterior probability >50%. D. COII Adams consensus tree of parsimony and likelihood results.
(2.21 MB TIF)

Figure S3 ITS-1 results. Results of analyses using ITS-1 not given in main figures. A. ITS-1 majority-rule tree of 32 trees (l = 984, ci = 0.752, ri = 0.753). Above branch: frequency <100%; below branch: bootstrap value >50%. B. ITS-1 successive weighting tree (l = 307.87836, ci = −0.906, ri− = 0.891). Below branch: bootstrap value >50%. C. ITS-1 Bayesian analysis tree (Xyrosaris lichneuta excluded). Below branch: posterior probability >50%. D. ITS-1 Adams consensus tree of parsimony and likelihood results.
(2.31 MB TIF)

Figure S4 Mitochondrial results. Results of analyses using 16S and COII not given in main figures. A. Mitochondrial maximum parsimony tree (l = 1195, ci = 0.640, ri = 0.670). Below branch: bootstrap value >50%. B. Mitochondrial successive weighting tree (l = 304.14502, ci = −0.861, ri− = 0.841). Below branch: bootstrap value >50%. C. Mitochondrial maximum likelihood tree (−ln L = −5266.057315). Below branch: bootstrap value >50%. D. Mitochondrial Bayesian inference tree. Below branch: posterior probability >50%.
(2.02 MB TIF)

Figure S5 Total-evidence results. Results of total-evidence analyses not given in main figures. A. Total-evidence maximum parsimony tree (l = 2322, ci = 0.648, ri = 0.643). Below branch: bootstrap value >50%. B. Total-evidence successive weighting tree (l = 573.01014, ci = −0.879, ri− = 0.850). Below branch: bootstrap value >50%. C. Total-evidence maximum likelihood tree (−ln L = 17485.271575). Below branch: bootstrap value >50%. D. Total-evidence Bayesian inference tree. Below branch: posterior probability >50%.
(3.07 MB TIF)

Text S1 Yponomeuta nexus file. Nexus file of aligned data, including maximum-likelihood parameters obtained using RAxML.
(0.14 MB DOC)

Text S2 Mitochondrial host Lagrange results. Evolution of host range based on mitochondrial Bayesian analysis tree.
(0.05 MB DOC)

Text S3 Total-evidence host Lagrange results. Evolution of host range based on total-evidence Bayesian analysis tree.
(0.05 MB DOC)

Text S4 Mitochondrial distribution Lagrange results. Evolution of biogeographical range based on mitochondrial Bayesian analysis tree.
(0.05 MB DOC)

Text S5 Total-evidence distribution Lagrange results. Evolution of biogeographical range based on total-evidence Bayesian analysis tree.
(0.05 MB DOC)
