# Supplementary figures and images for: Molecular Phylogeny of the Small Ermine Moth Genus Yponomeuta (Lepidoptera, Yponomeutidae) in the Palaearctic
Source: PLoS One. 2010 Mar 29;5(3):e9933. doi: 10.1371/journal.pone.0009933 (PMC2847947; doi:10.1371/journal.pone.0009933)

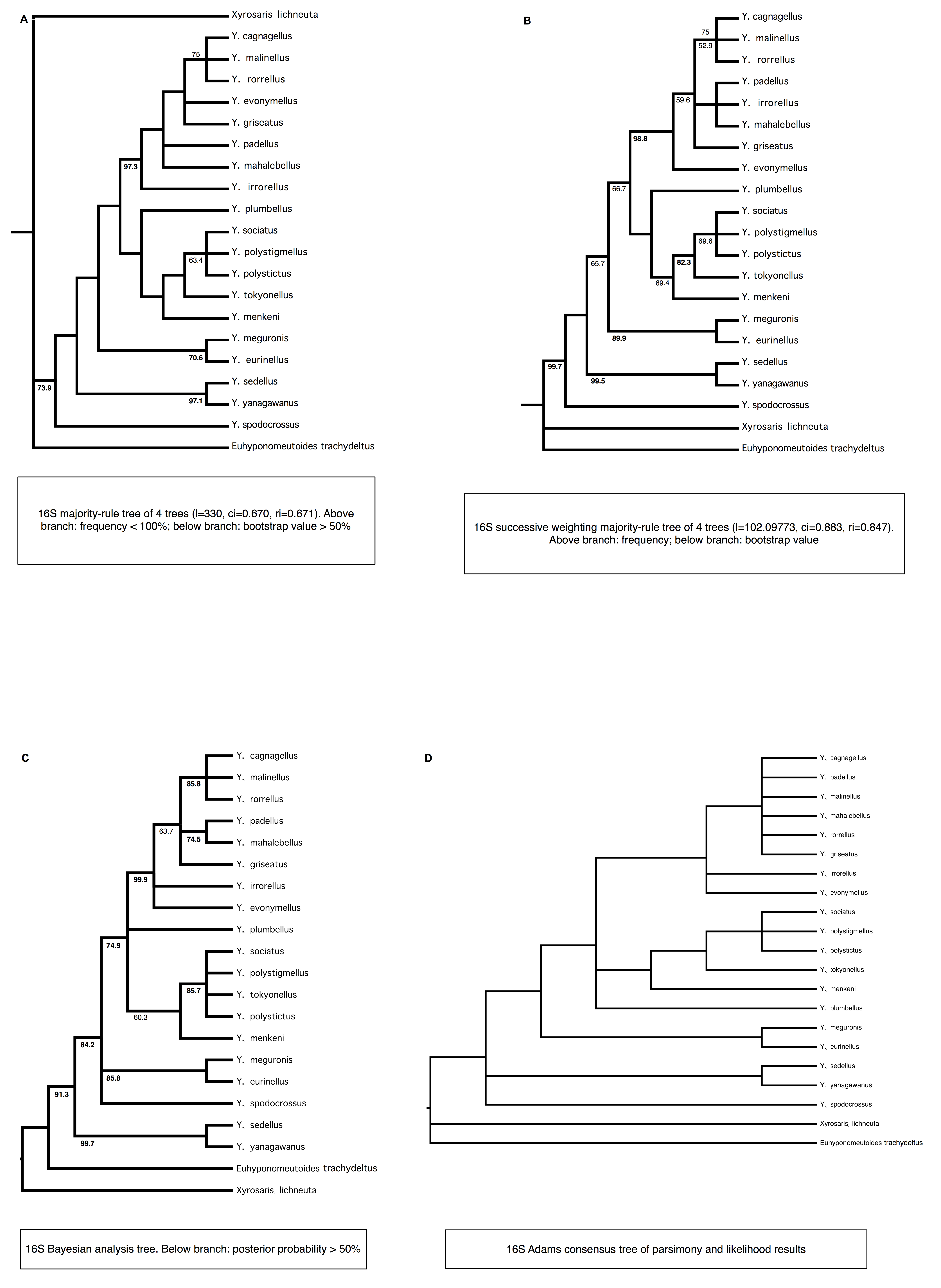

Supplement: Figure S1 — 16S results. Results of analyses using 16S not given in main figures. A. 16S majority-rule tree of 4 trees (l = 330, ci = 0.670, ri = 0.671). Above branch: frequency <100%; below branch: bootstrap value >50%. B. 16S successive weighting majority-rule tree of 4 trees (l = 102.09773, ci = −0.883, ri− = 0.847). Above branch: frequency <100%; below branch: bootstrap value >50%. C. 16S Bayesian analysis tree. Below branch: posterior probability >50%. D. 16S Adams consensus tree of parsimony and likelihood results. (2.22 MB TIF) [file pone.0009933.s001.tif]

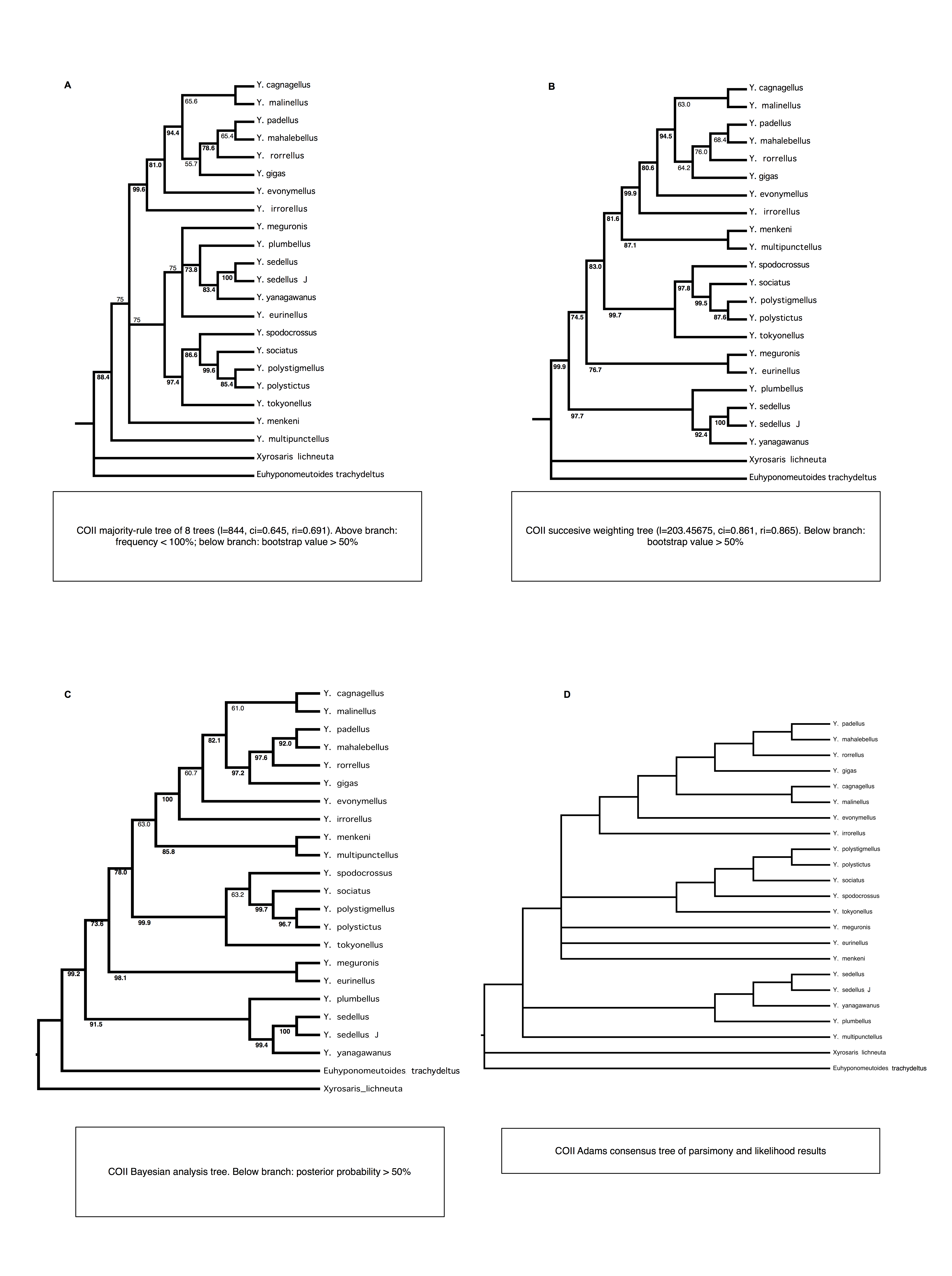

Supplement: Figure S2 — COII results. Results of analyses using COII not given in main figures. A. COII majority-rule tree of 8 trees (l = 844, ci = 0.645, ri = 0.691). Above branch: frequency <100%; below branch: bootstrap value >50%. B. COII successive weighting tree (l = 203.456575, ci = −0.861, ri− = 0.865). Below branch: bootstrap value >50%. C. COII Bayesian analysis tree. Below branch: posterior probability >50%. D. COII Adams consensus tree of parsimony and likelihood results. (2.21 MB TIF) [file pone.0009933.s002.tif]

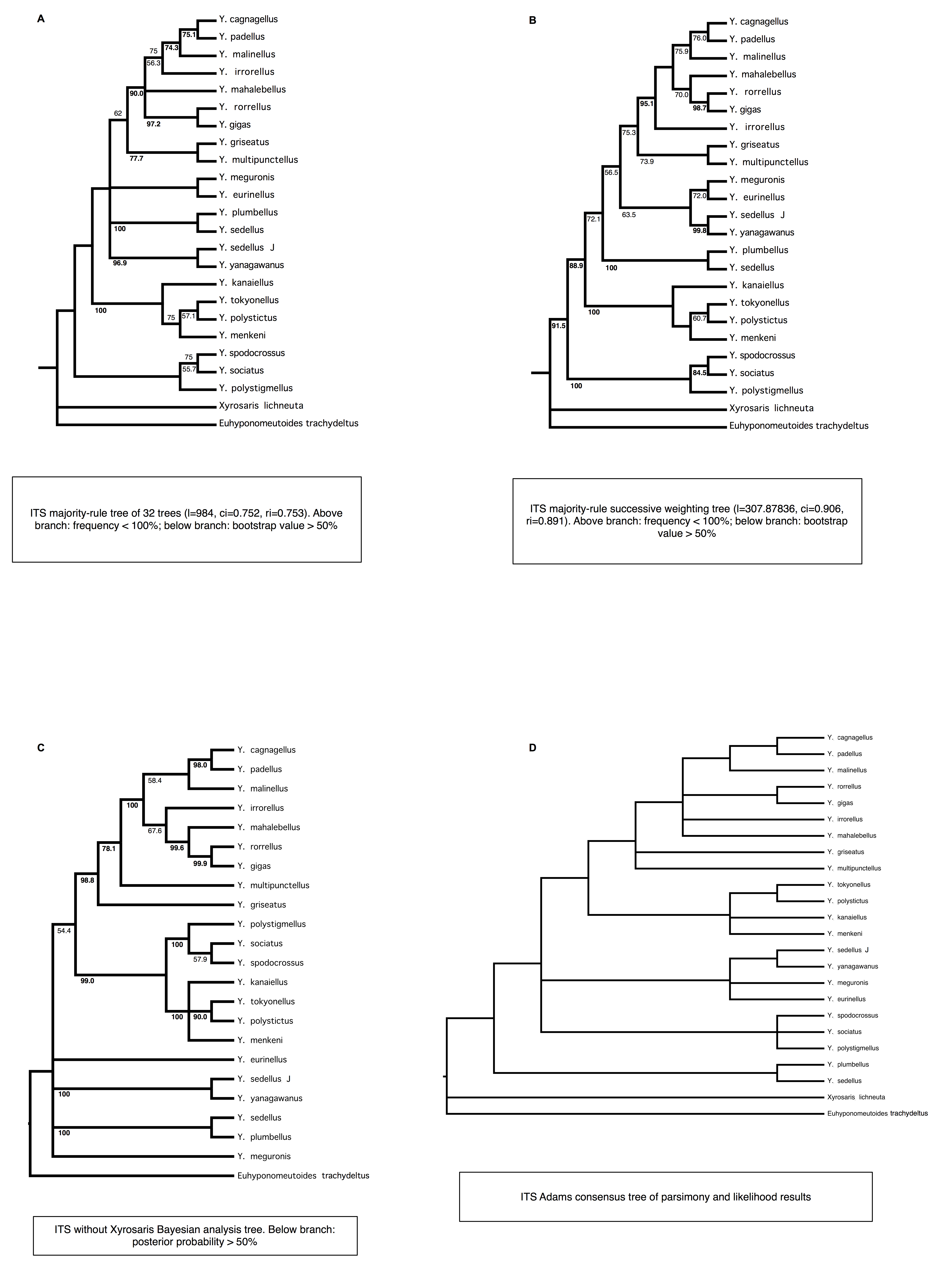

Supplement: Figure S3 — ITS-1 results. Results of analyses using ITS-1 not given in main figures. A. ITS-1 majority-rule tree of 32 trees (l = 984, ci = 0.752, ri = 0.753). Above branch: frequency <100%; below branch: bootstrap value >50%. B. ITS-1 successive weighting tree (l = 307.87836, ci = −0.906, ri− = 0.891). Below branch: bootstrap value >50%. C. ITS-1 Bayesian analysis tree (Xyrosaris lichneuta excluded). Below branch: posterior probability >50%. D. ITS-1 Adams consensus tree of parsimony and likelihood results. (2.31 MB TIF) [file pone.0009933.s003.tif]

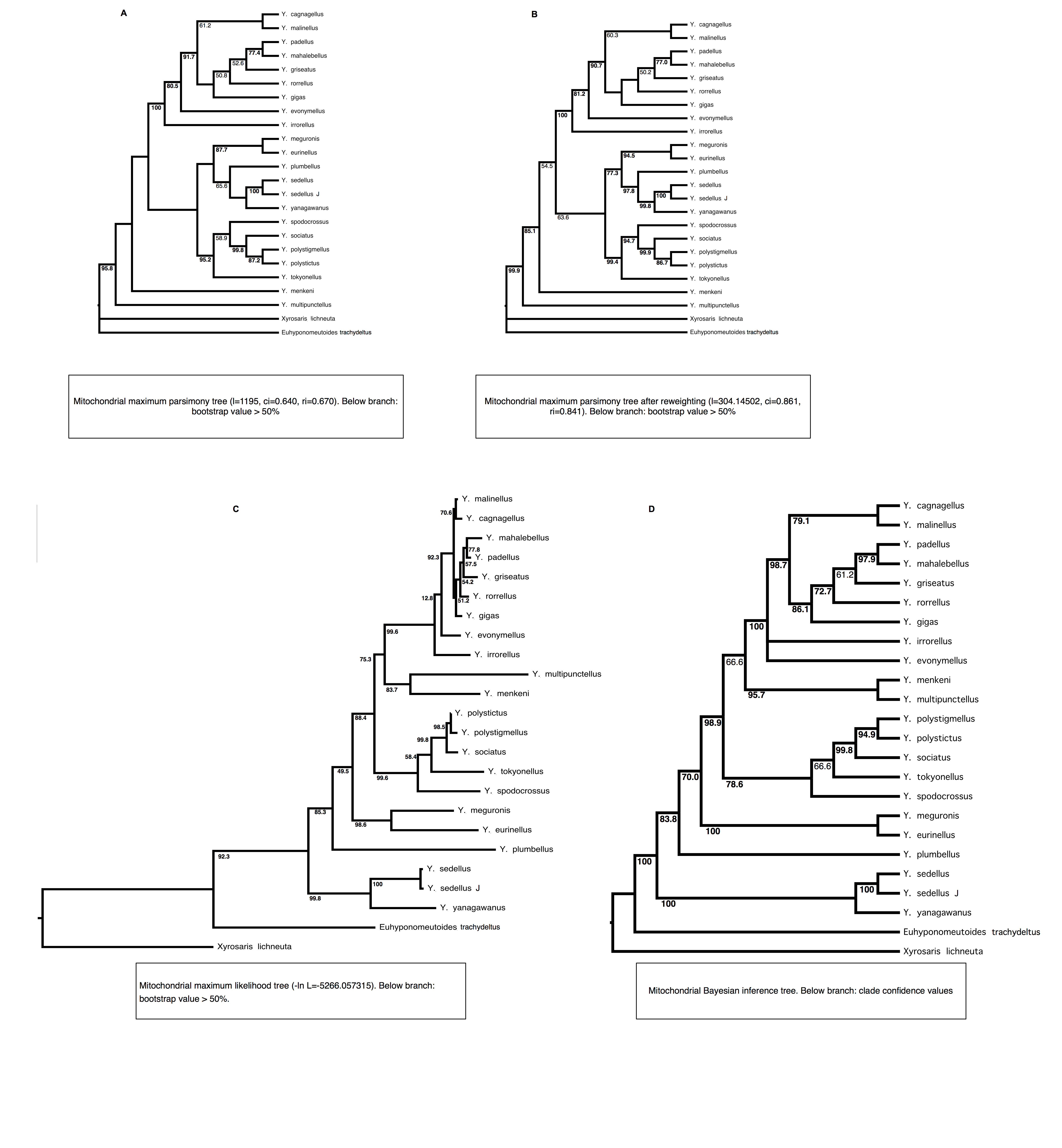

Supplement: Figure S4 — Mitochondrial results. Results of analyses using 16S and COII not given in main figures. A. Mitochondrial maximum parsimony tree (l = 1195, ci = 0.640, ri = 0.670). Below branch: bootstrap value >50%. B. Mitochondrial successive weighting tree (l = 304.14502, ci = −0.861, ri− = 0.841). Below branch: bootstrap value >50%. C. Mitochondrial maximum likelihood tree (−ln L = −5266.057315). Below branch: bootstrap value >50%. D. Mitochondrial Bayesian inference tree. Below branch: posterior probability >50%. (2.02 MB TIF) [file pone.0009933.s004.tif]

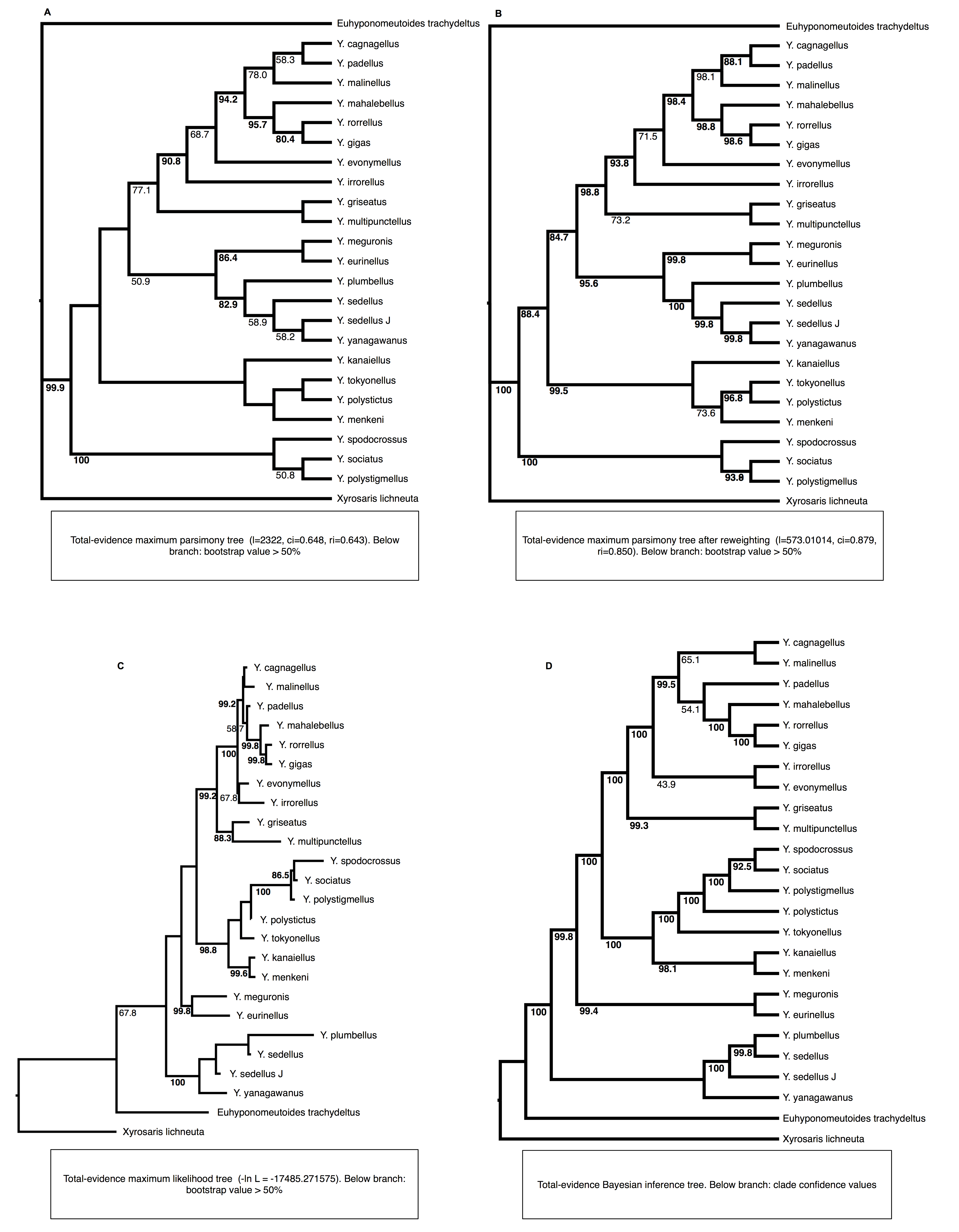

Supplement: Figure S5 — Total-evidence results. Results of total-evidence analyses not given in main figures. A. Total-evidence maximum parsimony tree (l = 2322, ci = 0.648, ri = 0.643). Below branch: bootstrap value >50%. B. Total-evidence successive weighting tree (l = 573.01014, ci = −0.879, ri− = 0.850). Below branch: bootstrap value >50%. C. Total-evidence maximum likelihood tree (−ln L = 17485.271575). Below branch: bootstrap value >50%. D. Total-evidence Bayesian inference tree. Below branch: posterior probability >50%. (3.07 MB TIF) [file pone.0009933.s005.tif]
