# Supplementary material for: Molecular Phylogeny of the Small Ermine Moth Genus Yponomeuta (Lepidoptera, Yponomeutidae) in the Palaearctic
Source: PLoS One. 2010 Mar 29;5(3):e9933. doi: 10.1371/journal.pone.0009933 (PMC2847947; doi:10.1371/journal.pone.0009933)
Supplement: Text S1 — Yponomeuta nexus file. Nexus file of aligned data, including maximum-likelihood parameters obtained using RAxML. (0.14 MB DOC) [file pone.0009933.s006.doc]

#NEXUS

BEGIN DATA;

DIMENSIONS NTAX=41 NCHAR=2454;

FORMAT DATATYPE=DNA MISSING=? GAP=- ;

MATRIX

[ 10 20 30 40 50 60 70 80 90 100 110 120 130 140 150 160 170 180 190 200 210 220 230 240 250 260 270 280 290 300 310 320 330 340 350 360 370 380 390 400 410 420 430 440 450 460 470 480 490 500 510 520 530 540 550 560 570 580 590 600 610 620 630 640 650 660 670 680 690 700 710 720 730 740 750 760 770 780 790 800 810 820 830 840 850 860 870 880 890 900 910 920 930 940 950 960 970 980 990 1000 1010 1020 1030 1040 1050 1060 1070 1080 1090 1100 1110 1120 1130 1140 1150 1160 1170 1180 1190 1200 1210 1220 1230 1240 1250 1260 1270 1280 1290 1300 1310 1320 1330 1340 1350 1360 1370 1380 1390 1400 1410 1420 1430 1440 1450 1460 1470 1480 1490 1500 1510 1520 1530 1540 1550 1560 1570 1580 1590 1600 1610 1620 1630 1640 1650 1660 1670 1680 1690 1700 1710 1720 1730 1740 1750 1760 1770 1780 1790 1800 1810 1820 1830 1840 1850 1860 1870 1880 1890 1900 1910 1920 1930 1940 1950 1960 1970 1980 1990 2000 2010 2020 2030 2040 2050 2060 2070 2080 2090 2100 2110 2120 2130 2140 2150 2160 2170 2180 2190 2200 2210 2220 2230 2240 2250 2260 2270 2280 2290 2300 2310 2320 2330 2340 2350 2360 2370 2380 2390 2400 2410 2420 2430 2440 2450 ]

[ . . . . . . . . . . . . . . . . . . . . . . . . . . . . . . . . . . . . . . . . . . . . . . . . . . . . . . . . . . . . . . . . . . . . . . . . . . . . . . . . . . . . . . . . . . . . . . . . . . . . . . . . . . . . . . . . . . . . . . . . . . . . . . . . . . . . . . . . . . . . . . . . . . . . . . . . . . . . . . . . . . . . . . . . . . . . . . . . . . . . . . . . . . . . . . . . . . . . . . . . . . . . . . . . . . . . . . . . . . . . . . . . . . . . . . . . . . . . . . . . . . . . . ]

Y.cagnagellus1 -------------------------------------------------------------------------------------------------------------------------------------------------------------------------------------------------------------------------------------------------------------------------------------------------------------------------------------------------------------------------------------------------------------------------------------------------------------------------------------------------------------------------------------------------------------------------------------------------------------------------------------------------------------------------------------------------------------------------------------------------------------------------------------------------------------------------------------------------------------------------------------------------------------------------------------------------------------------------------------------------------------------------------------------------------------------------------------------------------------------------------------------------------------------------------------------------------------------------------------------------------------------------------------------------------------------------------------------------------------------------------------------------------------------------------------------------------------------------------------------------------------------------------------------------------------------------------------------------------------------------------------------------------GCTGCGTTCTTCATCGATGCGCGAGCCAAGTGATCCACCGTCCAGGGTGATGATTTGC--CGTTTGGCGTTGATCGAAATTGAGAG--AAAAAAATTCTACATACACCTAGGTGATAT--GTGTGCACGTGCGTGCGTG----TG-------------------------------------------------------------------------------------------------------------------------TAACGACAAACGAGCGCTCTC--ACGC---CGTCTCTCACG---------ACG-C--GAGGCGC-TCGC-CATCACCGCATAATA-------------------------------------------T--A-TCG-GCACGTGCGACTCCGGGCGCGGATTTTG-AACCCATCGATGTCGT----------TTA-GACGCG--------TGC-CGTAT-----CA-------------------------------------------------------------------------------------------------------------------------------------------------------------------------------------------------------------GATATA-A-TAACGACG---T-A------ATG---ACAC--G-------------------------------------------------------------TTA---CGTTAATGATCCTTCCGCAGGTTCCCCTACGGAAACCTTGTTACGACTTTTACTTCC [354]

Y.cagnagellus2 -------------------------------------------------------------------------------------------------------------------------------------------------------------------------------------------------------------------------------------------------------------------------------------------------------------------------------------------------------------------------------------------------------------------------------------------------------------------------------------------------------------------------------------------------------------------------------------------------------------------------------------------------------------------------------------------------------------------------------------------------------------------------------------------------------------------------------------------------------------------------------------------------------------------------------------------------------------------------------------------------------------------------------------------------------------------------------------------------------------------------------------------------------------------------------------------------------------------------------------------------------------------------------------------------------------------------------------------------------------------------------------------------------------------------------------------------------------------------------------------------------------------------------------------------------------------------------------------------------------------------------------------------------GCTGCGTTCTTCATCGATGCGCGAGCCAAGTGATCCACCGTCCAGGGTGATGATTTGC--CGTTTGGCGTTGATCGAAATTGAGAG--AAAAAAATTCTACATACACCTAGGTGATAT--GTGTGCACGTGTGTGCGTG----TG-------------------------------------------------------------------------------------------------------------------------TAACGACAAACGAGCGCTCTC--ACGC---CGTCTCTCACG---------ACG-C--GAGGCGC-TCGC-CATCACCGCATAATA-------------------------------------------T--A-TCG-GCACGTGCGACTCCGGTCGCGGATTTTG-AACCCATCGATGTCGT----------TTA-GACGCG--------TGC-CGTAT-----CA-------------------------------------------------------------------------------------------------------------------------------------------------------------------------------------------------------------GATATA-A-TAACGACG---T-A------ATG---ACAC--G-------------------------------------------------------------TTA---CGTTAATGATCCTTCCGCAGGTTCCCCTACGGAAACCTTGTTACGACTTTTACTTCC [354]

Y.cagnagellus3 -------------------------------------------------------------------------------------------------------------------------------------------------------------------------------------------------------------------------------------------------------------------------------------------------------------------------------------------------------------------------------------------------------------------------------------------------------------------------------------------------------------------------------------------------------------------------------------------------------------------------------------------------------------------------------------------------------------------------------------------------------------------------------------------------------------------------------------------------------------------------------------------------------------------------------------------------------------------------------------------------------------------------------------------------------------------------------------------------------------------------------------------------------------------------------------------------------------------------------------------------------------------------------------------------------------------------------------------------------------------------------------------------------------------------------------------------------------------------------------------------------------------------------------------------------------------------------------------------------------------------------------------------------GCTGCGTTCTTCATCGATGCGCGAGCCAAGTGATCCACCGTCCAGGGTGATGATTTGC--CGTTTGGCGTTGATCGAAATTGAAAG--AAAAAAATTCTACATACACCTAGGTGATAT--GTGTGCACGTGTGTGCGTG----TG-------------------------------------------------------------------------------------------------------------------------TAACGACAAACGAGCGCTCTC--ACGC---CGTCTCTCACG---------ACG-C--GAGGCGC-TCGC-CATCACCGCATAATA-------------------------------------------T--A-TCG-GCACGTGCGACTCCGGGCGCGGATTTTG-AACCCATCGATGTCGT----------TTA-GACGCG--------TGC-CGTAT-----CA-------------------------------------------------------------------------------------------------------------------------------------------------------------------------------------------------------------GATATA-A-TAACGACG---T-A------ATG---ACAC--G-------------------------------------------------------------TTA---CGTTAATGATCCTTCCGCAGGTTCCCCTACGGAAACCTTGTTACGACTTTTACTTCC [354]

Y.padellus1 -------------------------------------------------------------------------------------------------------------------------------------------------------------------------------------------------------------------------------------------------------------------------------------------------------------------------------------------------------------------------------------------------------------------------------------------------------------------------------------------------------------------------------------------------------------------------------------------------------------------------------------------------------------------------------------------------------------------------------------------------------------------------------------------------------------------------------------------------------------------------------------------------------------------------------------------------------------------------------------------------------------------------------------------------------------------------------------------------------------------------------------------------------------------------------------------------------------------------------------------------------------------------------------------------------------------------------------------------------------------------------------------------------------------------------------------------------------------------------------------------------------------------------------------------------------------------------------------------------------------------------------------------------GCTGCGTTCTTCATCGATGCGCGAGCCAAGTGATCCACCGTCCAGGGTGATGATTTGC--CGTTTGGCGTTGATCGAAATTGAAAG--AAAAAAATTCTACATACACCTAGGTGATAT--GCGTGCACGTGTGTGCGTG----TG-------------------------------------------------------------------------------------------------------------------------TAACGACAAACGAGCGCTCTC--ACGC---CGTCTCTCACG---------ACG-C--GAGGCGC-TCGC-CATCACCGCATAATATT-----------------------------------------T--A-TCG-GCACGTGCGACTCCGGGCGCGGATTTTG-AACCCATCGATGTCGT----------TTA-GACGCG--------TGC-CGTAT-----CA-------------------------------------------------------------------------------------------------------------------------------------------------------------------------------------------------------------GATATA-A-TAACGACG---T-A------ATG---ACAC--G-------------------------------------------------------------TTA---CGTTAATGATCCTTCCGCAGGTTCCCCTACGGAAACCTTGTTACGACTTTTACTTCC [356]

Y.padellus2 -------------------------------------------------------------------------------------------------------------------------------------------------------------------------------------------------------------------------------------------------------------------------------------------------------------------------------------------------------------------------------------------------------------------------------------------------------------------------------------------------------------------------------------------------------------------------------------------------------------------------------------------------------------------------------------------------------------------------------------------------------------------------------------------------------------------------------------------------------------------------------------------------------------------------------------------------------------------------------------------------------------------------------------------------------------------------------------------------------------------------------------------------------------------------------------------------------------------------------------------------------------------------------------------------------------------------------------------------------------------------------------------------------------------------------------------------------------------------------------------------------------------------------------------------------------------------------------------------------------------------------------------------------GCTGCGTTCTTCATCGATGCGCGAGCCAAGTGATCCACCGTCCAGGGTGATGATTTGC--CGTTTGGCGTTGATCGAAATTGAAAG--AAAAAAATTCTACATACACCTAGGTGATAT--GTGTGCACGTGTGTGCGTG----TG-------------------------------------------------------------------------------------------------------------------------TAACGACAAACGAGCGCTCTC--ACGC---CGTCTCTCACG---------ACG-C--GAGGCGC-TCGC-CATCACCGCATAATA-------------------------------------------T--A-TCG-GCACGTGCGACTCCGGGCGCGGATTTTG-AACCCATCGATGTCGT----------TTA-GACGCG--------TGC-CGTAT-----CA-------------------------------------------------------------------------------------------------------------------------------------------------------------------------------------------------------------GATATA-A-TAACGACG---T-A------ATG---ACAC--G-------------------------------------------------------------TTA---CGTTAATGATCCTTCCGCAGGTTCCC-TACGGAAACCTTGTTACGACTTTTACTTCC [353]

Y.padellus3 -------------------------------------------------------------------------------------------------------------------------------------------------------------------------------------------------------------------------------------------------------------------------------------------------------------------------------------------------------------------------------------------------------------------------------------------------------------------------------------------------------------------------------------------------------------------------------------------------------------------------------------------------------------------------------------------------------------------------------------------------------------------------------------------------------------------------------------------------------------------------------------------------------------------------------------------------------------------------------------------------------------------------------------------------------------------------------------------------------------------------------------------------------------------------------------------------------------------------------------------------------------------------------------------------------------------------------------------------------------------------------------------------------------------------------------------------------------------------------------------------------------------------------------------------------------------------------------------------------------------------------------------------------GCTGCGTTCTTCATCGATGCGCGAGCCAAGTGATCCACCGTCCAGGGTGATGATTTGC--CGTTTGGCGTTGATCGAAATTGAAAG--AAAAAAATTCTACATACACCTAGGTGATGT--GTGTGCACGTGTGTGCGTG----TG-------------------------------------------------------------------------------------------------------------------------TAACGACAAACGAGCGCTCTC--ACGC---CGTCTCTCACG---------ACG-C--GAGGCGC-TCGC-CATCACCGCATAATA-------------------------------------------T--A-TCG-GCACGTGCGACTCCGGGCGCGCATTTTG-AACCCATCGATGTCGT----------TTA-GACGCG--------TGC-CGTAT-----CA-------------------------------------------------------------------------------------------------------------------------------------------------------------------------------------------------------------GATATA-A-TAACGACG---T-A------ATG---ACAC--G-------------------------------------------------------------TTA---CGTTAATGATCCTTCCGCAGGTTCCCCTACGGAAACCTTGTTACGACTTTTACTTCC [354]

Y.padellus4 -------------------------------------------------------------------------------------------------------------------------------------------------------------------------------------------------------------------------------------------------------------------------------------------------------------------------------------------------------------------------------------------------------------------------------------------------------------------------------------------------------------------------------------------------------------------------------------------------------------------------------------------------------------------------------------------------------------------------------------------------------------------------------------------------------------------------------------------------------------------------------------------------------------------------------------------------------------------------------------------------------------------------------------------------------------------------------------------------------------------------------------------------------------------------------------------------------------------------------------------------------------------------------------------------------------------------------------------------------------------------------------------------------------------------------------------------------------------------------------------------------------------------------------------------------------------------------------------------------------------------------------------------------GCTGCGTTCTTCATCGATGCGCGAGCCAAGTGATCCACCGTCCAGGGTGATGATTTGC--CGTTTGGCGTTGATCGAAATTGAAAG--AAAAAAATTCTACATACACCTAGGTGATAT--GTGTGCACGTGTGTGCGTG----TG-------------------------------------------------------------------------------------------------------------------------TAACGACAAACGAGCGCTCTC--ACGC---CGTCTCTCACG---------ACG-C--GAGGCGC-TCGC-CATCACCGCATAATA-------------------------------------------T--A-TCG-GCACGTGCGACTCCGGGCGCGGATTTTG-AACCCATCGATGTCGT----------TTA-GACGCG--------TGC-CGTAT-----CA-------------------------------------------------------------------------------------------------------------------------------------------------------------------------------------------------------------GATATA-A-TAACGACG---T-A------ATG---ACAC--G-------------------------------------------------------------TTA---CGTTAATGATCCTTCCGCAGGTTCCC-TACGGAAACCTTGTTACGACTTTTACTTCC [353]

Y.padellus5 -------------------------------------------------------------------------------------------------------------------------------------------------------------------------------------------------------------------------------------------------------------------------------------------------------------------------------------------------------------------------------------------------------------------------------------------------------------------------------------------------------------------------------------------------------------------------------------------------------------------------------------------------------------------------------------------------------------------------------------------------------------------------------------------------------------------------------------------------------------------------------------------------------------------------------------------------------------------------------------------------------------------------------------------------------------------------------------------------------------------------------------------------------------------------------------------------------------------------------------------------------------------------------------------------------------------------------------------------------------------------------------------------------------------------------------------------------------------------------------------------------------------------------------------------------------------------------------------------------------------------------------------------------GCTGCGTTCTTCATCGATGCGCGAGCCAAGTGATCCACCGTCCAGGGTGATGATTTGC--CGTTTGGCGTTGATCGAAATTGAAAG--AAAAAAATTCTACATACACCTAGGTGATAT--GTGTGCACGTGTGTGCGTG----TG-------------------------------------------------------------------------------------------------------------------------TAACGACAAACGAGCGCTCTC--ACGC---CGTCTCTCACG---------ACG-C--GAGGCGC-TCGC-CATCACCGCATAATA-------------------------------------------T--A-TCG-GCACGTGCGACTCCGGGCGCGGATTTTG-AACCCATCGATGTCGT----------TTA-GACGCG--------TGC-CGTAT-----CA-------------------------------------------------------------------------------------------------------------------------------------------------------------------------------------------------------------GATATA-A-TAACGACG---T-A------ATG---ACAC--G-------------------------------------------------------------TTA---CGTTAATGATCCTTCCGCAGGTTCCC-TACGGAAACCTTGTTACGACTTTTACTTCC [353]

Y.padellus6 -------------------------------------------------------------------------------------------------------------------------------------------------------------------------------------------------------------------------------------------------------------------------------------------------------------------------------------------------------------------------------------------------------------------------------------------------------------------------------------------------------------------------------------------------------------------------------------------------------------------------------------------------------------------------------------------------------------------------------------------------------------------------------------------------------------------------------------------------------------------------------------------------------------------------------------------------------------------------------------------------------------------------------------------------------------------------------------------------------------------------------------------------------------------------------------------------------------------------------------------------------------------------------------------------------------------------------------------------------------------------------------------------------------------------------------------------------------------------------------------------------------------------------------------------------------------------------------------------------------------------------------------------------GCTGCGTTCTTCATCGATGCGCGAGCCAAGTGATCCACCGTCCAGGGTGATGATTTGC--CGTTTGGCGTTGATCGAAATTGAAAG--AAAAAAATTCTACATACACCTAGGTGATAT--GTGTGCACGTGTGTGCGTG----TG-------------------------------------------------------------------------------------------------------------------------TAACGACAAACGAGCGCTCTC--ACGC---CGTCTCTCACG---------ACG-C--GAGGCGC-TCGC-CATCACCGCATAATA-------------------------------------------T--A-TCG-GCACGTGCGACTCCGGGCGCGGATTTTG-AACCCACCGATGTCGT----------TTA-GACGCG--------TGC-CGTAT-----CA-------------------------------------------------------------------------------------------------------------------------------------------------------------------------------------------------------------GATATA-A-TAACGACG---T-A------ATG---ACAC--G-------------------------------------------------------------TTA---CGTTAATGATCCTTCCGCAGGTTCCC-TACGGAAACCTTGTTACGACTTTTACTTCC [353]

Y.padellus7 -------------------------------------------------------------------------------------------------------------------------------------------------------------------------------------------------------------------------------------------------------------------------------------------------------------------------------------------------------------------------------------------------------------------------------------------------------------------------------------------------------------------------------------------------------------------------------------------------------------------------------------------------------------------------------------------------------------------------------------------------------------------------------------------------------------------------------------------------------------------------------------------------------------------------------------------------------------------------------------------------------------------------------------------------------------------------------------------------------------------------------------------------------------------------------------------------------------------------------------------------------------------------------------------------------------------------------------------------------------------------------------------------------------------------------------------------------------------------------------------------------------------------------------------------------------------------------------------------------------------------------------------------------GCTGCGTTCTTCATCGATGCGCGAGCCAAGTGATCCACCGTCCAGGGTGATGATTTGC--CGTTTGGCGTTGATCGAAATTGAAAG--AAAAAA-TTCTACATACACCTAGGTGATAT--GTGTGCACGTGTGTGCGTGCGTGTG-------------------------------------------------------------------------------------------------------------------------TAACGACAAACGAGCGCTCTC--ACGC---CGTCTCCCACG---------ACG-C--GAGGCGC-TCGC-CATCACCGCATAATA-------------------------------------------T--A-TCG-GCACGTGCGACTCCGGGCGCGGATTTTG-AACCCATCGATGTCGT----------TTA-GACGCG--------TGC-CGTAT-----CA-------------------------------------------------------------------------------------------------------------------------------------------------------------------------------------------------------------GATATA-A-TAACGACG---T-A------ATG---ACAC--G-------------------------------------------------------------TTA---CGTTAATGATCCTTCCGCAGGTTCCC-TACGGAAACCTTGTTACGACTTTTACTTCC [356]

Y.padellus8 -------------------------------------------------------------------------------------------------------------------------------------------------------------------------------------------------------------------------------------------------------------------------------------------------------------------------------------------------------------------------------------------------------------------------------------------------------------------------------------------------------------------------------------------------------------------------------------------------------------------------------------------------------------------------------------------------------------------------------------------------------------------------------------------------------------------------------------------------------------------------------------------------------------------------------------------------------------------------------------------------------------------------------------------------------------------------------------------------------------------------------------------------------------------------------------------------------------------------------------------------------------------------------------------------------------------------------------------------------------------------------------------------------------------------------------------------------------------------------------------------------------------------------------------------------------------------------------------------------------------------------------------------------GCTGCGTTCTTCATCGATGCGCGAGCCAAGTGATCCACCGTCCAGGGTGATGATTTGC--CGTTTGGCGTTGATCGAAATTGAAAG--AAAAAAATTCTACATACACCTAGGTGATAT--GTGTGCACGTGTGTGCGTG----TG-------------------------------------------------------------------------------------------------------------------------TAACGACAAACGAGCGCTCTC--ACGC---CGTCTCTCACG---------ACG-C--GAGGCGC-TCGC-CATCACCGCATAATA-------------------------------------------T--A-TCG-GCACGTGCGACTCCGGGCGCGGATTTTG-AACCCATCGATGTCGT----------TTA-GACGCG--------TGC-CGTAT-----CA-------------------------------------------------------------------------------------------------------------------------------------------------------------------------------------------------------------GATATA-A-TAACGACG---T-A------ATG---ACACACG-------------------------------------------------------------TTA---CGTTAATGATCCTCCCGCAGGTTCCC-TACGGAAACCTTGTTACGACTTTTACTTCC [355]

Y.padellus9 -------------------------------------------------------------------------------------------------------------------------------------------------------------------------------------------------------------------------------------------------------------------------------------------------------------------------------------------------------------------------------------------------------------------------------------------------------------------------------------------------------------------------------------------------------------------------------------------------------------------------------------------------------------------------------------------------------------------------------------------------------------------------------------------------------------------------------------------------------------------------------------------------------------------------------------------------------------------------------------------------------------------------------------------------------------------------------------------------------------------------------------------------------------------------------------------------------------------------------------------------------------------------------------------------------------------------------------------------------------------------------------------------------------------------------------------------------------------------------------------------------------------------------------------------------------------------------------------------------------------------------------------------------GCTGCGTTCTTCATCGATGCGCGAGCCAAGTGATCCACCGTCCAGGGTGATGATTTGC--CGTTTGGCGTTGATCGAAATTGAAAG--AAAAAAATTCTACATACACCTAGGTGATAT--GTGTGCACGTGTGTGCGTG----TG-------------------------------------------------------------------------------------------------------------------------TAACGACAAACGAGCGCTCTC--ACGC---CGTCTCTCACG---------ACG-C--GAGGCGC-TCGC-CATCACCGCATAATA-------------------------------------------T--A-TCG-GCACGTGCGACTCCGGGCGCGGATTTTG-AACCCATCGATGTCGT----------TTA-GACGCG--------TGC-CGTAT-----CATCA----------------------------------------------------------------------------------------------------------------------------------------------------------------------------------------------------------GATATA-A-TAACGACG---T-A------ATG---ACAC--G-------------------------------------------------------------TTA---CGTTAATGATCCTTCCGCAGGTTCCCCTACGGAAACCTTGTTACGACTTTTACTTCG [357]

Y.padellus10 -------------------------------------------------------------------------------------------------------------------------------------------------------------------------------------------------------------------------------------------------------------------------------------------------------------------------------------------------------------------------------------------------------------------------------------------------------------------------------------------------------------------------------------------------------------------------------------------------------------------------------------------------------------------------------------------------------------------------------------------------------------------------------------------------------------------------------------------------------------------------------------------------------------------------------------------------------------------------------------------------------------------------------------------------------------------------------------------------------------------------------------------------------------------------------------------------------------------------------------------------------------------------------------------------------------------------------------------------------------------------------------------------------------------------------------------------------------------------------------------------------------------------------------------------------------------------------------------------------------------------------------------------------GCTGCGTTCTTCATCGATGCGCGAGCCAAGTGATCCACCGTCCAGGGTGATGATTTGC--CGTTTGGCGTTGATCGAAATTGAAAG--AAAAAAATTCTACATACACCTAGGTGATAT--GTGTGCACGTGTGTGCGTG----TG-------------------------------------------------------------------------------------------------------------------------TAACGACAAACGAGCGCTCTT--ACGC---CGTCTCTCGCG---------ACG-C--GAGGCGC-TCGC-CATCACCGCATAATA-------------------------------------------T--A-TCG-GCACGTGCGACTCCGGGCGCGGATTTTG-AACCCATCGATGTCGT----------TTA-GACGCG--------TGC-CGTAT-----CA-------------------------------------------------------------------------------------------------------------------------------------------------------------------------------------------------------------GATATA-A-TAACGACG---T-A------ATG---ACACACG-------------------------------------------------------------TTA---CGTTAATGATCCTTCCGCAGGTTCCC-TACGGAAACCTTGTTACGACTTTTACTTCC [355]

Y.padellus11 -------------------------------------------------------------------------------------------------------------------------------------------------------------------------------------------------------------------------------------------------------------------------------------------------------------------------------------------------------------------------------------------------------------------------------------------------------------------------------------------------------------------------------------------------------------------------------------------------------------------------------------------------------------------------------------------------------------------------------------------------------------------------------------------------------------------------------------------------------------------------------------------------------------------------------------------------------------------------------------------------------------------------------------------------------------------------------------------------------------------------------------------------------------------------------------------------------------------------------------------------------------------------------------------------------------------------------------------------------------------------------------------------------------------------------------------------------------------------------------------------------------------------------------------------------------------------------------------------------------------------------------------------------GCTGCGTTCTTCATCGATGCGCGAGCCAAGTGATCCACCGTCCAGGGTGATGATTTGC--CGTTTGGCGTTGATCGAAATTGAAAG--AAAAAAATTCTACATACACCTAGGTGATAT--GTGTGCACGTGTGTGCGTG----TG-------------------------------------------------------------------------------------------------------------------------TAACGACAAACGAGCGCTCTC--ACGC---CGTCTCTCACG---------ACG-C--GAGGCGC-TCGC-CATCACCGCATAATA-------------------------------------------T--A-TCG-GCACGTGCGACTCCGGGCGCGGATTTTG-AACCCATCGATGTCGT----------TTA-GACGCG--------TGC-CGTAT-----CA-------------------------------------------------------------------------------------------------------------------------------------------------------------------------------------------------------------GATATA-A-TAACGACG---T-A------ATG---ACAC--G-------------------------------------------------------------TTA---CGTTAATGATCCTTCCGCAGGTTCCCCTACGGAAACCTTGTTACGACTTTTACTTCC [354]

Y.padellus12 -------------------------------------------------------------------------------------------------------------------------------------------------------------------------------------------------------------------------------------------------------------------------------------------------------------------------------------------------------------------------------------------------------------------------------------------------------------------------------------------------------------------------------------------------------------------------------------------------------------------------------------------------------------------------------------------------------------------------------------------------------------------------------------------------------------------------------------------------------------------------------------------------------------------------------------------------------------------------------------------------------------------------------------------------------------------------------------------------------------------------------------------------------------------------------------------------------------------------------------------------------------------------------------------------------------------------------------------------------------------------------------------------------------------------------------------------------------------------------------------------------------------------------------------------------------------------------------------------------------------------------------------------------GCTGCGTTCTTCATCGATGCGCGAGCCAAGTGATCCACCGTCCAGGGTGATGATTTGC--CGTTTGGCGTTGATCGAAATTGAAAG--AAAAAAATTCTACATACACCTAGGTGATAT--GTGTGCACGTGTGTGCGTG----TG-------------------------------------------------------------------------------------------------------------------------TAACGACAAACGAGCGCTCTC--ACGC---CGTCTCTCGCG---------ACG-C--GAGGCGC-TCGC-CATCACCGCATAATA-------------------------------------------T--A-TCG-GCACGTGCGACTCCGGGCGCGGATTTTG-AACCCATCGATGTCGT----------TTA-GACGCG--------TGC-CGTAT-----CA-------------------------------------------------------------------------------------------------------------------------------------------------------------------------------------------------------------GATATA-A-TAACGACG---T-A------ATG---ACAC--G-------------------------------------------------------------TTA---CGTTAATGATCCTTCCGCAGGTTCCC-TACGGAAACCTTGTTACGACTTTTACTTCC [353]

Y.padellus13 -------------------------------------------------------------------------------------------------------------------------------------------------------------------------------------------------------------------------------------------------------------------------------------------------------------------------------------------------------------------------------------------------------------------------------------------------------------------------------------------------------------------------------------------------------------------------------------------------------------------------------------------------------------------------------------------------------------------------------------------------------------------------------------------------------------------------------------------------------------------------------------------------------------------------------------------------------------------------------------------------------------------------------------------------------------------------------------------------------------------------------------------------------------------------------------------------------------------------------------------------------------------------------------------------------------------------------------------------------------------------------------------------------------------------------------------------------------------------------------------------------------------------------------------------------------------------------------------------------------------------------------------------------GCTGCGTTCTTCATCGATGCGCGAGCCAAGTGATCCACCGTCCAGGGTGATGATTTGC--CGTTTGGCGTTGATCGAAATTGAAAG-AAAAAAAATTCTACATACACCTAGGTGATAT--GTGTGCACGTGTGTGCGTG----TG-------------------------------------------------------------------------------------------------------------------------TAACGACAAACGAGCGCTCTC--ACGC---CGTCTCTCACG---------ACG-C--GAGGCGC-TCGC-CATCACCGCATAATA-------------------------------------------T--A-TCG-GCACGTGCGACTCCGGGCGCGGATTTTG-AACCCATCGATGTCGT----------TTA-GACGCG--------TGC-CGTAT-----CA-------------------------------------------------------------------------------------------------------------------------------------------------------------------------------------------------------------GATATA-A-TAACGACG---T-A------ATG---ACAC--G-------------------------------------------------------------TTA---CGTTAATGATCCTTCCGCAGGTTCCCCTACGGAAACCTTGTTACGACTTTTACTTCC [355]

Y._cagnagellus[4] CGCCTGTTTATCAAAAACATGTCTTTTTGATTAATAATTTAAAGTCTGGTCTGCCCA-CTGATT-A-AAATAATTAAAGGGCTGCAGTATTTTGACTGTACAAAGGTAGCATAATAATTAGTCTTTTAATTGATGACTTGTATGAATGATTGGATAAAATATAAGCTGTCTCTTAATAAATTTATAGAATTTAATTTTTTATTTAAAAAGTTAAAATAATTTTAAAAGACGAGAAGACCCTATAGAGTTTAATAA-TTTTATAAATTAAAATTATATTTATAAA-TTTAATTAAAAATTATAT-AATTATTTTATTGGGGTGATAAAAAAATTAATAAAACTTTTTTTAAATATTA---------ACAAAAATAATTGAATATTTGATCCAATTTT-ATTTTGATTATAAGATTAAATTACCTTAGGGATAACAGCGTAATTTTTTTTTTTAGTTCTTATAAAAAATAAAGTTTGCGACCTCGATGTTGGATTAAGATAAGATTTAAATGCAAAAGTTTAAAATTTTGATCTGTTCGATCATTAAAATCTTA-CATGATCTGAGTTCAAACCGGAGATACCTCGACGTTATTCAGATTATCCTGATGCTTATATTTGTTGAAATATTATTTCTTCTTTAGGATCCTATATTTCATTTTTAGCAATTATAATAATATTAATTATTATTTGAGAATCATTTATTTCTCAACGAATAATTTTATTTTCATTAAACATACCTTCTTCTATTGAATGACTCCAAAAATTCCCACCATCAGAACATTCATATAATGAACTTCCTATTTTAAGAAACTTCTAATAGTGCAGACTATATGTAATGGATTTAAACCCCATTTATAAAGGTTAATCCTTTTTTTAGAAATGGCAACATGAAATAATTTAAATTTACAAAATGGAGCATCTCCTTTAATAGAACAAATCATTTTTTTTCATGATCATACATTAATTATTTTAATTATAATTACAATCTTAGTAGGATATTTAATAATTAATTTATTTTTTAATAAATATATTAATCGATTTTTATTAGAAGGACAAATAATTGAATTAATTTGAACAATTTTACCAGCAATTACTTTAATTTTTATTGCTCTTCCATCTCTTCGTTTATTATATTTATTAGATGAACTTAATAATCCTTTAATTACATTAAAATCTATTGGTCATCAATGATATTGAAGTTATGAATATTCAGATTTTAATAATATTCAATTTGATTCTTATATAATCCCAAGAAAAGAAATAAAATTTAATGAATTTCGATTATTAGATGTAGATAATCGTATTATTCTTCCTATAAATAACCAAATTCGTATTATAGTAACAGCAACAGATGTAATTCATTCTTGAACAGTTCCATCTTTAGGAGTTAAAATTGATGCTAATCCAGGACGTTTAAATCAAACAAATTTTTTCATTAATCGTCCTGGATTATTTTATGGACAATGTTCTGAAATTTGTGGAGCAAACCATAGATTTATACCTATTGTAATTGAAAGAATTTCAATTAACAATTTTATTAAATGAATTAATAATTACTCTTCATTAGATGACTGAAAGCAAGTAATGGTCTCGCTGCGTTCTTCATCGATGCGCGAGCCAAGTGATCCACCGTCCAGGGTGATGATTTGC--CGTTTGGCGTTGATCGAAATTGAAAG--AAAAAAATTCTACATACACCTAGGTGATAT--GTGTGCACGTGTGTGCGTG----TG-------------------------------------------------------------------------------------------------------------------------TAACGACAAACGAGCGCTCTC--ACGC---CGTCTCTCACG---------ACG-C--GAGGCGC-TCGC-CATCACCGCATAATA-------------------------------------------T--A-TCG-GCACGTGCGACTCCGGGCGCGGATTTTG-AACCCATCGATGTCGT----------TTA-GACGCG--------TGC-CGTAT-----CA-------------------------------------------------------------------------------------------------------------------------------------------------------------------------------------------------------------GATATA-A-TAACGACG---T-A------ATG---ACAC--G-------------------------------------------------------------TTA---CGTTAATGATCCTTCCGCAGGTTCCC-TACGGAAACCTTGTTACGACTTTTACTTCC [1927]

Y._padellus[14] CGCCTGTTTATCAAAAACATGTCTTTTTGATTAATAATTTAAAGTCTGGTCTGCCCA-CTGATT-A-AAATAATTAAAGGGCTGCAGTATTTTGACTGTACAAAGGTAGCATAATAATTAGTCTTTTAATTGATGACTTGTATGAATGATTGGATAAAGTATAATCTGTCTCTTAATAAATTTATAGAATTTAATTTTTTATTTAAAAAGTTAAAATAATTTTAAAAGACGAGAAGACCCTATAGAGTTTAATAA-TTTTATAAATTAAAATTATATTTATAAA-TTTAATTAAAAATTATAT-AATTATTTTATTGGGGTGATAAAAAAATTAATAAAACTTTTTTTAAATATTA---------ACAAAAATAATTGAATATTTGATCCAATTTT-ATTTTGATTATAAGATTAAATTACCTTAGGGATAACAGCGTAATTTTTTTTTTTAGTTCTTATAAAAAAGAAAGTTTGCGACCTCGATGTTGGATTAAGATAAAATTTAAATGCAAAAGTTTAAAATTTTGATCTGTTCGATCATTAAAATCTTA-CATGATCTGAGTTCAAACCGGAGATACCTCGACGTTATTCAGATTATCCTGATGCTTATATTTGTTGAAATATTATTTCTTCTTTAGGATCCTATATTTCATTTTTAGCAATTATAATAATATTAATTATTATTTGAGAATCATTTATTTCTCAACGAATAATTTTATTTTCATTAAACATACCTTCTTCTATTGAATGACTCCAAAAATTCCCACCATCAGAACATTCATATAATGAACTTCCCATTTTAAGAAACTTCTAATATGGCAGACTATATGTAATGGATTTAAACCCCATTTATAAAGGTTAATCCTTTTTTTAGAAATGGCAACATGAAATAATTTAAATTTACAAAATGGAGCATCTCCTTTAATAGAACAAATCATTTTTTTTCACGATCATACATTAATTATTTTAATTATAATTACAATCTTAGTAGGATATTTAATAATTAATTTATTTTTTAATAAATATATTAATCGATTTTTATTAGAAGGACAAATAATTGAATTAATTTGAACAATTTTACCAGCAATTACTTTAATTTTTATTGCTCTTCCATCTCTTCGTTTATTATATTTATTAGATGAACTTAATAATCCTTTAATTACTTTAAAATCTATTGGTCATCAATGATATTGAAGTTATGAATATTCAGATTTTAATAATATTCAATTTGATTCTTATATAATTCCAAGAAAAGAAATAAAATTTAATGAATTTCGATTATTAGATGTAGATAATCGTATTATTCTTCCCATAAATAACCAAATTCGTATTATAGTAACAGCAACAGATGTAATTCATTCTTGAACAGTTCCATCTTTAGGGGTTAAAATTGATGCTAATCCAGGACGTTTAAATCAAACAAATTTTTTCATTAATCGTCCTGGATTATTTTATGGACAATGTTCTGAAATTTGTGGAGCAAACCATAGATTTATACCTATTGTAATTGAAAGAATTTCAATTAACAATTTTATTAAATGAATTAATAATTACTCTTCATTAGATGACTGAAAGCAAGTAATGGTCTCGCTGCGTTCTTCATCGATGCGCGAGCCAAGTGATCCACCGTCCAGGGTGATGATTTGC--CGTTTGGCGTTGATCGAAATTGAAAG--AAAAAAATTCTACATACACCTAGGTGATAT--GTGTGCACGTGTGTGCGTG----TG-------------------------------------------------------------------------------------------------------------------------TAACGACAAACGAGCGCTCTC--ACGC---CGTCTCTCACG---------ACG-C--GAGGCGC-TCGC-CATCACCGCATAATA-------------------------------------------T--A-TCG-GCACGTGCGACTCCGGGCGCGGATTTTG-AACCCATCGATGTCGT----------TTA-GACGCG--------TGC-CGTAT-----CA-------------------------------------------------------------------------------------------------------------------------------------------------------------------------------------------------------------GATATA-A-TAACGACG---T-A------ATG---ACAC--G-------------------------------------------------------------TTA---CGTTAATGATCCTTCCGCAGGTTCCC-TACGGAAACCTTGTTACGACTTTTACTTCC [1927]

Y._irrorellus CGCCTGTTTATCAAAAACATGTCTTTTTGATTAATAATTTAAAGTCTGGTCTGCCCA-CTGATT-A-AAATAATTAAAGGGCTGCAGTATTTTGACTGTACAAAGGTAGCATAATAATTAGTCTTTTAATTGATGACTTGTATGAATGATCGAATAAAATATAATCTGTCTCTTAATAAATTTATAGAATTTAATTTTTTATTTAAAAAGTTAAAATAATTTTAAAAGACGAGAAGACCCTATAGAGTTTAATAA-TTTTATAAATTAAAATTATATATATAAA-TTTAATTAAAAATTATAT-AATTATTTTATTGGGGTGATAAAAAAATTAATAAAACTTTTTTTAAATATTA---------ACAAAAATAATTGTATATTTGATCCAATTT--ATTTTGATTATAAGATTAAATTACCTTAGGGATAACAGCGTAATTTTTTTTTTTAGTTCTTATAAAAAGGAAAGCTTGCGACCTCGATGTTGGATTAAGATAAAATTTAAACGCAAAAGTTTAAAATTTTGATCTGTTCGATCATTAAAATCTTA-CATGATCTGAGTTCAAACCGGAGATACCTCGACGTTATTCAGATTATCCTGATGCTTATATTTGTTGAAATATTATTTCTTCTTTAGGTTCTTATATTTCATTTTTAGCAATTATAATAATATTAATTATTATTTGAGAATCATTTATCTCTCAACGAATAATTTTATTTTCATTAAATATACCTTCTTCTATTGAATGACTCCAAAAATTCCCACCATCAGAACATTCATATAATGAACTTCCTATTTTAAGAAACTTCTAATATGGCAGACTATATGTAATGGATTTAAACCCCATATATAAAGGTTAATCCTTTTTTTAGAAATGGCAACATGAAATAATTTAAATTTACAAAATGGAGCATCTCCTTTAATAGAACAAATCATTTTTTTTCATGATCATACATTAATTATTTTAATTATAATTACAATCTTAGTAGGATATTTAATAATCAATTTATTTTTTAATAAATATATTAATCGATTTTTATTAGAAGGACAAATAATTGAATTAATTTGAACAATTTTACCAGCAATTACTTTAATTTTTATTGCCCTTCCATCTCTTCGTTTATTATATTTATTAGATGAACTTAATAATCCTTTAATTACATTAAAATCTATTGGTCATCAATGATATTGAAGTTATGAATATTCAGATTTTAATGATATTCAATTTGATTCTTATATAATTCCAAGAAAAGAAATAAAATTTAATGAATTTCGATTATTAGATGTAGATAATCGTATTATTCTTCCTATAAATAATCAAATTCGTATTATAGTAACAGCAACAGATGTAATTCATTCTTGAACAATTCCATCTTTAGGAGTTAAAATTGATGCTAACCCAGGACGTTTAAATCAAACAAATTTTTTTATTAATCGTCCTGGATTATTTTATGGACAATGTTCTGAAATTTGTGGAGCAAACBATAGATTTATACCTATTGTAATTGAAAGAATTTCAATTAATAATTTTATTAAATGAATTAATAATTTCTCTTCATTAGATGACTGAAAGCAAGTAATGGTCTCGCTGCGTTCTTCATCGATGCGCGAGCCAAGTGATCCACCGTCCAGGGTAATGATTTGT-CCGTTTGGCGTTGATTGAAATTGAAAG-AAAAAAAA----ATATACATATAGGTAACATATGTATGTA-GTATGTG-----------------------------------------------------------------------------------------------------------------------------------TAACGACAAACGAGNGCTCTC--ACGC---CGTC----A-G---------ACG-C--GAGGCGC-TCGC-CATCACCGCATAGTTA---------------TTC-----------TATATATTTAACGTCCAC-CG-GCACGTGCGACTCCGGGTGCGGATTTTG-AACCCATCGATGTCGT----------TTAA-ACGCG--------TGC-CGT--------------------G--------CGAC------------GTCTC--TCT-------------T-------CGCC----G---------ATAAGGT-G------------------GCTC-------------------------GCGCGCGCCCA----C--ATC-CGCGTCGAGA--GAACGCT-----GAGGGA--T--ATC---AGATA-----TAACGATG---T-A------ATG-----AC--GATAGCGGCGGCAATAGTGGAT--GCTGCTGC---T-A-------CGCGCA-CA----C--GTTA---CGTTAATGATCCTTCCGCAGGTTCCCCTACGGAAACCTTGTTACGACTTTTACTTCC [2040]

Y._malinellus CGCCTGTTTATCAAAAACATGTCTTTTTGATTAATAATTTAAAGTCTGGTCTGCCCA-CTGATT-A-AAATAATTAAAGGGCTGCAGTATTTTGACTGTACAAAGGTAGCATAATAATTAGTCTTTTAATTGATGACTTGTATGAATGATTGGATAAAATATAAGCTGTCTCTTAATAAATTTATAGAATTTAATTTTTTATTTAAAAAGTTAAAATAATTTTAAAAGACGAGAAGACCCTATAGAGTTTAATAA-TTTTATAAATTAAAATTATATTTATAAA-TTTAATTAAAAATTATAT-AATTATTTTATTGGGGTGATAAAAAAATTAATAAAACTTTTTTTAAATATTA---------ACAAAAATAATTGAATATTTGATCCAATTTT-ATTTTGATTATAAGATTAAATTACCTTAGGGATAACAGVGTAATTTTTTTTTT-AGTTCTTATAAAAAATAAAGTTTGCGACCTCGATGTTGGATTAAGATAAAATTTAAATGCAAAAGTTTAAAATTTTGATCTGTTCGATCATTAAAATCTTA-CATGATCTGAGTTCAAACCGGAGATACCTCGACGTTATTCAGATTATCCTGATGCTTATATTTGTTGAAGTATTATTTCTTCTTTAGGATCCTATATTTCATTTTTAGCAATTATAATAATATTAATTATTATTTGAGAATCATTTATTTCTCAACGAATAATTTTATTTTCATTAAACATACCTTCTTCTATTGAATGACTCCAAAAATTCCCACCATCAGAACATTCATATAATGAACTTCCTATTTTAAGAAACTTCTAATATGGCAGACTATATGTAATGGATTTAAACCCCATTTATAAAGGTTAATCCTTTTTTTAGAAATGGCAACATGAAATAATTTAAATTTACAAAATGGAGCATCTCCTTTAATAGAACAAATCATTTTTTTTCATGATCATACATTAATTATTTTAATTATAATTACAATCTTAGTAGGATATTTAATAATTAATTTATTTTTTAATAAATATATTAATCGATTTTTATTAGAAGGACAAATAATTGAATTAATTTGAACAATTTTACCAGCAATTACTTTAATTTTTATTGCTCTTCCATCTCTTCGTTTATTATATTTATTAGATGAACTTAATAATCCTTTAATTACATTAAAATCTATTGGTCATCAATGATATTGAAGTTATGAATATTCAGATTTTAATAATATTCAATTTGATTCTTATATAATCCCAAGAAAAGAAATAAAATTTAATGAATTTCGATTATTAGATGTAGATAATCGTATTATTCTTCCTATAAATAACCAAATTCGTATTATAGTAACAGCAACAGATGTAATTCATTCTTGAACAGTTCCATCTTTAGGAGTTAAAATTGATGCTAATCCAGGACGTTTAAATCAAACAAATTTTTTCATTAATCGTCCTGGATTATTTTATGGACAATGTTCTGAAATTTGTGGAGCAAACCATAGATTTATACCTATTGTAATTGAAAGAATTTCAATTAACAATTTTATTAAATGAATTAATAATTACTCTTCATTAGATGACTGAAAGCAAGTAATGGTCTCGCTGCGTTCTTCATCGATGCGCGAGCCAAGTGATCCACCGTCCAGGGTGATGATTTGC--CGTTTGGCGTTGATCGAAATTAAAAGGAAAAAAAA-CGTACACTCA--TACGTGATAC--GCGTTT---TGTGCGCGTG----TG-------------------------------------------------------------------------------------------------------------------------TAACGACAAACGAGCGCTCTC--ACGC---CGTC----A-G---------ACG-C--GAGGCGC-TCGC-CATCACCGCATAATAG------------------------------------------TCCA-TCG-GCACGTGCGACTCCGGGCGCGGATTTTG-AACCCATCGATGTCGT----------TT-GGACGCG--------TGC-CGTAT--CATCA-------------------------------------------------------------------------------------------------------------------------------------------------------------------------------------------------------------GATATA-A-TAACGATG---C-A------ATG---ACAC--GATAGCGGCGGCAACGGTGGAT--GCTGCTGCTGCT-A-------CGCGCA-CA----C--GTTA---CGTTAATGATCCTTCCGCAGGTTCCCCTACGGAAACCTTGTTACGACTTTTACTTCC [1968]

Y._mahalebellus CGCCTGTTTATCAAAAACATGTCTTTTTGATTAATAATTTAAAGTCTGGTCTGCCCA-CTGATT-A-AATTAATTAAAGGGCTGCAGTATTTTGACTGTACAAAGGTAGCATAATAATTAGTCTTTTAATTGATGACTTGTATGAATGATTGGATAAAATATAATCTGTCTCTTAATAAATTTATAGAATTTAATTTTTTATTTAAAAAGTTAAAATAATTTTAAAAGACGAGAAGACCCTATAGAGTTTAATAA-TTTTATAAATTAAAATTATATTTATAAA-TTTAATTAAAAATTATAT-AATTATTTTATTGGGGTGATAAAAAAATTAATAAAACTTTTTTTAAATATTA---------ACAAAAATAATTGAATATTTGATCCAATTTT-ATTTTGATTATAAGATTAAATTACCTTAGGGATAACAGCGTAATTTTTTTTTTTAGTTCTTATAAAAAAGAAAGTTTGCGACCTCGATGTTGGATTAAGATAAAATTTAAATGCAAAAGTTTAAAACTTTGATCTGTTCGATCATTAAAATCTTA-CATGATCTGAGTTCAAACCGGAGATACCTCGACGTTATTCAGATTATCCTGATGCTTATATTTGTTGAAATATTATTTCTTCTTTAGGATCCTATATTTCATTTTTAGCAATTATAATAATATTAATTATTATTTGAGAATCATTTATTTCTCAACGAATAATTTTATTTTCATTAAACATACCTTCTTCTATTGAATGACTCCAAAAATTCCCACCATCAGAACATTCATATAATGAACTTCCTATTTTAAGAAACTTCTAATATGGCAGACTATATGTAATGGATTTAAACCCCATTTATAAAGGTTAATCCTTTTTTTAGAAATGGCAACATGAAATAATTTAAATTTACAAAATGGAGCATCTCCTTTAATAGAACAAATCATTTTTTTTCACGATCATACATTAATTATTTTAATTATAATTACAATCTTAGTAGGATATTTAATAATTAATTTATTTTTTAATAAATATATTAATCGATTTTTATTAGAAGGACAAATAATTGAATTAATTTGAACAATTTTACCAGCAATTACTTTAATTTTTATTGCTCTTCCATCTCTTCGTTTATTATATTTATTAGATGAACTTAATAATCCTTTAATTACTTTAAAATCTATTGGTCATCAATGATATTGAAGTTATGAATATTCAGATTTTAATAATATTCAATTTGATTCTTATATAATTCCATGAATAGATATTAAATTTAATGAATTTCGATTATTAGATGTAGATAATCGTATTATTCTTCCCATAAATAACCAAATTCGTATTATAGTAACAGCAACAGATGTAATTCATTCTTGAACAGTTCCATCTTTAGGGGTTAAAATTGATGCTATTCCAGGACGTTTAAATCAAACAAATTTTTTCATTAATCGTCCTGGATTATTTTATGGACAATGTTCTGAAATTTGTGGAGCAAACCATAGATTTATACCTATTGTAATTGAAAGAATTTCAATTAACAATTTTATTAAATGAATTAATAATTACTCTTCATTAGATGACTGAAAGCAAGTAATGGTCTCGCTGCGTTCTTCATCGATGCGCGAGCCAAGTGATCCACCGTCCAGGGTGATGATTTGT--TGTTTGGCTTTGATTGAAATTAAAAC--AAAATAA----ATACA-------------TT-GTGTGTA----------------TG-------------------------------------------------------------------------------------------------------------------------TAACGACAAACGAGCGCTCTC--ACGC---CGTC----A-G---------ACG-C--GAGGCGC-TCGC-CATCACCGCATAGTC-------------------------------------------TCCAC-CG-GCACGTGCGACTCCGGGCGCGGATTTTG-AACCCATCGATGTCGT----------TTA-GACGCG--------TGC-CGTATATCATCATCATCATCATC-------------------------------------------------------------------------------------------------------------------------------------------------------------------------------------------------AGATATT-A-TAACGATG---T-A------ATG---ACAC--GATAGC---------------T-----------GCTGCT-AC--GCGGGCA-CA----C--GTCA---CGTTAATGATCCTTCCGCAGGTTCCCCTACGGAAACCTTGTTACGACTTTTACTTCC [1939]

Y._rorrellus CGCCTGTTTATCAAAAACATGTCTTTTTGATTAATAATTTAAAGTCTGGTCTGCCCA-CTGATT-A-AAATAATTAAAGGGCTGCAGTATTTTGACTGTACAAAGGTAGCATAATAATTAGTCTTTTAATTGATGACTTGTATGAATGATTGGATAAAATATAAGCTGTCTCTTAATAAATTTATAGAATTTAATTTTTTATTTAAAAAGCTAAAATAATTTTAAAAGACGAGAAGACCCTATAGAGTTTAATAA-TTTTATAAATTAAAATTATATTTATAAA-TTTAATTAAAAATTATAT-AATTATTTTATTGGGGTGATAAAAAAATTAATAAAACTTTTTTTAAATATTA---------ACAAAAATAATTGAATATTTGATCCAATTTT-ATTTTGATTATAAGATTAAATTACCTTAGGGATAACAGCGTAATTTTTTTTTTTAGTTCTTATAAAAAATAAAGTTTGCGACCTCGATGTTGGATTAAGATAAAATTTAAATGCAAAAGTTTAAAATTTTGATCTGTTCGATCATTAAAATCTTA-CATGATCTGAGTTCAAACCGGAGATACCTCGACGTTATTCAGAATATCCTGATGCTTATATTTGTTGAAATATTATTTCTTCTTTAGGATCCTATATTTCATTTTTAGCAATTATAATAATATTAATTATTATTTGAGAATCATTTATTTCTCAACGAATAATTTTATTTTCATTAAACATACCTTCTTCTATTGAATGACTCCAAAAATTCCCACCATCAGAACATTCATATAATGAACTTCCTATTTTAAGAAACTTCTAATATGGCAGACTATATGTAATGGATTTAAACCCCATTTATAAAGGTTAATCCTTTTTTTAGAAATGGCAACATGAAATAATTTAAATTTACAAAATGGAGCATCTCCTTTAATAGAACAAATCATTTTTTTTCACGATCATACATTAATTATTTTAATTATAATTACAATCTTAGTAGGATATTTAATAATTAATTTATTTTTTAATAAATATATTAATCGATTTTTATTAGAAGGACTAATAATTGAATTAATTTGAACAATTTTACCAGCAATTACTTTAATTTTTATTGCTCTTCCATCTCTTCGTTTATTATATTTATTAGATGAACTTAATAATCCTTTAATTACTTTAAAATCTATTGGTCATCAATGATATTGAAGTTATGAATATTCAGATTTTAATAATATTCAATTTGATTCTTATATAATTCCAAGAAAAGRAATAAAATTTAATGAATTTCGATTATTAGATGTAGATAATCGTATTATTCTTCCCATAAATAACCAAATTCGTATTATAGTAACAGCAACAGATGTAATTCATTCTTGAACAGTTCCATCTTTAGGAGTTAAAATTGATGCTAATCCAGGACGTTTAAATCAAACAAATTTTTTTATTAATCGTCCTGGATTATTTTATGGACAATGTTCTGAAATTTGTGGAGCAAACCATAGATTTATACCTATTGTAATTGAAAGAATTTCAATTAACAATTTTATTAAATGAATTAATAATTACTCTTCATTAGATGACTGAAAGCAAGTAATGGTCTCGCTGCGTTCTTCATCGATGCGCGAGCCAAGTGATCCACCGTCCAGGGTGATGATTTGT--TGTTTGGCTTTGATTGAAATTAAAAC--AAAATAA----ACGCACA--TAGGT-ACATT-GTGTGTGCGTGTGTGTGTG----TG-------------------------------------------------------------------------------------------------------------------------TAACGACAAACGAGCGCTCTC--ACGC---CGTC----A-G---------ACG-C--GAGGCGC-TCGC-CATCACCGCATAGTC-------------------------------------------TCCAC-CG-GCACGTGCGACTCCGGGCGCGGATTTTG-AACCCATCGATGTCGT----------TTA-GACGCG--------TGC-CGTATATCATCA-------------------------------------------------------------------------------------------------------------------------------------------------------------------------------------------------------------GATAT-TA-TAACGATG---T-A------ATG----------ACACGTATAGC--------------------TGCTCGT-AC--GCGCGCA-CA----C--GTTA---CGTTAATGATCCTTCCGCAGGTTCCC-TACGGAAACCTTGTTACGACTTTTACTTCC [1949]

Y._gigas ------------------------------------------------------------------------------------------------------------------------------------------------------------------------------------------------------------------------------------------------------------------------------------------------------------------------------------------------------------------------------------------------------------------------------------------------------------------------------------------------------------------------------------------------------------------------------------------------ATACCTCGACGTTATTCAGATTATCCTGATGCTTATATTTGTTGAAATATTATTTCTTCTTTAGGATCCTATATTTCATTTTTAGCAATTATAATAATATTAATTATTATTTGAGAATCATTTATTTCTCAACGAATAATTTTATTTTCATTAAACATACCTTCTTCTATTGAATGACTCCAAAAATTCCCACCATCAGAACATTCATATAATGAACTTCCTATTTTAAGAAACTTCTAATATGGCAGACTATATGTAATGGATTTAAACCCCATTTATAAAGGTTAATCCTTTTTTTAGAAATGGCAACATGAAATAATTTAAATTTACAAAATGGAGCATCTCCTTTAATAGAACAAATCATTTTTTTTCATGATCATACATTAATTATTTTAATTATAATTACAATCTTAGTGGGATATTTAATAATTAATTTATTTTTTAATAAATATATTAATCGATTTTTATTAGAAGGACAAATAATTGAATTAATTTGAACAATTTTACCAGCAATTACTTTAATTTTTATTGCTCTTCCATCTCTTCGTTTATTATATTTATTAGATGAACTTAATAATCCTTTAATCACTTTAAAATCTATTGGTCATCAATGATATTGAAGTTATGAATATTCAGATTTTAATAATATTCAATTTGATTCTTATATAATTCCAAGAAAAGAAATAAAATTTAATGAATTTCGATTATTAGATGTAGATAATCGTATTATTCTTCCTATAAATAACCAAATTCGTATTATAGTAACAGCAACAGATGTAATTCATTCTTGAACAGTTCCATCTTTAGGAGTTAAAATTGATGCTAATCCAGGACGTTTAAATCAAACAAATTTTTTCATTAATCGTCCTGGATTATTTTATGGACAATGTTCTGAAATTTGTGGAGCAAACCATAGATTTATACCTATTGTAATTGAAAGAATTTCAATTAACAATTTTATTAAATGAATTAATAATTACTCTTCATTAGATGACTGAAAGCAAGTAATGGTCTCGCTGCGTTCTTCATCGATGCGCGAGCCAAGTGATCCACCGTTCAGGGTGATGATTTGT--TGTCTGGCTTTGATTGAAATTAAAAC--AAAATAA----ATACACA--TAGGT-ACATT-GTGTGTGCGTGTGTGTGTGTG--TG-------------------------------------------------------------------------------------------------------------------------TAACGACAAACGAGCGCTCTC--ACGC---CGTC----A-G---------ACG-C--GAGGCGC-TCGC-CATCACCGCATAGTC-------------------------------------------TCCAC-CG-GCACGTGCGACTCCGGGCGCGGATTTTG-AACCCATCGATGTCGT----------TTA-GACGCG--------TGC-CGTATATCATCA-------------------------------------------------------------------------------------------------------------------------------------------------------------------------------------------------------------GATAT-TA-TAACGATG---T-A------ATG----------ACACG-ATAGC--------------------TGCTGCT-AC--GCGCGCA-CA----C--GTTA---CGTTAATGATCCTTCCGCAGGTTCCCCTACGGAAACCTTGTTACGACTTTTACTTCC [1392]

Y._evonymellus CGCCTGTTTATCAAAAACATGTCTTTTTGATAAATAATTTAAAGTCTGGTCTGCCCA-CTGATT-A-AAATAATTAAAGGGCTGCAGTATTTTGACTGTACAAAGGTAGCATAATAATTAGTCTTTTAATTGATGACTTGTATGAATGATCGGATAAAATATAAGCTGTCTCTTAATAAATTTATAGAATTTAATTTTTTATTTAAAAAGTTAAAATAATTTTAAAAGACGAGAAGACCCTATAGAGTTTAATAA-TTTTATAAATTAAAATTATATTTATAAA-TTTAATTAAAAATTATAT-AATTATTTTATTGGGGTGATAGAAAAATTAATAAAACTTTTTTTAAATATTA---------ACAAAAATAATTGTATATTTGATCCAATTTT-ATTTTGATTATAAGATTAAATTACCTTAGGGATAACAGCGTAATTTTTTTTTTTAGTTCTTATAAAAAAAAAAGTTTGCGACCTCGATGTTGGATTAAGATAAAATTTAAATGCAAAAGTTTAAAATTTTGATCTGTTCGATCATTAAAATCTTA-CATGATCTGAGTTCAAACCGGAGATACCTCGACGTTATTCAGATTATCCTGATGCTTATATTTGTTGAAATATTATTTCTTCTTTAGGATCTTATATTTCATTTTTAGCAATTATAATAATATTAATTATTATTTGAGAATCATTTATTTATCAACGAATAATTTTATTTTCATTAAACATACCTTCTTCTATTGAATGACTCCAAAAATTCCCACCATCAGAACATTCATATAATGAACTTCCTATTTTAAGAAACTTCTAATATGGCAGACTATATGTAATGGATTTAAACCCCATTTATAAAGGTTAATCCTTTTTTTAGAAATGGCAACATGAAATAATTTAAATTTACAAAACGGAGCATCTCCTTTAATAGAACAAATCATTTTTTTTCATGATCATACATTAATTATTTTAATTATAATTACAATTTTAGTAGGATATTTAATAATTAATTTATTTTTTAATAAATATATTAATCGATTTTTATTAGAAGGGCAAATAATTGAATTAATTTGAACAATTTTACCAGCAATTACTTTAATTTTTATTGCTCTTCCATCTCTTCGTTTATTATATTTATTAGATGAACTTAATAATCCTTTAATTACATTAAAATCTATTGGTCATCAATGATATTGAAGTTATGAATATTCAGATTTTAATAATATTCAATTTGATTCTTATATAATTCCAAGAAAAGAAATAAAATTTAATGAATTTCGATTATTAGATGTAGATAATCGTATTATTCTTCCTATAAATAACCAAATTCGTATTATAGTAACAGCAACAGATGTAATTCATTCTTGAACAGTACCATCTTTAGGAGTTAAAATTGATGCTAATCCAGGACGTTTAAATCAAACAAATTTTTTTATTAATCGTCCTGGATTATTTTATGGACAATGTTCTGAGATTTGTGGAGCAAACCATAGATTTATACCTATTGTAATTGAAAGAATTTCAATTAATAATTTTATTAAATGAATTAATAATTACTCTTCATTAGATGACTGAAAGCAAGTAATGGTCTC----------------------------------------------------------------------------------------------------------------------------------------------------------------------------------------------------------------------------------------------------------------------------------------------------------------------------------------------------------------------------------------------------------------------------------------------------------------------------------------------------------------------------------------------------------------------------------------------------------------------------------------------------------------------------------------------------------------------------------------------------------------------------------------------------------------------------------------------------------------------------------------------- [1950]

Y._meguronis CGCCTGTTTATCAAAAACATGTCTTTTTGAA-TTTAATTTAAAGTCTGGTCTGCCCA-CTGATT----TATAATTGAAGGGCTGCAGTATATTGACTGTACAAAGGTAGCATAATCATTAGTCTTTTAATTGATGACTTGTATGAATGATCGGATAAAATATAAGCTGTCTCTAAATAAATTTATAGAATTTAATTTTTTATTTAAAAAGTTAAAATAATTTTAAAAGACGAGAAGACCCTATAGAGTTTAATAT-TTATTTAAATTAAAATTATATATTTAAA-TTTAATTAAAAATTAAAT-ATTTATTTTATTGGGGTGATAAAAAAATTAATTAAACTTTTTTTAATTTTTA---------ACATAGATAATTGAATATTTGATCCAATTTT---TTTGATTATAAGATTAAATTACCTTAGGGATAACAGCGTAATTTTTTTTTTTAGTTCATATAAAAAAAAAAGTTTGCGACCTCGATGTTGGATTAAGATAAAATTTAAATGCAAAAGTTTAAAATTTTGATCTGTTCGATCATTAATATCTTA-CATGATCTGAGTTCAAACCGGAGATACCTCGACGTTATTCAGATTACCCTGATGCTTATATTTGTTGAAATATTATTTCTTCATTAGGTTCTTATATTTCATTTCTAGCCATTATAATAATATTAATTATTATTTGAGAATCATTTATTTCTCAACGATTAATTTTATTTTCAATAAATATACCATCTTCTATTGAATGACTTCAAAAATTTCCTCCTTCTGAACATTCTTATAATGAACTTCCTATTTTAAGAAATTTCTAATATGGCAGATTATATGTAATGGATTTAAACCCCATTTATAAAGGTTTATCCTTTTTTTAGAAATGGCAACATGAAATAATTTAAATTTACAAAATGGAGCATCTCCTTTAATAGAACAAATCATTTTTTTTCATGATCATACATTAATTATTTTAATTATAATTACAATTCTAGTAGGATATTTAATAATTAATTTATTTTTTAATAAATATACTAATCGTTTTTTATTAGAAGGACAAATAATTGAATTAATTTGAACAATTTTACCTGCAATTACTTTAATTTTTATTGCTCTCCCATCTCTACGTTTATTATATTTATTAGATGAATTAAATAATCCATTAATTACATTAAAATCAATCGGACATCAATGATATTGAAGTTATGAATATTCAGATTTTAATAATATTCAATTTGATTCTTATATAATTCCTAGAAAAGAAATAAAAATTAATAATTTTCGATTATTAGATGTAGATAATCGTATTATTCTTCCCATAAATAATCAAATTCGTATTATAGTTACAGCAACAGATGTTATTCATTCTTGAACAATTCCATCTTTAGGAGTTAAAATTGATGCTAATCCAGGACGTTTAAACCAAACAAATTTTTTTATTAATCGTCCTGGTTTATTTTATGGTCAATGTTCTGAAATTTGTGGAGCAAATCATAGTTTTATACCTATTGTAATTGAAAGAATTTCAATTAACAATTTTATTAAATGAATTAATAATTATTCTTCATTAGATGACTGAAAGCAAGTAATGGTCTCGCTGCGTTCTTCATCGATGCGCGAGCCAAGTGATCCACCGTCCAGGGTAATGATTT----------------------------------------------------------------------------------------------G--GT-TCATCAC-CA-C----GTT--T-----T-GTAA-T---GC--------------GATATG---AGT--ATTC-GAAATCGA--TAA-A-ATTT-----AT----AA----TAACGACAAACGAGCGCTCTC--ACGCT---GTC----A------A----ACG-A--GAGGCGC-TCGC-CATCACCGC-------------AGA-G----TT-TT---GTTTGT---GT--TTAACGTCCACT-G-GCACGTGCGACTCCGGGCGCGGATTTTA-GACCCATCGATGTCGT----------TTA-GACGCG--------TGC-CGT--------------------G--------CGAC------------GTCTC--TCT-------------T-------CGCC----G---------AC--GGC-G--CG--------------CGCA--G-AATT--AAATAAATAATCA--GCGCGC--GCA----C--ATC-CGCGTCGAGA--GAG------TGCGAGGGA--TG--T-----T-AA---A-TAACGATG---T-A------ATG----------ATA-CAGCGG----------GT-------------GC---C-A---CACA-CAA---C--ATTA---CGTTAATGATCCTTCCGCAGGTTCCC-TACGGAAACCTTGTTACGACTTTTACTTCC [2013]

Y._plumbellus CGCCTGTTTATCAAAAACATGTCTTTT-GATTNATAATTTAAAGTCTGGTCTGCCCA-CTGATT---TTATAATTGAAGGGCTGCAGTATATTGACTGTACAAAGGTAGCATAATCATTAGTCTTTTAATTGATGACTAGAATGAATGATCGGATAAAATATAAACTGTCTCTAAATAAATTTATAAAATTTAATTTTTTATTTAAAAAGTTAAAATAATTTTAAAAGACGAGAAGACCCTATAGGGTTTGATAAATATTATAAATTAAAATTATATATAAATA-TTTAATTAAAAATTATAT-ATTTATTTTATTGGGGTGATAGAAAAATTAATTAAACTTTTTTTATAAGTAATACTTATAAACATAAATAATTGATTATATGATCCAAGTTTGA--TTGATTATAAGATTAAATTACCTTAGGGATAACAGCGTAATTTTTTTTTTTAGTTCTTATAAAAAAAAAAGTTTGCGACCTCGATGTTGGATTAAGATAAAATTTAAATGCAAATGTTTAAAATTTTGATCTGTTCGATCATTAAAATCTTA-CATGATCTGAGTTCAAACCGGAGATACCTCGACGTTATTCAGACTATCCAGATGCTTATATTTGTTGGAATATTATTTCTTCATTAGGATCATATATTTCATTTTTAGCAATTATAATAATATTAATTATTGTTTGAGAATCATTTATTTCCCAACGTATAATCTTATTTTCATTAAATATACCTTCTTCTATTGAATGACTTCAAAAATTTCCACCATCAGAACATTCTTATAATGAACTTCCTATTTTAAGAAACTTCTAATATGGCAGATTATATGTAATGGATTTAAACCCCATTTATAAAGGTTTATCCTTTTTTTAGAAATGGCAACATGAACTAATTTAAATTTACAAAATGGAGCATCTCCTTTAATAGAACAAATTATTTTTTTCCATGATCACACATTAATTATTTTAATTATAATTACAATTTTAGTTGGATATTTAATAATCAATCTTTTTTTTAATAAATATACTAATCGATTTTTATTAGAAGGACAAATAATTGAATTAATTTGAACAATTCTACCTGCTATTACTTTAATTTTTATTGCTCTTCCATCATTACGCTTATTATATTTATTAGATGAACTTAATAATCCTTTAATCACATTAAAATCTATTGGCCATCAATGATACTGAAGATATGAATATTCAGATTTTAATAATATTCAATTTGATTCTTATATAATCCCAAGAAAAGAAATAAATATAAATAATTTTCGATTATTAGATGTAGATAATCGAATTATTTTACCTATAAATAATCAAATTCGAATTATAGTAACTGCAACAGATGTTATTCACTCTTGAACGGTTCCTTCTTTAGGGGTTAAAATTGATGCTAATCCAGGACGTTTAAATCAAACAAATTTTTTTATTAATCGACCTGGATTATTTTTTGGTCAATGTTCTGAAATTTGTGGTGCAAATCATAGCTTCATACCTATTGTAATTGAAAGAATTTCAATTAATAATTTTATTAAATGAATTAATAATTATTCGTCATTAGATGACTGAAAGCAAGTAATGGTCTCGCTGCGTTCTTCATCGATGCGCGAGCCAAGTGATCCACCGTCCAGGGTAATGATTT-----------------------------------------------------------------------------------------TCATTG--GT-TCATCAC-CAACAAAGACTGACA----TGATAA-T---AT----------------TATG---ATT--AT-----AATAAAAGTAATA-ATTTTCAAAAT--GAAA----TAACGACAAACGAGCGCTCTC--ACGTTCGCGAN----A------A----ACG-A--GAGGCGC-TCGC-CATCACCGC-------------AGC-G---TTTC-----GTTCAT---AT--TTAACGTCCACT-G-GCGCGTGCGACTCCGGGCGCGGATTTTTTAACCCATCGATGTCGT----------TTAA-ACACG--------TGC-CGT--------------------G--------CGAC------------GTCCCCCTCT-------------T-------CGCC----G---------AC--GGC-G--CG--------------CGCA--A-AAC---AGTG--TAAATCTTCGCGGGC--ACA----C--ATCACGCGTCGAGA--GAGAG----TGCGATGGA--TT--T----GT-AA-----TAACGATG---T-A------AT-------------A-T---------------GT-------------GT--AT-A---CACA-CA----C--GTTAT-ACGTTAATGATCCTTCCGCAGGTTCCC-TACGGAAACCTTGTTACGACTTTTACTTCC [2045]

Y._sedellus CGCCTGTTTATCAAAAACATGTCTTTTTGAATAATAATTTAAAGTCTAGTCTGCCCA-CTGAT----ATTAAATTAAAGGGCTGCAGTATATTGACTGTACAAAGGTAGCCTAATCATTAGTCTTTTAATTGATGACTTGTATGAATGATTGGATAAGATATAAACTGTCTCTTAATAAATTTATAGAATTTAATTTTTTATTTAAAAAGTTAAAATAATTTTAAAAGACGAGAAGACCCTATAGAGTTTAATAA-TTATTTATATTAAAATTATAAATATAAA-TTTAATTAAAATTTATTT-AATTATTTTATTGGGGTGATAAAAAAATTAAAAAAACTTTTTTTAAAATTTA---------ACATAGATAATTGATTATTTGATCCAATTTT---TTTGATTATAAGATTAAATTACCTTAGGGATAACAGCGTAATTTTTTTTTT-AGTTCATATAAAAAAAAAAGTTTGCGACCTCGATGTTGGATTAAGATAAAATTTAAATGCAAAAGTTTAAAATTTTGATCTGTTCGATCATTAAAATCTTA-CATGATCTGAGTTCAAACCGGAGATACCTCGACGTTATTCAGACTATCCAGATGCTTATATTTGTTGAAATATTATTTCTTCATTAGGATCATATATTTCATTTTTAGCAATTATAATAATATTAATTATTGTTTGAGAATCATTTATTTCCCAACGTATAATCTTATTTTCATTAAATATACCTTCTTCTATTGAATGACTTCAAAAATTTCCACCATCAGAACATTCTTATAATGAACTTCCTATTTTAAGAAACTTCTAATATGGCAGATTATATGTAATGGATTTAAACCCCATTTATAAAGGTTTATCCTTTTTTTAGAAATGGCAACATGAACTAATTTAAATTTACAAAATGGAGCATCTCCTTTAATAGAACAAATCATTTTTTTTCATGATCATACATTAATTATTTTAATTATAATTACAATTTTAGTGATATATTTAATAATAAATTTATTTTTTAATAAATACACTAACCGATTTTTATTAGAAGGACAAATAATTGAATTAATTTGAACAATTTTACCTGCAATTACTTTAATTTTTATTGCTCTTCCATCTTTACGTTTATTATATCTACTAGATGAACTTAATAATCCTTTAATTACATTAAAATCAATTGGACATCAATGATATTGAAGTTATGAATATTCAGATTTTAATAATATTCAATTTGATTCATATATAATTCCAAGAAAAGAAATAAATTTAAATAATTTTCGACTTTTAGATGTAGACAATCGTATTATTCTCCCTATAAATAACCAAATTCGTATTATAGTAACAGCTACTGATGTTATTCATTCTTGAACAATCCCTTCTTTAGGGGTAAAAATTGATGCTAATCCAGGTCGTTTAAATCAAACAAACTTTTTTATCAATCGACCTGGATTATTTTATGGTCAATGTTCAGAAATTTGCGGAGCTAATCATAGTTTTATACCTATTGTAATTGAAAGAATTTCAATTAATAATTTTATTAAATGAATTAATAATTATTCTTCATTAGATGACTGAAAGCAAGTAATGGTCTCGCTGCGTTCTTCATCGACCCGCGAGCCAAGTGATCCACCGTCCAGGGTAATGATTT-----------------------------------------------------------------------------------------TCATTG--GT-TCATCAC-CAACAAAGACTGACA----TGATAA-T---AT----------------TATG---ATT--AT-----AATAAAAGTAATA-ATTTTCAAAAT--GAAA----TAACGACAAACGAGCGCTCTC--ACGTTCGCGAA----A------A----ACG-A--GAGGCGC-TCGC-CATCACCGC-------------AGC-G---TTTC-----GTTCAT---AT--TTAACGTCCACT-G-GCGCGTGCGACTCCGGGCGCGGATTTTTTAACCCATCGATGTCGT----------TTAA-ACACG--------TGC-CGT--------------------G--------CGAC------------GTCCCCCTCT-------------T-------CGCC----G---------AC--GGB-G--CG--------------CGCA--A-AAC---AGTG--TAAATSTTCGCGCGC--GCA----C--ATCACGCGTCGAGA--GAGAG----TGCGATGGA--TT--T----GT-AA-----TAACGATG---T-A-------------------ATA-T---------------GT-------------GT--AT-A---CACA-CA----C--GTTAT-ACGTTAATGATCCTTCCGCAGGTTCCCCTACGGAAACCTTGTTACGACTTTTACTTCC [2034]

Y._sedellus__J ------------------------------------------------------------------------------------------------------------------------------------------------------------------------------------------------------------------------------------------------------------------------------------------------------------------------------------------------------------------------------------------------------------------------------------------------------------------------------------------------------------------------------------------------------------------------------------------------ATACCTCGACGTTATTCAGACTATCCAGATGCTTATATTTGTTGAAATATTATTTCTTCATTAGGATCATATATTTCATTTTTAGCAATTATAATAATATTAATTATTGTTTGAGAATCATTTATTTCCCAACGTATAATCTTATTTTCATTAAATATACCTTCTTCTATTGAATGACTTCAAAAATTTCCACCATCAGAACATTCTTATAATGAACTTCCTATTTTAAGAAACTTCTAATATGGCAGATTATATGTAATGGATTTAAACCCCATTTATAAAGGTTTATCCTTTTTTTAGAAATGGCAACATGAACTAATTTAAATTTACAAAATGGAGCATCTCCTTTAATAGAACAAATCATTTTTTTTCATGATCATACATTAATTATTTTAATTATAATTACAATTTTAGTAATATATTTAATAATAAATTTATTTTTTAATAAATACACTAACCGATTTTTATTAGAAGGACAAATAATTGAATTAATTTGAACAATTTTACCTGCAATTACTTTAATTTTTATTGCTCTTCCATCTTTACGTTTATTATATCTACTAGATGAACTTAATAATCCTTTAATTACATTAAAATCAATTGGACATCAATGATATTGAAGTTATGAATATTCAGATTTTAATAATATTCAATTTGATTCATATATAATTCCAAGAAAAGAAATAAATTTAAATAATTTTCGACTTTTAGATGAAGACAATCGTATTATTCTCCCTATAAATAACCAAATTCGTATTATAGTAACAGCTACTGATGTTATTCATTCTTGAACAATCCCTTCTTTAGGGGTAAAAATTGATGCTAATCCAGGTCGTTTAAATCAAACAAACTTTTTTATCAATCGACCTGGATTATTTTATGGTCAATGTTCAGAAATTTGCGGAGCTAATCATAGTTTTATACCTATTGTAATTGAAAGAATTTCAATTAATAATTTTATTAAATGAATTAATAATTATT----------------------------------GCTGCGTTCTTCATCGATGCGCGAGCCAAGTGATCCACCGTCCAGGGTAATGATTT----------------------------------------------------------------------------------------------G--GT-TCATCA----CC-----TTG-TG----TGATAG-T---GC--------------GATACG---AAT--AT---GAAATACACTTAA-ACATTTTT---ATT-G-TA----TAACGACAAACGAGCGCTCTCGCACGCTT-CGTC----A----GAA----ACG-ATAGAGGCGC-TCGC-CATCACCGC-------------AGA-G----TT-TT-TCGTTAGT---GT--TTAACGTCCACTCG-GCGCGTGCGACTCCGGGCGCGGATTT-GTG-CCCATCGATGTCGT----------TTAA-ACACG--------TGC-CGC--------------------GG-------CGAC------------GTCTC--TCT-------------T-------CGCC----G---------AC--AGC-GG-CG--------------CGTC-TG-CACG--CGC-------------CGCGT------------ATC-CACGTCGAGA--GAGAG----TGCGAGGGA--TG--T----GT-AA-----TAACGATGCAGT-AGCAT--ATGTAT-------ATA-CAGCGGGCGCT-----GT--AT---------GC--AC-A---CACA-CAAA--C--GTTA---CGTTAATGATCCTTCCGCAGGTTCCC-TACGGAAACCTTGTTACGACTTTTACTTCC [1449]

Y._yanagawanus CGCCTGTTTATCAAAAACATGTCTTTTTGAATAATAATTTAAAGTCTAGTCTGCCCA-CTGATT---ATTAAATTAAAGGGCTGCAGTATATTGACTGTACAAAGGTAGCATAATCATTAGTCTCTTAATTGATGACTTGTATGAATGATTGGATAAGATATAAACTGTCTCTTAATGAATTTATAGAATTTAATTTTTTATTTAAAAAGTTAAAATAGTTTTAAAAGACGAGAAGACCCTATAGAGTTTAATAT-TTATTTATATTAAAATTATATGTATAAA-TTTAATTAAAATTTATTT-AATTATTTTATTGGGGTGATAAAAAAATTAAAAAAACTTTTTTTAAAATTTA---------ACATAGATAATTGATTATTTGATCCAATATT---TTTGATTATAAGATTAAATTACCTTAGGGATAACAGCGTAATTTTTTTTTTTAGTTCGTATAAAAAAAAAAGTTTGCGACCTCGATGTTGGATTAAGATAAAATTTAAATGCAAAAGTTTAAAATTTTGATCTGTTCGATCATTAAAATCTTA-CATGATCTGAGTTCAAACCGGAGATACCTCGACGTTATTCAGATTACCCTGATGCTTATATTTGTTGAAATATTATTTCTTCTTTAGGTTCTTATATTTCATTTTTAGCAATTATAATAATATTAATTATTGTTTGAGAATCCTTTATTTCTCAACGTATAATCTTATTTTCATTAAATATATCTTCTTCTATTGAATGACTTCAAAAATTCCCACCATCAGAACATTCCTATAATGAACTTCCTATCTTAAGAAACTTCTAATATGGCAGATTATATGTAATGGATTTAAACCCCATTTATAAAGGTTTATCCTTTTTTTAGAAATGGCAACATGAACTAATCTAAATCTACAAAATGGAGCATCTCCTTTAATAGAACAAATCATTTTTTTTCACGATCACACATTAATTATTTTAATTATAATTACAATTTTAGTAGGATATTTAATAATCAATTTATTTTTTAATAAATATACTAATCGATTTTTATTAGAAGGACAAATAATTGAATTAATTTGAACAATTTTACCTGCAATTACTTTAATTTTCATTGCTCTTCCATCTTTACGTTTACTATATTTATTAGATGAACTTAATAATCCTTTAATTACATTAAAATCAATTGGACATCAATGATATTGAAGTTATGAATATTCAGATTTTAATAATATTCAATTTGACTCATATATAATCCCAAGAAAAGAAATAAATTTAAATAATTTTCGACTTTTAGATGTAGATAATCGTATTATTCTTCCTATAAATAATCAAATTCGTATTATAGTGACAGCTACTGATGTTATCCACTCTTGAACAATTCCATCTCTAGGAGTAAAAATTGATGCTAATCCAGGTCGTTTAAATCAAACAAACTTTTTTATTAATCGACCTGGATTATTTTATGGTCAATGTTCAGAAATTTGTGGAGCTAATCATAGTTTTATACCTATTGTAATTGAAAGAATTTCAATTAATAATTTTATTAAATGAATTAATAATTATTCTTCATTAGATGACTGAAAGCAAGTAATGGTCTCGCTGCGTTCTTCATCGATGCGCGAGCCAAGTGATCCACCGTCCAGGGTAATGATTT----------------------------------------------------------------------------------------------G--GT-TCATCAC-C--------TTGT-------GATAAGT---GC--------------GATACG---AAT--AT---GAAATACACTTAAAACATTC----AATT-G-TA----TAACGACAAACGAGCGCTCTCGCACGCTT-CGTT----A----GAA----ACG-ATAGAGGCGC-TCGC-CATCACCGC-------------GGA-G----TT-TT-TCGTTCAT---GT--TTAACGTCCGCGCG-GCGCGTGCGACTCCGGGCGCGGATTT-GTG-CCCATCGACGTCGT----------TTAA-ACACG--------TGC-CGC--------------------GG-------CGAC------------GTCTC--TCT-------------T-------CGCC----G---------AT--AGC-GTGCG--------------CGTC-TG-GACG--CGT-------------CGCGT------------ATC-CGCGTCGAGA--GAGAG----TGCGAGGGA--TG--T----GT-AA-----TAACGATGCAGT-AGTAT--ATGTAT-------ATA-CAGCGG-CGCT-----GT--AT---------GT--AC-A---CACA-CAAA--C--GTTA---CGTTAATGATCCTTCCGCAGGTTCCCCTACGGAAACCTTGTTACGACTTTTACTTCC [2039]

Y._eurinellus CGCCTGTTTATCAAAAACATGTCTTTTTGAAGTATAATTTAAAGTCTGGTCTGCCCA-CTGAT----AAATAATTAAAGGGCTGCAGTATATTGACTGTACAAAGGTAGCATAATCATTAGTCTTTTAATTGGTGACTTGTATGAATGATCGGATAAAATATAAGCTGTCTCTAGATAAATTTATAGAATTTAATTTTTTATTTAAAAAGTTAAAATGATTTTAAAAGACGAGAAGACCCTATAGAGTTTAATAT-TTATTTAAATTGAAATTATATATATTAACTTTAATTAAAAATTAAAT-ATTTATTTTATTGGGGTGATAAAAAAATTAATTAAACTTTTCTTAAAATTTA---------ACATAAATAATTGAATATTTGATCCAATTTT---TTTGATTATAAGATTAAATTACCTTAGGGATAACAGCGTAATTTTTTTTTTTAGTTCTTATAAAAAAAAAAGTTTGCGACCTCGATGTTGGATTAAGATAAAATTTAAATGCAAAAGTTTAAAATTTTGATCTGTTCGATCATTAAAATCTTA-CATGATCTGAGTTCAAACCGGAG-----------------------------------------------------------------------------------------------------------------------------------------------------------------------------------------------------------------------------------------------------------------------------------------------------------------------------------------------GGAGCATCTCCTTTAATAGAACAAATCATTTTCTTTCACGATCATACTTTAATTATTTTAATTATAATTACAATTTTAGTGGGATATTTAATAATTAATTTATTTTTCAATAAATATACTAATCGTTTCTTATTAGAAGGACAAATAATTGAATTAATTTGAACAATTTTACCTGCAATTACTTTAATTTTTATTGCTCTTCCATCTTTACGTTTATTATATTTATTAGATGAATTAAATAATCCATTAATTACATTAAAATCAATTGGACATCAATGATATTGAAGCTATGAATATTCAGATTTTAATAATATTCAATTTGACTCTTATATAATTCCAAATAAAGAAATAAAAATTAATAACTTTCGATTATTAGATGTAGATAATCGTATTATTCTTCCTATAAATAATCAAATTCGTATTATAGTGACAGCAACAGATGTTATTCATTCTTGAACAATCCCATCTTTAGGAGTTAAAATTGATGCTAACCCAGGACGTTTAAATCAAACTAATTTTTTTATCAATCGACCTGGATTATTTTATGGTCAATGTTCTGAAATTTGTGGAGCAAATCATAGTTTTATACCTATTGTAATTGAAAGAATTTCAATTAATAATTTTATTAAATGAATTAATAACTATTCCTCATTAGATGACTGAAAGCAAGTAATGGTCTCGCTGCGTTCTTCATCGATGCGCGAGCCAAGTGATCCACCGTCCAGGGTAATGATTT----------------------------------------------------------------------------------------------G--GT-TCATCAC-CA-CACACGTTGTTGCGCGT-GTTG-T---GC--------------GATATG---AGT--ATTATAAAATAGA---AT-ACATTT-----ATT---AA----TAACGACAAACGAGCGCTCTC--ACGCT---GTT----A------A----ACG-A--GAGGCGC-TCGC-CATCACCGC-------------AGA-G----TT-TT---GTTTAT---GT--TTAACGTCCACT-G-GCACGTGCGACTCCGGGCGCGGATTTTC-GACCCATCGATGTCGT----------TTA-GACACG--------TGC-CGT--------------------G--------CGAC------------GTCTC--TCT-------------T-------CGCC----G---------ATT-GGC-G--CG--------------CGC---G-CGCG--CGCG--TTGA-C---GCGCGCCCGCACACAC--ATC-TACGTCGAGA--GAG------TGAGAGGGA--TG--T-----G-AA---A-TAACGATG---T-A------ATG----------ATA-CAGCGGC----------T-------------GC---C-G---TACA-CAA---C--ATTA---CGTTAATGATCCTTCCGCAGGTTCCCCTACGGAAACCTTGTTACGACTTTTACTTCC [1696]

Xyrosaris_lichneuta CGCCTGTTTATCAAAAACATGTCTTTTTGTGAAATAATTTAAAGTCTGGTCTGCCCA-CTGA---A-ATTTTTTTGAAGGGCTGCAGTATATTGACTGTACAAAGGTAGCATAATCAGTAGTCTTTTAATTGATGACTTGTATGAATGATCGGATAAGATATAAACTGTCTTAAATAGATTATATTGAATTTAATTTTTTATTTAAAAAGTTAAAATAATTTTAAAAGACGAGAAGACCCTATAAAGTTTAATAT-TCTATTAAATTAAAATTTTTTATTAAAT-TTTAATTAAAATTTAATA-AAATATTTTGTTGGGGTGATAAAAAAATTAAAAAAACTTTTTTTTTATAAAA---------ACATAAATAAATGAATTATTGATCCATTTTT--TTTTGATTAAAAGATTAAATTACTTTAGGGATAACAGCGTAATCTTTTTTTTTAGTTCTTATAAGAAAAAAAGTTTGCGACCTCGATGTTGGATTAAGATAAAATTTAAATGCAAAAGTTTAAAATTT-GATCTGTTCGATCATTAAAATCTTA-CATGATCTGAGTTCAAACCGGAGATACCTCGACGTTATTCAGATTACCCTGATTCTTACATTTGTTGAAATATTATTTCTTCTTTAGGATCTTATATTTCTTTTTTAGGAATTATAATAATATTAATTATTATTTGAGAATCTATAATTTCTCAACGAATAAGATTATTTTCTCTAAATATACCTTCCTCTATTGAATGATTGCAAAATTTCCCCCCCGCTGAACATTCATATAATGAACTTCCTATTTTAAGAAATTTCTAATATGGCAGATTATATGTAATGGATTTAAACCCCATTTATAAAGGATCATCCTTTTTTTAGAAATGGCAACATGAATAAATTTAAATCTCCAAAATGGAGCATCTCCTTTAATAGAACAAATTATTTTTTTTCATGACCACACTTTAATAATTTTAATTATAATTACTATTTTAGTTAGTTATTTAATAATTAATTTATTTTTTAATAAATATATTAACCGATTTTTATTAGAAGGGCAAATAATTGAATTAATTTGAACTATTTTACCTGCTATTACTTTAATTTTTATTGCTTTACCATCTTTACGATTATTATATTTATTAGATGAAATTAATAACCCTTTAATTACATTAAAATCAATTGGACATCAATGATATTGAAGTTATGAATACTCAGATTTTAATAATATTCAATTTGATTCTTATATAATTCCAAGAAACGACTTAAAAATAAGAGATTTTCGTTTACTAGATGTTGATAATCGAATTATTTTACCTATAAATAATCAAATTCGTATTATAGTAACTGCTACTGATGTAATTCATTCTTGAACAATTCCTTCATTAGGAGTAAAAATTGATGCTAATCCAGGTCGTTTAAATCAAACTAATTTTTTTATTAATCGTCCTGGATTATTTTATGGACAATGTTCAGAAATTTGTGGAGCAAATCATAGATTTATACCTATTGTAATTGAAAGAATTTCAATTAATAATTTTATTAAATGAATTAATAATTATTCTTCATTAGATGACTGAAAGCAAGTAATGGTCTCGCTGCGTTCTTCATCGATGCGCGAGCCAAGTGATCCACCGTCCAGGGTAATGATTT---------------------------------------------------------------------------------------------TGG-GCATTACCAC-CAT-----ACCACCACCCAACATGATT---GT--------------GATG------AT--ATCC-AAAAT--ACACAA-A-ATTG---AAAT--GAAACTTTTAACGACGAACGAGCGCACGC--ATTCC--CGT-----AT--------TCACGGA---AGGCGC-TCGTGCATCACCGC-------------------AC--TGT-GA-GATTAT---AT--TTAACGTCC-CGCG-ACACGTGCGACTCCGGGTGCGGATTT-GTTACCCATCGATGTCGTCGTTTAACCCTTAAAAGACCAATTTAATTGCTTTT--------------------GCTTTAAAACAACATTAGGCGTTGTGTTTTTTTTTTTTTTTTTTTTTTTTATATAGCCTCTACTGTGTTTTAAAACAACATGGGGCGTTTAAGGGTTAAACACGTGTGTCGCATGCGACG----------GCGCGA---CA----C-GCACACA-GTCAAG-T-GTAT----CT-CGCGCGAGTTGGATG---GTTTA--TA-TAACGGTG---T-A--------------------TA-TAACGGCGC--------TC------------GC---TCA---CGCGGCACTG-CT-ATTATTACGTTAATGATCCTTCCGCAGGTTCCCCTACGGAAACCTTGTTACGACTTTTACTTCC [515]

Y._spodocrossus CGCCTGTTTATCAAAAACATGTCTTTTTGAAAAATAATTTAAAGTCTGGTCTGCCCA-CTGATT-ATTTATAATTAAAGGGCTGCAGTATTTTGACTGTACAAAGGTAGCATAATCATTAGTCTTTTAATTGGTGACTTGTATGAATGATCGGATAAAATATAAACTGTCTCTTAATAAATTTATAGAATTTAATTTTTTATTTAAAAAGTTAAAATAATTTTAAAAGACGAGAAGACCCTATAGAGTTTAATAT-TTGTATAAATTAAAATTATATATAAAAA-TTTAATTAAAAAATTATATATTTATTTTATTGGGGTGATAAAAAAATTAA-AAAACTTTTTTTAAATTTTA---------ACATATATAATTGAATATTTGATCCAATTTT--TTTTGATTATAAGATTAAATTACCTTAGGGATAACAGCGTAATTTTTTTTTTTAGTTCTTATAAAAAAAAAAGTTTGCGACCTCGATGCTGGATTAAGATAAAATTTAAATGCAAAAGTTTAAAATTTTGATCTGTTCGATCATTAAAATCTTA-CATGATCTGAGTTCAAACCGGAGATACCTCGACGTTATTCAGATTATCCAGATGCTTATATTTGTTGAAATATTATTTCTTCATTAGGATCTTATATTTCATTTTTAGCAATTATAATAATATTAATCATCATTTGAGAATCATTTATTTCTCAACGAATAATTTTATTTTCATTAAATATATCTTCTTCTATTGAATGACTTCAAAAATTTCCACCATCAGAACATTCTTATAATGAACTTCCTATTTTAAGAAATTTCTAATATGGCAGATAATATGTAATGGATTTAAACCCCATTTATAAAGGTTAATCCTTTTTTTAGGAATGGCAACATGAAATAATTTAAATTTACAAAATGGAGCATCTCCTTTAATAGAACAAATCATTTTTTTTCATGATCACACATTAATTATCTTAATTATAATTACAATTTTAGTAGGATATTTAATAATTAATTTATTTTTTAATAAATATACAAATCGATTTTTATTAGAAGGACAAATAATTGAACTAATTTGAACAATTTTACCAGCAATCACTTTAATTTTTATTGCTCTTCCATCTCTTCGTTTACTATATTTACTAGATGAACTAAATAATCCTTTAATTACATTAAAATCTATTGGTCATCAATGATATTGAAGTTATGAATATTCAGATTTTAATAATATTCAATTTGACTCATATATAATCCCAAGAAAAGAAATAAGAATTAATAATTTTCGATTATTAGATGTAGATAATCGTATTATTCTCCCTATAAATAATCAAATTCGTATTATAGTAACAGCAACAGATGTAATTCTTTCTTGAACAATTCCATCATTAGGTGTTAAAATTGATGCTAATCCAGGACGATTAAATCAAACTAATTTTTTTATTAATCGTCCTGGTTTATTTTATGGTCAATGTTCTGAAATTTGTGGAGCAAATCATAGTTTTATACCTATTGTAATTGAAAGAATTTCAATTAATAATTTTATTAAATGAATTAATAATTATTCTTCATTAGATGACTGAAAGCAAGTAATGGTCTCGCTGCGTTCTTCATCGATGCGCGAGCCAAGTGATCCACCGTCCAGGGTGATTATTT----------------------------------------------------------------------------------------------GT-GTTTGTTCGG-C----------G---------ATACTT---GC--------------GATA-----AAT---T----AAAACGAAACATTAAATTT-----AT-AGAAA----TGACGACAAACGAGCGCTCTC--ACGCCGCCGCC----ACGTTGAACGCAACG-A--GAGGCGC-TCGC-CATCACCGC-------------AGG-G----TTCTT---GTTTAT---AT--TTAACGTCCACT-G-GCAGGTGCGACTCCGGGCGCGGATTTTA-GACCCATCGATGTCGT----------TTGAGACACT--------TGC-CGT--------------------G--------CGAC------------GTCCC--TCT---------------------CGTC----GT--------ACCTCCC-GT-CGA-----------ACCGTAAGG-TGTGTGTGT------------GCGNGCGCCCA----C--CTC-TACGTCGAGA--GAACGC-ACT-CGAGGGA--TAAATT---GTGAAA-AA-TAACGAT----TCA-------------------ATTGCA-C------------GT--------------C--AT-A---CGCG-CTCT--C--GACA---CGTTAATGATCCTTCCGCAGGTTCCCCTACGGAAACCTTGTTACGACTTTTACTTCC [2041]

Y._sociatus CGCCTGTTTATCAAAAACATGTCTTTTTGAA-AATAATTTAAAGTCTGGTCTGCCCA-CTGATT-ATAAATAATTAAAGGGCTGCAGTATATTGACTGTACAAAGGTAGCATAATCATTAGTCTTTTAATTGATGACTTGTATGAATGATCGGATAAAATATAAACTGTCTCTTAATAAATTTATAGAATTTAATTTTTTATTTAAAAAGTTAAAATAATTTTAAAAGACGAGAAGACCCTATAGAGTTTAATAA-TTTTGTAAATTAAAATTATATGTATAAA-TTTAATTAAAAATTATAG-ATTTATTTTGTTGGGGTGATAAAAAAATTAATTAAACTTTTTTTAAAATTTA---------ACATAAATAATTGAATATTTGATCCAATTTT---TTTGATTATAAGAATAAATTACCTTAGGGATAACAGCGTAATTTTTTTTTTTAGTTCTTATAAAAAAAAAAGTTTGCGACCTCGATGTTGGATTAAGATAAAATTTAAATGCAAAAGTTTAAATTTT-GATCTGTTCGATCATTAAAATCTTA-CATGATCTGAGTTCAAACCGGAGATACCTCGACGTTATTCAGATTATCCAGATGCTTATATTTGTTGAAATATTATTTCTTCATTAGGATCTTATATTTCATTTTTAGCAATTATAATAATATTAATCATTATTTGAGAATCATTTATTTCTCAACGAATAATTTTATTTTCATTAAATATATCTTCTTCTATTGAATGACTTCAAAAATTCCCACCATCAGAACATTCTTATAATGAACTTCCTATTTTAAGAAATTTCTAATATGGCAGATAATATGTAATGGATTTAAACCCCATTTATAAAGGTTAATCCTTTTTTTAGAAATGGCAACATGAAATAATTTAAATTTACAAAATGGAGCATCTCCTTTAATAGAACAAATCATTTTTTTTCATGATCATACATTAATTATTTTAATTATAATTACAATTTTAGTAGGATATTTAATAATTAATTTATTCTTTAATAAATATACAAATCGATTTTTATTAGAAGGACAAATAATTGAATTAATTTGAACAATTTTACCAGCAATCACTTTAATTTTTATTGCTCTTCCATCTCTTCGTTTATTATATTTACTAGATGAATTAAATAATCCTTTAATTACATTAAAATCTATTGGTCACCAATGATATTGAAGTTATGAATATTCAGATTTTAATAATATTCAATTTGACTCATATATAATTCCAAGAAAAGAATTAAGAATTAATAATTTTCGATTATTAGATGTAGATAATCGTATTATTCTCCCTATAAATGATCAAATTCGAATTATAGTAACAGCAACAGATGTAATTCATTCTTGAACGGTTCCATCATTAGGAGTAAAAATTGATGCTAATCCAGGACGATTAAATCAAACTAATTTTTTTATTAATCGTCCTGGATTATTTTATGGTCAATGTTCTGAAATTTGTGGAGCAAATCATAGTTTTATACCTATTGTAATTGAAAGAATTTCAATTAATAATTTTATTAAATGAATTAATAATTATTCTTCATTAGATGACTGAAAGCAAGTAATGGTCTCGCTGCGTTCTTCATCGATGCGCGAGCCAAGTGATCCACCGTCCAGGGTGATTATTT----------------------------------------------------------------------------------------------GT-GTTTGTTCGG-C----------G---------ATACTT---GC--------------GATA-----AAT---T----AAAACGAAACATTAAAT-TT----AT-AGAAA----TGACGACAAACGAGCGCTCTC--ACGCCGCCGCC----ACGTTGAACGCAACG-A--GAGGCGC-TCGC-CATCACCGC-------------AGG-G----TTCTT---GTTTAT---AT--TTAACGTCCACT-G-GCAGGTGCGACTCCGGGCGCGGATTTTA-GACCCATCGATGTCGT----------TTAAGACACT--------TGC-CGT--------------------G--------CGAC------------GTCCC--TCT---------------------CGTC----GT--------ACCTCCC-GT-CGA------------CCG-A-GG-CGCG--CGC------------GCGCGCC--CA----C--CTC-TACGTCGAGA--GAACGC-ACT-CGAGGGA--TAAATT---GTGAAA-AA-TAACGAT----TCA-------------------AT--T-GCA--C--------GT--------------C--AT-A---CGCG-CTCT--C--GATA---CGTTAATGATCCTTCCGCAGGTTCCCCTACGGAAACCTTGTTACGACTTTTACTTCC [2031]

Y._polystigmellus CGCCTGTTTATCAAAAACATGTCTTTTTGAA-AATAATTTAAAGTCTGGTCTGCCCA-CTGATTTATAAATAATTAAAGGGCTGCAGTATATTGACTGTACAAAGGTAGCATAATCATTAGTCTTTTAATTGATGACTAGTATGAATGATCGGATAAAATATAAACTGTCTCTTAATAAATTTATAGAATTTAATTTTTTATTTAAAAAGTTAAAATAATTTTAAAAGACGAGAAGACCCTATAGAGTTTAATAA-TTTTATAAATTAAAATTATATGTATAAA-TTTAATTAAAAATTATAA-ATTTATTTTGTTGGGGTGATAAAAAAATTAATTAAACTTTTTTTAAAATTTA---------ACATAAATAATTGAATATTTGATCCAATTTT---TTTGATTATAAGAATAAATTACCTTAGGGATAACAGCGTAATTTTTTTTTTTAGTTCTTATAAAAAAAAA-GTTTGCGACCTCGATGTTGGATTAAGATAAAATTTAAATGCAAAAGTTTAAAATTTTGATCTGTTCGATCATTAAAATCTTA-CATGATCTGAGTTCAAACCGGAGATACCTCGACGTTATTCAGATTATCCAGATGCTTATATTCGTTGAAATATTATTTCTTCATTAGGATCTTATATTTCATTTTTAGCAATTATAATAATACTAATCATTATTTGAGAATCATTTATTTCTCAACGAATAATTTTATTTTCATTAAATATATCTTCTTCTATTGAATGACTTCAAAAATTCCCACCATCAGAACATTCTTATAATGAACTTCCTATTTTAAGAAATTTCTAATATGGCAGATAATATGTAATGGATTTAAACCCCATTTATAAAGGTTAATCCTTTTTTTAGAAATGGCAACATGAAATAATTTAAATTTACAAAATGGAGCATCTCCTTTAATAGAACAAATCATTTTTTTTCATGATCATACATTAATTATTTTAATTATAATTACAATTTTAGTAGGATATTTAATAATTAATTTATTCTTTAATAAATATACAAATCGATTTTTATTAGAAGGACAAATAATTGAATTAATTTGAACAATTTTACCAGCAATCACTTTAATTTTTATTGCTCTTCCATCTCTTCGTTTATTATATTTACTAGATGAATTAAATAATCCTTTAATTACATTAAAATCTATTGGTCACCAATGATATTGAAGTTATGAATATTCAGATTTTAATAATATTCAATTTGACTCATATATAATTCCAAGAAAAGAAATAAGAATTAATAATTTTCGATTATTAGATGTAGATAATCGTATTATTCTCCCTATAAATAATCAAATTCGAATTATAGTAACAGCAACAGATGTAATTCATTCTTGAACGGTTCCATCATTAGGAGTAAAAATTGATGCTAACCCAGGACGATTAAATCAAACTAATTTTTTTATTAATCGTCCTGGATTATTTTATGGTCAATGTTCTGAAATTTGTGGGGCAAATCATAGTTTTATACCTATTGTAATTGAAAGAATTTCAATTAATAATTTTATTAAATGAATTAATAATTATTCTTCATTAGATGACTGAAAGCAAGTAATGGTCTCGCTGCGTTCTTCATCGATGCGCGAGCCAAGTGATCCACCGTCCAGGGTGATTATTT--------------------------------------------------------------------------------------------TTGT-GTCTGTTCGG-C----------G---------ATACTT---GC--------------GATA-----AAT---T----AAAACGAAACATTACATTT-----AT-AGAAA----TGACGACAAACGAGCGCTCTC--ACGCCGCCGCC----ACGTTGAACGCAACG-A--GAGGCGC-TCGC-CATCACCGC-------------AG--G----TTCTT---GTTTAT---AT--TTAACGTCCACT-G-GCAGGTGCGACTCCGGGCGCGGATTTTA-GACCCATCGATGTCGT----------TTAAGACACT--------TGC-CGT--------------------G--------CGAC------------GTCCC--TCT---------------------CGTC----GT--------ACCTCCC-GT-CGA------------CCGAT-GG-GGCG--TGC------------GCGCGCC--CA----C--CTC-TACGTCGAGA--GAACGC-ACT-CGAGGGA--TAAATT---GTGAAA-AA-TAACGAT----TCA-------------------ATTGCA-C------------GT--------------C--AT-A---CGCG-CT----C--GATA---CGTTAATGATCCTTCCGCAGGTTCCC-TACGGAAACCTTGTTACGACTTTTACTTCC [2031]

Y._griseatus CGCCTGTTTATCAAAAACATGTCTTTTTGATTAATAATTTAAAGTCTGGTCTGCCCA-CTGATT-A-AAATAATTAAAGGGCTGCAGTATTTTGACTGTACAAAGGTAGCATAATAATTAGTCTTTTAATTGATGACTTGTATGAATGATTAGATAAAATATAAGCTGTCTCTTAATAAATTTATAGAATTTAATTTTTTATTTAAAAAGTTAAAATAATTTTAAAAGACGAGAAGACCCTATAGAGTTTAATAA-TTTTATAAATTAAAATTGTATTTATAAA-TTTAATTAAAAATTATAT-AATTATTTTATTGGGGTGATAAAAAAATTAATAAAACTTTTTTTAAATATTA---------ACAAAAATAATTGAATATTTGATCCAATTTT-ATTTTGATTATAAGATTAAATTACCTTAGGGATAACAGCGTAATTTTTTTTTTTAGTTCTTATAAAAAAGAAAGTTTGCGACCTCGATGTTGGATTAAGATAAAATTTAAATGCAAAAGTTTAAAATTTTGATCTGTTCGATCATTAAAATCTTA-CATGATCTGAGTTCAAACCGGAG-------------------------------------------------------------------------------------------------------------------------------------------------------------------------------------------------------------------------------------------------------------------------------------------------------------------------------------------------------------------------------------------------------------------------------------------------------------------------------------------------------------------------------------------------------------------------------------------------------------------------------------------------------------------------------------------------------------------------------------------------------------------------------------------------------------------------------------------------------------------------------------------------------------------------------------------------------------------------------------------------------------------------------------------------------GCTGCGTTCTTCATCGATGCGCGAGCCAAGTGATCCACCGTCCAGGGTAATGATTT----------------------------------------------------------------------------------------------G--GC-TCATCAC-CATCACAAGATATT-------ATAA-T---GCTTGTGTTCTCTGTCGATGTGTCAAGTGAATT--GAAGT-GAAATAA-AGA--------AA----AA----TAACGACAAACGAGCGCACTC--ACGCC---GTC----A------A----GCG-A--GAGGCGC-TCGC-CATCACCGC-------------GGT-G----TT-TT---GTGTGT---ATATTTAACGTCCAC-CG-GCACGTGCGACTCCGGGCGCGGATTTTG-AACCCATCGATGTCGT----------TTA-GACGCG--------TGC-CGT--------------------G--------CGAC------------GTCTC--TCT-------------T-------CGCC----G---------AT--CGC-G--CG--------------CGC---G-AGCG--AGCG-------C---GCGTGC--GCA----G--ATC-TACGTCGAGA--GAA------TGCGAGGGA--TG--AC---AT-AG---A-TAACGATG---T-A------ATG----------ATA-TAGCGGCGGCGGCAGAGTTGCTGCTGCTGCTGC---T-A---CACA-CA----C--GTTA---CGTTAATGATCCTTCCGCAGGTTCCCCTACGGAAACCTTGTTACGACTTTTACTTCC [1044]

Y._kanaiellus -------------------------------------------------------------------------------------------------------------------------------------------------------------------------------------------------------------------------------------------------------------------------------------------------------------------------------------------------------------------------------------------------------------------------------------------------------------------------------------------------------------------------------------------------------------------------------------------------------------------------------------------------------------------------------------------------------------------------------------------------------------------------------------------------------------------------------------------------------------------------------------------------------------------------------------------------------------------------------------------------------------------------------------------------------------------------------------------------------------------------------------------------------------------------------------------------------------------------------------------------------------------------------------------------------------------------------------------------------------------------------------------------------------------------------------------------------------------------------------------------------------------------------------------------------------------------------------------------------------------------------------------------------GCTGCGTTCTTCATCGATGCGCGAGCCAAGTGATCCACCGTCCAGGGTAATGATTT----------------------------------------------------------------------------------------------G--GT-T-ATCTC-T--C-------GT---------TACGT---GC--------------GATA-----AGT---TA--AAAAT-GAAATAA-A-A--------AT-AAAAA----TAACGACAGACGAGCGCTCTCTCGCGCC---GTTG---A-G----A----ACG-A--GAGGCGC-TCGC-CATCACCGC-------CGAACAAGAAGAAC-TTCTTGTCGTTTATTATATATTTAACGTCCAC-CG-GCAGGTGCGACTCCGGGCGCGGATTTTA-GACCCATCGATGTCGT----------TTAGGACACC--------TGC-CGT--------------------G--------CGAC------------GTCCC--TCTCGTGTAGTCCCTCT-------CGTC----G---------AC--GGA-GG-CG--------------CGCC--G-CGCG--CGT----CGA-----GAGAGC--ACA----C--GCCTTGCCTCGAGA--GAACAC-ACT-CGAGGGA-ATG--TG---GGCGAT-TA-TAACGAT----TCAATCGTGTTGTAT-------ATAGCGGCGGCCGCGGCTGAGTCGCTGCTGC----GCTAAT-A---CACA-CACT--C--GATA---CGTTAATGATCCTTCCGCAGGTTCCCCTACGGAAACCTTGTTACGACTTTTACTTCC [2088]

Y._tokyonellus CGCCTGTTTATCAAAAACATGTCTTTTTGAA-AATAATTTAAAGTCTGGTCTGCCCA-CTGATTTATTAATAATTAAAGGGCTGCAGTATATTGACTGTACAAAGGTAGCATAATAATTAGTCTTTTAATTGATGACTTGTATGAATGATCGGATAAAATATAAACTGTCTCTTAATAAATTTATAGAATTTAATTTTTTATTTAAAAAGTTAAAATAATTTTAAAAGACGAGAAGACCCTATAGAGTTTAATAA-TTTTATAAATTAAAATTATATATATAAA-TTCAATTAAAAATTATAA-ATTTATTTTGTTGGGGTGATAAAAAAATTAATTAAACTTTTTTTAAAATTTA---------ACATAAATAAATGAATATTTGATCCAATTTTTTTTTTGATTATAAGAATAAATTACCTTAGGGATAACAGCGTAATTTTTTTTTTTAGTTCTTATAAAAAAAAAAGTTTGCGACCTCGATGTTGGATTGAGATAAAATTTAAATGCAAAAGTTTAAAATTTTGATCTGTTCGATCATTAAAATCTTA-CATGATCTGAGTTCAAACCGGAGATACCTCGACGTTATTCAGATTATCCTGATACTTATATTTGTTGAAATGTTATTTCTTCATTAGGATCTTATATTTCATTTTTAGCAATTATAATAATATTAATTATTATTTGAGAATCATTTATTTCTCAACGAATAATTTTATTTTCATTAAATATATCTTCTTCTATTGAATGACTTCAAAAATTTCCACCATCAGAACATTCTTATAATGAACTTCCTATTTTAAGAAATTTCTAATATGACAGATAATATGTAATGGATTTAAACCCCATTTATAAAGGTTAATCCTTTTTTTAGAAATGGCAACATGAAATAATTTAAATTTACAAAATGGAGCATCTCCTTTAATAGAACAAATCATTTTTTTTCATGATCATACATTAATCATTTTAATTATAATTACAATTTTAGTAGGATATTTAATAATTAATTTATTTTTTAATAAATATACAAATCGATTTTTATTAGAAGGACAAATAATTGAGTTAATTTGAACAGTCTTACCAGCAATTACTTTAATTTTTATTGCTCTTCCATCTCTTCGTTTATTATATTTATTAGATGAATTAAATAATCCTTTAATCACATTAAAATCAATTGGTCATCAATGATATTGAAGTTATGAATATTCAGATTTTAATAATATTCAATTTGATTCATATATAATCCCAAGAAAAGAAATAAGAATTAATAATTTTCGATTATTAGATGTAGATAATCGAATTATTCTTCCTATAAATAATCAAATTCGTATTATAGTAACAGCAACAGATGTAATTCATTCTTGAACAGTTCCATCATTAGGAGTAAAAATTGATGCTAATCCAGGACGATTAAATCAAACTAATTTTTTTATTAATCGTCCTGGATTATTTTATGGTCAATGTTCTGAAATTTGTGGAGCAAATCATAGTTTTATACCTATTGTAATTGAAAGAATTTCAATTAATAATTTTATTAAATGAATTAATAATTATTCTTCATTAGATGACTGAAAGCAAGTAATGGTCTCGCTGCGTTCTTCATCGATGCGCGAGCCAAGTGATCCACCGTCCAGGGTAATGATTT----------------------------------------------------------------------------------------------G--GT-T-ATCTC------------GT---------TACGT---GC--------------GATA-----AGT---TG--AAAAT-GAAATAA-A-A--------AT-AAAAA----TAACGACAGACGAGCGCTCTCTCGCGCC---GTTG---A-G----A----ACG-A--GAGGCGC-TCGC-CATCACCGC-------CGAACAAGAAGAAC-TTCTTGTCGTTTATTATATATTTAACGTCCAC-CG-GCAGGTGCGACTCCGGGCGCGGATTTTA-GACCCATCGATGTCGT----------TTAGGACACC--------TGC-CGT--------------------G--------CGAC------------GTCCC--TCTCGTGTAGTCCCTCT-------CGTC----G---------AC--GGAGGCGCG-------------CCGC---G-CGCGT-CGA------------GCGGGC--ACA----C--GCCTTGCCTCGAGA--GAACAC-ACT-CGAGGGA-ATG--TG---GGCGAT-TA-TAACGAT----TCAATCGTGTTGTAT-------ATAGCGGCGGCCGCGGCTGAGTCGCTCT-GC----GCTAAT-A---CACA-CACACTC--GATA---CGTTAATGATCCTTCCGCAGGTTCCC-TACGGAAACCTTGTTACGACTTTTACTTCC [2086]

Y._polystictus CGCCTGTTTATCAAAAACATGTCTTTTTGAA-AATAATTTAAAGTCTGGTCTGCCCA-CTGATTTATAAATAATTAAAGGGCTGCAGTATATTGACTGTACAAAGGTAGCATAATCATTAGTCTTTTAATTGATGACTTGTATGAATGATCGGATAAAATATAAACTGTCTCTTAATAAATTTATAGAATTTAATTTTTTATTTAAAAAGTTAAAATAATTTTAAAAGACGAGAAGACCCTATAGAGTTTAATAA-TTTTATAAATTAAAATTATATGTATAAA-TTTAATTAAAAATTATAA-ATTTATTTTGTTGGGGTGATAAAAAAATTAATTAAACTTTTTTTAAAATTTA---------ACATAAATAATTGAATATTTGATCCAATTTT---TTTGATTATAAGAATAAATTACCTTAGGGATAACAGCGTAATTTTTTTTTTTAGTTCTTATAAAAAAAAAAGTTTGCGACCTCGATGTTGGATTAAGATAAAATTTAAATGCAAAAGTTTAAAATTTTGATCTGTTCGATCATTAAAATCTTA-CATGATCTGAGTTCAAACCGGAGATACCTCGACGTTATTCAGATTATCCAGATGCTTATATTTGTTGAAATATTATTTCTTCATTAGGATCTTATATTTCATTTTTAGCAATTATAATAATATTAATCATTATTTGAGAATCATTTATTTCTCAACGAATAATTTTATTTTCATTAAATATATCTTCTTCTATTGAATGACTTCAAAAATTCCCACCATCAGAACATTCTTATAATGAACTTCCTATTTTAAGAAATTTCTAATATGGCAGATAATATGTAATGGATTTAAACCCCATTTATAAAGGTTAATCCTTTTTTTAGAAATGGCAACATGAAATAATTTAAATTTACAAAATGGAGCATCTCCTTTAATAGAACAAATCATTTTTTTTCATGATCATACATTAATTATTTTAATTATAATTACAATTTTAGTAGGATATTTAATAATTAATTTATTCTTTAATAAATATACAAATCGATTTTTATTAGAAGGACAAATAATTGAATTAATTTGAACAATTTTACCAGCAATCACTTTAATTTTTATTGCTCTTCCATCTCTTCGTTTATTATATTTACTAGATGAATTAAATAATCCTTTAATTACATTAAAATCTATTGGTCACCAATGATATTGAAGTTATGAATATTCAGATTTTAATAATATTCAATTTGACTCATATATAATTCCAAGAAAAGAAATAAGAATTAATAATTTTCGATTATTAGATGTAGATAATCGTATTATTCTCCCTATAAATAATCAAATTCGAATTATAGTAACAGCAACAGATGTAATTCATTCTTGAACGGTTCCATCATTAGGAGTAAAAATTGATGCTAACCCAGGACGATTAAATCAAACTAATTTTTTTATTAATCGTCCTGGATTATTTTATGGTCAATGTTCTGAAATTTGTGGGGCAAATCATAGTTTTATACCTATTGTAATTGAAAGAATTTCAATTAATAATTTTATTAAATGAATTAATAATTATTCTTCATTAGATGACTGAAAGCAAGTAATGGTCTCGCTGCGTTCTTCATCGATGCGCGAGCCAAGTGATCCACCGTCCAGGGTAATGATTT----------------------------------------------------------------------------------------------G--GT-T-ATCTCTC----------GT---------TACGT---GC--------------GATA-----AGT---TG--AAAATGGAA-TAA-A-A--------AT-AAAAA----TAACGACAGACGAGCGCTCTCTCGCGCC---GTTG---A-G----A----ACG-A--GAGGCGC-TCGC-CATCACCGC-------CGAACAAGAAGAAC-TTCTTGTCGTTTATCATATATTTAACGTCCAC-CG-GCAGGTGCGACTCCGGGCGCGGATTTTA-GACCCATCGATGTCGT----------TTAGGACACC--------TGC-CGT--------------------G--------CGAC------------GTCCC--TCTCGTGTAGTCCCTCT-------CGTC----G---------AC--GGAAGGGCG-------------CCGC---G-CGCGT-CGA------------GNGNGC--ACA----C--GCCTTGCCTCGAGA--GAACAC-ACT-CGAGGGA-ATG--TG---GGCGAC-TA-TAACGAT----TCAATCGTGTTGTAT-------ATAGCGGCGGCCGCGGCTGAGTCGCTST-GC----GCTAAT-A---CACA-CACACTC--GATA---CGTTAATGATCCTTCCGCAGGTTCCCCTACGGAAACCTTGTTACGACTTTTACTTCC [2086]

Y._menkeni CGCCTGTTTATCAAAAACATGTCTTTTTGATTAATAATTTAAAGTCTGGTCTGCCCA-CTGATTTA-TTATAATTAAAGGGCTGCAGTATCTTGACTGTACAAAGGTAGCATAATCATTAGTCTTTTAATTGATGACTTGTATGAATGATCGGATAAGATATAAGCTGTCTCTTAATAAATTTATAGAATTTAATTTTTTATTTAAAAAGTTAAAATAATTTTAAAAGACGAGAAGACCCTATAGAGTTTAATAAATTTTATAAATTAAAATTTTATGTATAAA-TTTGATTAAAAATTATAT-ATTTATTTTATTGGGGTGATAAAAAAATTAATTAA-CTTTTTTTAAAA-TTA---------ACATAAATAAATGAGTATTTGATCCAATTTT-TTTTGGATTATAAGAATAAATTACCTTAGGGATAACAGCGTAATTTTTTTTTTTAGTTCTTATAAAAAAAAAAGTTTGCGACCTCGATGTTGGATTAAGATAAAATTTAAATGCAAAAGTTTAAAATTTTGATCTGTTCGATCATTAAAATCTTA-CATGATCTGAGTTCAAACCGGAGATACCTCGACGTTATTCAGATTACCCTAATGCTTATATTTGTTGAAATATTATTTCTTCTTTAGGATCTTATATTTCTTTTTTAGCAATTATAATAATATTAATTATTATTTGAGAATCCTTTATTTATCAACGAATAATTTTATTTTCATTAAATATACCTTCTTCTATTGAATGACTTCAAAAATTCCCACCATCAGAACATTCTTGTAATGAACTTCCTATTTTAAGAAACTTCTAATATGGCAGACTATATGTAATGGATTTAAACCCCATTTATAAAGGTTAATCCTTTTTTTAGAAATGGCAACATGAAACAATTTAAATTTACAAAATGGAGCATCTCCTTTAATAGAACAAATTATTTTTTTTCATGATCATACATTAATTATTTTAATTATAATTACAATTTTAGTAGGATATTTAATAATAAATTTATTATTTAATAAATACATTAATCGATTTTTATTAGAAGGTCAAATAATTGAATTAATTTGAACAATTTTACCAGCAATTACTTTAATTTTTATTGCTCTTCCTTCTCTTCGTTTATTATATTTATTAGATGAACTTAATAATCCTTTAATTACATTAAAATCTATTGGTCATCAATGATATTGAAGTTACGAATATTCAGATTTTAATAATATTCAGTTTGATTCTTATATAATTCCAAGTAAAGAAATAATAATTAATAATTTTCGATTACTAGATGTAGATAATCGTATTATTCTCCCTATAAATAATCAAATTCGTATTATAGTAACCGCAACTGATGTAATTCATTCTTGAACAATTCCTTCACTAGGAGTTAAAATTGATGCTAATCCAGGACGTTTAAATCAAACAAATTTTTTTATTAATCGTCCTGGTTTATTTTATGGTCAATGCTCTGAAATTTGTGGAGCAAATCATAGTTTTATACCAATTGTAATTGAAAGAATTTCAATTAATAATTTTATTAAATGAATTAATAATTATTCTTCATTAGATGACTGAAAGCAAGTAATGGTCTCGCTGCGTTCTTCATCGATGCGCGAGCCAAGTGATCCACCGTCCAGGGTAATGATTT----------------------------------------------------------------------------------------------G--GT-T-ATCTCTC----------GT---------TACGT---GC--------------GATA-----AGT---TG--AAAAT-GAAATAA-A-A--------AT-AAAAA----TAACGACAGACGAGCGCTCTCTCGCGCC---GTTG---A-G----A----ACG-A--GAGGCGC-TCGC-CATCACCGC-------CGAACAAGAAGAAC-TTCTTGTCGTTTATTATATATTTAACGTCCAC-CG-GCAGGTGCGACTCCGGGCGCGGATTTTA-GACCCATCGATGTCGT----------TTAGGACACC--------TGC-CGT--------------------G--------CGAC------------GTCCC--TCTCGTGTAGTCCCTCT-------CGTC----G---------AC--GGAGG--CG--------------CGCCGCG-CGCGT-CGA------------GTGTGC--ACA----C--GCCTTGCCTCGAGA--GAACAC-ACT-CGAGGGA-ATG--TG---GGCGAT-TA-TAACGAT----TCAATCGTGTTGTAT-------ATAGCGGCGGCCGCGGCTGAGTCGCTGCTGC----GCTAAT-A---CACA-CACT--C--GATA---CGTTAATGATCCTTCCGCAGGTTCCCCTACGGAAACCTTGTTACGACTTTTACTTCC [2086]

Euhyponomeutoides_trachydeltus CGCCTGTTTATCAAAAACATGTCTTTTTGAA-AATAATTTAAAGTCTAGTCTGCCCA-CTGAT----AAATAATTAAAGGGCTGCAGTATATTGACTGTACAAAGGTAGCATAATAATTAGTCTTTTAATTGATGACTTGTATGAATGATTGGATAAGATATAAGCTGTCTCTTAATAAATTTATAGAATTTAATTTTTTATTTAAAAAGTTAAAATGATTTTAAAAGACGAGAAGACCCTATAGAGTTTAATAT-TTAAATGAATTAAAATTATTTTTAAAAT-TTAAATTAAAATTTATTTTATTTATTTTATTGGGGTGATAGAAAAATTAAATAAACCTTTTTTTAAATATA-------AACCATAAATAAATGAATATATGATCCAAAATTTATTTTGATTAAAAGATTAAATTACCTTAGGGATAACAGCGTAATTTTTTTTTTTAGTTCTTATAAGAAAAAAAGTTTGCGACCTCGATGTTGGATTAAGATAAAATTTAAATGCAAAAGTTTAAAATTTTGATCTGTTCGATCATTAAAATCTTA-CATGATCTGAGTTCAAACCGGAGATACCTCGACGTTATTCAGACTATCCCGATGCATATATTTGTTGAAATATTATTTCATCCCTAGGATCTTATATTTCATTTTTAGCAATCATAAAAATATTAATTATTATTTGAGAATCTTTTATTTCACAACGAATAATTTTATTTTCAATAAACATATCCTCCTCAATTGAATGATTACAAAAATTCCCCCCATCAGAACATTCGTATAATGAACTTCCTATTTTAAGAAATTTCTAATATGGCAGATTATATGTAATGGATTTAAACCCCATTTATAAAGGTTTATCCTTTTTTTAGAAATGGCAACATGAACTAATTTAAATTTTCAAAATGGAGCATCTCCTTTAATAGAACAAATTATTTTTTTTCATGATCTTACATTAATTATTTTAATTATAATTACTATTCTAGTTGGGTATTTAATAATTAATTTATTTTTTAATAAATACACAAATCGTTTTTTATTAGAAGGTCAAATAATTGAACTAATTTGAACAATTTTACCTGCAATTACTTTAATTTTTATTGCTTTACCTTCGCTTCGTTTATTATACTTATTAGATGAACTTAATAATCCTTTAATTACATTAAAATCTATTGGACATCAATGATATTGAAGTTATGAATACTCAGATTTTAATAATATTCAATTTGATTCATATATAATCCCAAGAAAAGAATTAAGAATTAATAATTTCCGATTACTAGATGTTGATAATCGTATTATTTTACCTATAAATAATCAAATTCGAATTATAGTAACTGCAACAGATGTTATTCATTCTTGAACTATTCCCTCATTAGGGGTTAAAGTTGATGCTAATCCAGGTCGATTAAACCAAACAAACTTTTTTATTAATCGACCTGGATTATTTTATGGTCAATGCTCAGAAATTTGCGGAGCAAATCATAGATTTATACCTATTGTAATTGAAAGAATCTCAATTAATAATTTTATTAAATGAATTAATAATTATTCCTCATTAGATGACTGAAAGCAAGTAATGGTCTCGCTGCGTTCTTCATCGATGCGCGAGCCAAGTGATCCACCGTCCAGGGTAATGATTT----------------------------------------------------------------------------------------------GGCGCATTACCAC-CG---------GTT-------GTTCTTTGAGC--------------GATATC---AAT---T-----AAT--AA--AATAGATT-------T--G-CA----TAACGACAAACGAGCGCTCTC--CCGCT----TCT---A------AT---ACG-A--GAGGCGC-TCGC-CATCACCGC-------------TGT------TTCT----GTGCGT---AT--TTAACGTCC-C-CGCGCGCGTGCGACTCCGGGCGCGGATTTCG-TACCCATCGATGTCGT----------TTAA-ACACG--------TGCACGT--------------------G--------CGAC------------GTCTC--TCCC--------C---T-------CCCC--------------CC--CCCTCTTCG-------------CCGATCGGTAACG--CTT------------ACGCGC--ACA----CGGATC-TTCGTCGAGATAGAGAGAGAGAGAGAGAGAGATAGATATGTGTGGATATAGTAACGATG---TAA-------------------AT--TAACGGCC---------TTGC----------GC-GATCA---CGCAGCACTG-CTAAATAT-ACGTTAATGATCCTTCCGCAGGTTCCCCTACGGAAACCTTGTTACGACTTTTACTTCC [2052]

Y._multipunctellus -----------------------------------------------------------------------------------------------------------------------------------------------------------------------------------------------------------------------------------------------------------------------------------------------------------------------------------------------------------------------------------------------------------------------------------------------------------------------------------------------------------------------------------------------------------------------------------------------------------------------------------------------------------------------------------------------------------------------------------------------------------------------------------------------------------------------------------------------------------------------------------------------------------------------------------------------GGAGCATCTCCTTTAATAGAACAAATCATTTTTTTTCATGATCATACATTAATTATTTTAATTATAATTACAATTTTAGTAGGTTATTTAATAATTAATTTATTATTTAATAAATATATTAATCGATTTTTATTAGAAGAACAAATAATCGAATTAATTTGAACAATTTTACCAGCTATTACTTTAAATTTTATTGCTTTACCTTCACTTCGTTTATTATATTTATTAGATGAACTTAATAATCCTTTAATTACATTAAAATCTATTGGACATCAATGATATTGAAGTTATGAATATTCAGATTTTAATAATATTCAATTTGATTCTTATATAATTCCAAGAAAAGATATAATAATTAATAATTTTCGATTACTAGATGTAGATAATCGAATTATCCTTCCTTTAAATAATCAAATTCGTATTATAGTAACTGCAACAGATGTAATTCATTCTTGAACAATTCCATCATTAGGAGTAAAAATTGATGCTAATCCAGGACGATTAAATCAAACAAATTTTTTTATTAATCGTCCTGGATTATTTTATGGACAATGTTCTGAAATTTGTGGAGCTAATCATAGTTTTATACCTATTGTAATTGAAAGAATTTCAATTAATAATTTTATTAAATGAATTAATAATTATATTTCATTAGATGACTGAAAGCAAGTAATGGTCTCGCTGCGTTCTTCATCGATGCGCGAGCCAAGTGATCCACCGTCCAGGGTAATGATTT------------------------------------------------------------------------------------------------CGCCTCATCAC-CATGT--AATTAGT-----T-ATAT-T---G----------------ATGCGTCACGTAAATT--GAAGTA--AAAAA-A-A--------AA----AA----TAACGACAAACGAGCGCTCTC--ACGCT---GTC----A------A----ACG-CC-GAGGCGC-TCGC-CATCACCGC-------------AGT-G----TT-TTT-TGTGTGT---ATATTTAACGTCCACT-G-GCACGTGCGACTCCGGGCGCGGATTTTA-GACCCATCGATGTCGT----------TTA-GACGCG--------TGC-CGT--------------------G--------CGAC------------GTCTC--TCT-------------T-------CGCC----G---------AT?-CGC-G--CG--------------CGC-GCG-CGCT--CGCTCTTCAA-CG--GCGCGC--GCACG--G--ATC-TACGTCGAGA--GAA------TGCGAGGGA--TG--TC---AT-AAT-TA-TAACGATG---T-A-------------------AT-----CGT----------GT-------------GA---T-A---TACA-CG----C--GTTA---CGTTAATGATCCTTCCGCAGGTTCCC-TACGGAAACCTTGTTACGACTTTTACTTCC [1133]

;

END;

BEGIN ASSUMPTIONS;

EXSET 16S = 577-2454;

EXSET CO = 1-576 1592-2454;

EXSET ITS = 1-1591;

EXSET mito = 1592-2454;

EXSET wo16S = 1-576;

EXSET woCO = 577-1591;

EXSET woITS = 1592-2454;

wtset * 16Ssecstr = 1: 1 2 5-9 15-19 22-26 41-45 48-51 53-55

59-63 65 72 75-88 95-101 121-126 132-137 148-151 155

156 198-202 211-215 239-242 244-250 252-254 258-267 296-306 308-310 313-316 322-331

342-347 367-369 376-378 390-392 401-403 415-419 422-427

436-438 440-447 451 452 460 461 465-471 474-477 488-498

501-510 515-525 527-531 537-541 547-551 555 558-561 563,

2: 3 4 10-14 20 21 27-40 46 47 52 56-58 64 66-71 73 74

89-94 102-105 106-120 127-131 138-147 152-154 157-197 203-210 216-238 243 251 255-257 268-295

307 311 312 317-321 332-341 348-366 370-375 379-389 393-400

404-414 420 421 428-435 439 448-450 453-459 462-464 472

473 478-487 499 500 511-514 526 532-536 542-546 552-554

556 557 562 564-2454;

END;

BEGIN SETS;

TAXSET 16S = 17-22 24-27 29-35 37-40;

TAXSET CO = 17-34 37-41;

TAXSET ITS = 17-23 25-41;

TAXSET mito = 17-35 37-41;

TAXSET TOTAL = 17-41;

CHARSET 16S = 1-576; CHARSET CO = 577-1591; CHARSET ITS = 1592-2454;

CHARPARTITION ILD = 1: 16S, 2: CO, 3: ITS;

CHARPARTITION MITO = 1: 16S, 2: CO;

CHARPARTITION 16S_ITS = 1: 16S, 2: ITS;

CHARPARTITION CO_ITS = 1: CO, 2: ITS;

END;

BEGIN PAUP;out Euhyponomeutoides_trachydeltus Xyrosaris_lichneuta;assume wtset=16Ssecstr;

[! Likelihood settings for 16S ]

Lset Base=(0.400374 0.087699 0.125822) Nst=6 Rmat=(0.771276 5.179894 3.075888 0.000017 1.511002) Rates=gamma Shape= 0.167785;

[! Likelihood settings for CO ]

Lset Base=(0.361944 0.128758 0.102309) Nst=6 Rmat=(0.730018 4.172129 3.206646 0.160432 14.885474) Rates=gamma Shape=0.124585;

[! Likelihood settings for ITS ]

Lset Base=(0.236345 0.267373 0.245185) Nst=6 Rmat=(0.958565 3.423088 1.357795 1.151147 2.901429) Rates=gamma Shape=0.282890;

[! Likelihood settings for 16S and CO in the mitochondrial analysis ]

[16S:]

Lset Base =(0.388365 0.090883 0.130735) Nst=6 Rmat=(0.690012 6.176052 3.800534 0.000017 2.406864) Rates=gamma Shape=0.146957 ;

[CO:]

Lset Base=(0.361944 0.128758 0.102309) Nst=6 Rmat=(0.595418 3.601996 2.753682 0.104941 13.625033) Rates=gamma Shape=0.121554;

[! Likelihood settings for 16S, CO and ITS in the total-evidence analysis ]

[16S:]

Lset Base =(0.400373 0.087700 0.125823) Nst=6 Rmat=(0.690012 6.176052 3.800534 0.000017 2.406864) Rates=gamma Shape=0.155609 ;

[CO:]

Lset Base=(0.361944 0.128758 0.102309) Nst=6 Rmat=(0.595418 3.601996 2.753682 0.104941 13.625033) Rates=gamma Shape=0.122284;

[ITS:]

Lset Base=(0.236912 0.265920 0.243355) Nst=6 Rmat=(2.890976 8.396543 3.674521 3.151766 8.540486) Rates=gamma Shape=0.322785;

]

END;

[[The specimens of Y. cagnagellus and padellus are numbered as in Table 1 in the full text]]
