# Supplementary material for: Molecular Phylogeny of the Small Ermine Moth Genus Yponomeuta (Lepidoptera, Yponomeutidae) in the Palaearctic
Source: PLoS One. 2010 Mar 29;5(3):e9933. doi: 10.1371/journal.pone.0009933 (PMC2847947; doi:10.1371/journal.pone.0009933)
Supplement: Text S2 — Mitochondrial host Lagrange results. Evolution of host range based on mitochondrial Bayesian analysis tree. (0.05 MB DOC) [file pone.0009933.s007.doc]

B. Evolution of host range based on total-evidence Bayesian analysis tree.

EU: western Palaearctic; C: Celastraceae; Cr: Crassulaceae; R: Rosaceae; S: Salicaceae

lagrange: likelihood analysis of geographic range evolution

Version 2 released February 2008

This is development snapshot 20091004

Authors: Richard Ree [rree@fieldmuseum.org](mailto:rree@fieldmuseum.org), Stephen Smith <sasmith@nescent.org>

http://lagrange.googlecode.com

Newick tree with interior nodes labeled:

((((((((((cag:1.01538461538,mali:1.01538461538)I:4.06153846154,((((pad:1.01538461538,maha:1.01538461538)II:1.01538461538,gris:2.03076923077)III:1.01538461538,ror:3.04615384615)IV:1.01538461538,gig:4.06153846154)V:1.01538461538)VI:1.01538461538,(irr:1.01538461538,evon:1.01538461538)VII:5.07692307692)VIII:1.01538461538,(menk:1.01538461538,mult:1.01538461538)IX:6.09230769231)X:1.01538461538,((((pstc:1.01538461538,pstg:1.01538461538)XI:1.01538461538,soc:2.03076923077)XII:1.01538461538,toky:3.04615384615)XIII:1.01538461538,spod:4.06153846154)XIV:4.06153846154)XV:1.01538461538,(meg:1.01538461538,euri:1.01538461538)XVI:8.12307692308)XVII:1.01538461538,plum:10.1538461538)XVIII:1.01538461538,((sedJ:1.01538461538,sed:1.01538461538)XIX:1.01538461538,yana:2.03076923077)XX:9.13846153846)XXI:1.01538461538,Euhyp:12.1846153846)XXII:1.01538461538,Xyro:13.2)XXIII:0.0;

Cladogram (branch lengths not to scale):

----------+ [C] Y. cagnagellus

---------I+

: ----------+ [R] Y. malinellus

:

: ----+ [R] Y. padellus

--VI+ --II+

: : -III+ ----+ [R] Y. mahalabellus

: : : :

: : --IV+ --------+ [C] Y. griseatus

: : : :

VIII+ ---V+ ------------+ [S] Y. rorrellus

: : :

: : ----------------+ [S] Y. gigas

: :

---X+ : ------------+ [C] Y. irrorellus

: : ---------VII+

: : ------------+ [R] Y. evonymellus

: :

: : --------------+ [C] Y. menkeni

: ------------IX+

: --------------+ [C] Y. multipunctellus

--XV+

: : -------+ [C] Y. polystictus

: : -----XI+

: : ---XII+ -------+ [C] Y. polystigmellus

: : : :

: : --XIII+ --------------+ [C] Y. sociatus

XVII+ : : :

: : ---XIV+ --------------------+ [C] Y. tokyonellus

: : :

: : --------------------------+ [C] Y. spodocrossus

XVIII+ :

: : : ------------------+ [C] Y. meguronius

: : ---------------XVI+

: : ------------------+ [C] Y. eurinellus

-XXI+ :

: : ----------------------------------------+ [C] Y. plumbellus

: :

: : ---------------+ [Z] Y. sedellus J

XXII+ : ------------XIX+

: : ------------XX+ ---------------+ [Z] Y. sedellus

: : :

XXIII+ : ------------------------------+ [C] Y. yanagawanus

: :

: ------------------------------------------------+ [C] Euhyponomeutoides trachydeltus

:

----------------------------------------------------+ [C] Xyrosaris lichneuta

Global ML at root node:

-lnL = 30.98

dispersal = 0.0152

extinction = 4.285e-09

Ancestral range subdivision/inheritance scenarios ('splits') at

internal nodes.

* Split format: [left|right], where 'left' and 'right' are the ranges

inherited by each descendant branch (on the printed tree, 'left' is

the upper branch, and 'right' the lower branch).

* Only splits within 2 log-likelihood units of the maximum for each

node are shown. 'Rel.Prob' is the relative probability (fraction of

the global likelihood) of a split.

At node XXIII:

split lnL Rel.Prob

[C|C] -31.03 0.9525

At node XXII:

split lnL Rel.Prob

[C|C] -31.03 0.9483

At node XXI:

split lnL Rel.Prob

[C|C] -31.06 0.9244

At node XVIII:

split lnL Rel.Prob

[C|C] -30.98 0.9998

At node XVII:

split lnL Rel.Prob

[C|C] -30.98 0.9991

At node XV:

split lnL Rel.Prob

[C|C] -30.98 0.995

At node X:

split lnL Rel.Prob

[C|C] -31.01 0.9719

At node VIII:

split lnL Rel.Prob

[C|C] -31.13 0.8549

At node VI:

split lnL Rel.Prob

[C|C] -31.35 0.6859

[C|CS] -32.91 0.1443

At node I:

split lnL Rel.Prob

[C|R] -30.98 1

At node V:

split lnL Rel.Prob

[CS|S] -31.12 0.8686

At node IV:

split lnL Rel.Prob

[C|S] -31.09 0.8946

At node III:

split lnL Rel.Prob

[R|C] -30.98 1

At node II:

split lnL Rel.Prob

[R|R] -30.98 1

At node VII:

split lnL Rel.Prob

[C|R] -30.98 1

At node IX:

split lnL Rel.Prob

[C|C] -30.98 1

At node XIV:

split lnL Rel.Prob

[C|C] -30.98 1

At node XIII:

split lnL Rel.Prob

[C|C] -30.98 1

At node XII:

split lnL Rel.Prob

[C|C] -30.98 1

At node XI:

split lnL Rel.Prob

[C|C] -30.98 1

At node XVI:

split lnL Rel.Prob

[C|C] -30.98 1

At node XX:

split lnL Rel.Prob

[Z|C] -30.98 1

At node XIX:

split lnL Rel.Prob

[Z|Z] -30.98 1
