# Supplementary material for: Molecular Phylogeny of the Small Ermine Moth Genus Yponomeuta (Lepidoptera, Yponomeutidae) in the Palaearctic
Source: PLoS One. 2010 Mar 29;5(3):e9933. doi: 10.1371/journal.pone.0009933 (PMC2847947; doi:10.1371/journal.pone.0009933)
Supplement: Text S3 — Total-evidence host Lagrange results. Evolution of host range based on total-evidence Bayesian analysis tree. (0.05 MB DOC) [file pone.0009933.s008.doc]

B. Evolution of host range based on total-evidence Bayesian analysis tree.

EU: western Palaearctic; C: Celastraceae; Cr: Crassulaceae; R: Rosaceae; S: Salicaceae

lagrange: likelihood analysis of geographic range evolution

Version 2 released February 2008

This is development snapshot 20091004

Authors: Richard Ree rree@fieldmuseum.org, Stephen Smith <sasmith@nescent.org>

http://lagrange.googlecode.com

Newick tree with interior nodes labeled:

((((((sed:1.09090909091,plum:1.09090909091)I:1.09090909091,sedJ:2.18181818182)II:1.09090909091,yana:3.27272727273)III:6.54545454545,(((mult:1.09090909091,gris:1.09090909091)IV:6.54545454545,(((irr:1.09090909091,evon:1.09090909091)V:4.36363636364,((cag:1.09090909091,mali:1.09090909091)VI:3.27272727273,(((ror:1.09090909091,gig:1.09090909091)VII:1.09090909091,maha:2.18181818182)VIII:1.09090909091,pad:3.27272727273)IX:1.09090909091)X:1.09090909091)XI:1.09090909091,(((((spod:1.09090909091,soc:1.09090909091)XII:1.09090909091,pstg:2.18181818182)XIII:1.09090909091,pstc:3.27272727273)XIV:1.09090909091,toky:4.36363636364)XV:1.09090909091,(kana:1.09090909091,menk:1.09090909091)XVI:4.36363636364)XVII:1.09090909091)XVIII:1.09090909091)XIX:1.09090909091,(meg:1.09090909091,euri:1.09090909091)XX:7.63636363636)XXI:1.09090909091)XXII:1.09090909091,Euhyp:10.9090909091)XIII:1.09090909091,Xyro:12.0)XIV:0.0;

Cladogram (branch lengths not to scale):

------------+ [Cr] Y. sedellus

----------I+

---------II+ ------------+ [C] Y. plumbellus

: :

--------III+ -----------------------+ [Cr] Y. sedellus J

: :

: ----------------------------------+ [C] Y. yanagawanus

:

: ------------------+ [C] Y. multipunctellus

: ---------------IV+

: : ------------------+ [C] Y. griseatus

: :

: : -------------+ [C] Y. irrorellus

: : -----------V+

: : : -------------+ [R] Y. evonymellus

: : :

: : ---XI+ ----------+ [C] Y. cagnagellus

-XXII+ --XIX+ : : --------VI+

: : : : : : : ----------+ [R] Y. malinellus

: : : : : : :

: : : : : ----X+ -----+ [S] Y. rorrellus

: : : : : : --VII+

: : : : : : -VIII+ -----+ [S] Y. gigas

: : : : : : : :

: : : : : ---IX+ ----------+ [R] Y. mahalebellus

: : : : : :

: : : XVIII+ ---------------+ [R] Y. padellus

: : : :

: : : : -----+ [C] Y. spodocrossus

: : : : --XII+

: --XXI+ : -XIII+ -----+ [C] Y. sociatus

-XIII+ : : : :

: : : : --XIV+ ----------+ [C] Y. polystigmellus

: : : : : :

: : : : ---XV+ ---------------+ [C] Y. polystictus

: : : : : :

: : : -XVII+ --------------------+ [C] Y. tokyonellus

: : : :

: : : : -------------+ [C] Y. kanaiellus

XIV+ : : ---------XVI+

: : : -------------+ [C] Y. menkeni

: : :

: : : --------------------+ [C] Y. meguronis

: : ------------------XX+

: : --------------------+ [C] Y. eurinellus

: :

: --------------------------------------------------+ [C] Euhyponomeutoides

: trachydeltus

-------------------------------------------------------+ [C] Xyrosaris lichneuta

Global ML at root node:

-lnL = 28.4

dispersal = 0.01357

extinction = 4.285e-09

Ancestral range subdivision/inheritance scenarios ('splits') at

internal nodes.

* Split format: [left|right], where 'left' and 'right' are the ranges

inherited by each descendant branch (on the printed tree, 'left' is

the upper branch, and 'right' the lower branch).

* Only splits within 2 log-likelihood units of the maximum for each

node are shown. 'Rel.Prob' is the relative probability (fraction of

the global likelihood) of a split.

At node XIV:

split lnL Rel.Prob

[C|C] -28.44 0.9609

At node XIII:

split lnL Rel.Prob

[C|C] -28.45 0.9576

At node XXII:

split lnL Rel.Prob

[C|C] -28.47 0.9386

At node III:

split lnL Rel.Prob

[C+Cr|C] -29.03 0.5366

[C|C] -29.29 0.4116

At node II:

split lnL Rel.Prob

[C+Cr|Cr] -28.46 0.9424

At node I:

split lnL Rel.Prob

[Cr|C] -28.4 1

At node XXI:

split lnL Rel.Prob

[C|C] -28.41 0.9951

At node XIX:

split lnL Rel.Prob

[C|C] -28.43 0.9769

At node IV:

split lnL Rel.Prob

[C|C] -28.4 1

At node XVIII:

split lnL Rel.Prob

[C|C] -28.54 0.8744

At node XI:

split lnL Rel.Prob

[C|C] -29.34 0.3907

[C+R|R] -29.37 0.3789

[R|C+R] -30.9 0.08233

[C|C+R] -30.9 0.08233

At node V:

split lnL Rel.Prob

[C|R] -28.4 1

At node X:

split lnL Rel.Prob

[C+R|R] -28.95 0.5766

[R|R] -29.36 0.3855

At node VI:

split lnL Rel.Prob

[C|R] -28.4 1

At node IX:

split lnL Rel.Prob

[R|R] -28.58 0.838

[R+S|R] -30.24 0.1597

At node VIII:

split lnL Rel.Prob

[S|R] -28.4 1

At node VII:

split lnL Rel.Prob

[S|S] -28.4 1

At node XVII:

split lnL Rel.Prob

[C|C] -28.4 1

At node XV:

split lnL Rel.Prob

[C|C] -28.4 1

At node XIV:

split lnL Rel.Prob

[C|C] -28.4 1

At node XIII:

split lnL Rel.Prob

[C|C] -28.4 1

At node XII:

split lnL Rel.Prob

[C|C] -28.4 1

At node XVI:

split lnL Rel.Prob

[C|C] -28.4 1

At node XX:

split lnL Rel.Prob

[C|C] -28.4 1
