# Supplementary material for: Molecular Phylogeny of the Small Ermine Moth Genus Yponomeuta (Lepidoptera, Yponomeutidae) in the Palaearctic
Source: PLoS One. 2010 Mar 29;5(3):e9933. doi: 10.1371/journal.pone.0009933 (PMC2847947; doi:10.1371/journal.pone.0009933)
Supplement: Text S4 — Mitochondrial distribution Lagrange results. Evolution of biogeographical range based on mitochondrial Bayesian analysis tree. (0.05 MB DOC) [file pone.0009933.s009.doc]

C. Evolution of biogeographical range based on mitochondrial Bayesian analysis tree.

EU: western Palaearctic; AS: Far East; NA: North America; AF: Africa (Canary Islands)

lagrange: likelihood analysis of geographic range evolution

Version 2 released February 2008

This is development snapshot 20091004

Authors: Richard Ree <rree@fieldmuseum.org>

Stephen Smith <sasmith@nescent.org>

http://lagrange.googlecode.com

Newick tree with interior nodes labeled:

((((((((((cag:1.01538461538,mali:1.01538461538)I:4.06153846154,((((pad:1.01538461538,maha:1.01538461538)II:1.01538461538,gris:2.03076923077)III:1.01538461538,ror:3.04615384615)IV:1.01538461538,gig:4.06153846154)V:1.01538461538)VI:1.01538461538,(irr:1.01538461538,evon:1.01538461538)VII:5.07692307692)VIII:1.01538461538,(menk:1.01538461538,mult:1.01538461538)IX:6.09230769231)X:1.01538461538,((((pstc:1.01538461538,pstg:1.01538461538)XI:1.01538461538,soc:2.03076923077)XII:1.01538461538,toky:3.04615384615)XIII:1.01538461538,spod:4.06153846154)XIV:4.06153846154)XV:1.01538461538,(meg:1.01538461538,euri:1.01538461538)XVI:8.12307692308)XVII:1.01538461538,plum:10.1538461538)XVIII:1.01538461538,((sedJ:1.01538461538,sed:1.01538461538)XIX:1.01538461538,yana:2.03076923077)XX:9.13846153846)XXI:1.01538461538,Euhyp:12.1846153846)XXII:1.01538461538,Xyro:13.2)XXIII:0.0;

Cladogram (branch lengths not to scale):

----------+ [EU] Y. cagnagellus

---------I+

: ----------+ [EU] Y. malinellus

:

: ----+ [EU] Y. padellus

--VI+ --II+

: : -III+ ----+ [EU] Y. mahalabellus

: : : :

: : --IV+ --------+ [AS] Y. griseatus

: : : :

VIII+ ---V+ ------------+ [EU] Y. rorrellus

: : :

: : ----------------+ [AF] Y. gigas

: :

---X+ : ------------+ [EU] Y. irrorellus

: : ---------VII+

: : ------------+ [EU+AS] Y. evonymellus

: :

: : --------------+ [AS] Y. menkeni

: ------------IX+

: --------------+ [NA] Y. multipunctellus

--XV+

: : -------+ [AS] Y. polystictus

: : -----XI+

: : ---XII+ -------+ [AS] Y. polystigmellus

: : : :

: : --XIII+ --------------+ [AS] Y. sociatus

XVII+ : : :

: : ---XIV+ --------------------+ [AS] Y. tokyonellus

: : :

: : --------------------------+ [AS] Y. spodocrossus

XVIII+ :

: : : ------------------+ [AS] Y. meguronius

: : ---------------XVI+

: : ------------------+ [AS] Y. eurinellus

-XXI+ :

: : ----------------------------------------+ [EU] Y. plumbellus

: :

: : ---------------+ [AS] Y. sedellus J

XXII+ : ------------XIX+

: : ------------XX+ ---------------+ [EU] Y. sedellus

: : :

XXIII+ : ------------------------------+ [AS] Y. yanagawanus

: :

: ------------------------------------------------+ [AS] Euhyponomeutoides

: trachydeltus

----------------------------------------------------+ [AS] Xyrosaris lichneuta

Global ML at root node:

-lnL = 37.2

dispersal = 0.02115

extinction = 0.01222

Ancestral range subdivision/inheritance scenarios ('splits') at

internal nodes.

* Split format: [left|right], where 'left' and 'right' are the ranges

inherited by each descendant branch (on the printed tree, 'left' is

the upper branch, and 'right' the lower branch).

* Only splits within 2 log-likelihood units of the maximum for each

node are shown. 'Rel.Prob' is the relative probability (fraction of

the global likelihood) of a split.

At node XXIII:

split lnL Rel.Prob

[AS|AS] -37.48 0.7577

[EU+AS|AS] -39.29 0.1239

At node XXII:

split lnL Rel.Prob

[AS|AS] -37.38 0.8368

[EU+AS|AS] -39.3 0.1226

At node XXI:

split lnL Rel.Prob

[AS|AS] -37.46 0.7693

At node XVIII:

split lnL Rel.Prob

[AS|AS] -37.73 0.585

[AS|EU] -38.6 0.2457

[EU+AS|EU] -39.53 0.09707

At node XVII:

split lnL Rel.Prob

[AS|AS] -37.34 0.871

At node XV:

split lnL Rel.Prob

[AS|AS] -37.37 0.8387

[EU+AS|AS] -39.12 0.1462

At node X:

split lnL Rel.Prob

[AS|AS] -37.68 0.617

[EU+AS|AS] -38.95 0.1736

[EU|AS] -38.99 0.1663

At node VIII:

split lnL Rel.Prob

[EU|EU+AS] -37.62 0.6566

[EU|EU] -38.94 0.1755

At node VI:

split lnL Rel.Prob

[EU|EU] -37.42 0.8043

At node I:

split lnL Rel.Prob

[EU|EU] -37.2 0.9972

At node V:

split lnL Rel.Prob

[EU|AF] -37.78 0.5613

[EU|EU] -38.43 0.2902

At node IV:

split lnL Rel.Prob

[EU|EU] -37.45 0.7753

[EU+AS|EU] -38.75 0.2124

At node III:

split lnL Rel.Prob

[EU|AS] -37.35 0.8612

At node II:

split lnL Rel.Prob

[EU|EU] -37.2 0.9957

At node VII:

split lnL Rel.Prob

[EU|EU+AS] -37.33 0.873

At node IX:

split lnL Rel.Prob

[AS|NA] -37.23 0.9694

At node XIV:

split lnL Rel.Prob

[AS|AS] -37.2 0.9954

At node XIII:

split lnL Rel.Prob

[AS|AS] -37.2 0.9987

At node XII:

split lnL Rel.Prob

[AS|AS] -37.2 0.9993

At node XI:

split lnL Rel.Prob

[AS|AS] -37.2 0.9996

At node XVI:

split lnL Rel.Prob

[AS|AS] -37.2 0.9966

At node XX:

split lnL Rel.Prob

[EU+AS|AS] -37.58 0.6816

[AS|AS] -38.4 0.3015

At node XIX:

split lnL Rel.Prob

[AS|EU] -37.23 0.9641
