# Supplementary material for: Molecular Phylogeny of the Small Ermine Moth Genus Yponomeuta (Lepidoptera, Yponomeutidae) in the Palaearctic
Source: PLoS One. 2010 Mar 29;5(3):e9933. doi: 10.1371/journal.pone.0009933 (PMC2847947; doi:10.1371/journal.pone.0009933)
Supplement: Text S5 — Total-evidence distribution Lagrange results. Evolution of biogeographical range based on total-evidence Bayesian analysis tree. (0.05 MB DOC) [file pone.0009933.s010.doc]

D. Evolution of biogeographical range based on total-evidence Bayesian analysis tree.

EU: western Palaearctic; AS: Far East; NA: North America; AF: Africa (Canary Islands)

lagrange: likelihood analysis of geographic range evolution

Version 2 released February 2008

This is development snapshot 20091004

Authors: Richard Ree [rree@fieldmuseum.org](mailto:rree@fieldmuseum.org)

Stephen Smith <sasmith@nescent.org>

http://lagrange.googlecode.com

Newick tree with interior nodes labeled:

((((((sed:1.2,plum:1.2)I:1.2,sedJ:2.4)II:1.2,yana:3.6)III:7.2,(((mult:1.2,gris:1.2)IV:7.2,(((irr:1.2,evon:1.2)V:4.8,((cag:1.2,mali:1.2)VI:3.6,(((ror:1.2,gig:1.2)VII:1.2,maha:2.4)VIII:1.2,pad:3.6)IX:1.2)X:1.2)XI:1.2,(((((spod:1.2,soc:1.2)XII:1.2,pstg:2.4)XIII:1.2,pstc:3.6)XIV:1.2,toky:4.8)XV:1.2,(kana:1.2,menk:1.2)XVI:4.8)XVII:1.2)XVIII:1.2)XIX:1.2,(meg:1.2,euri:1.2)XX:8.4)XXI:1.2)XXII:1.2,Euhyp:12.0)XIII:1.2,Xyro:13.2)XIV:0.0;

Cladogram (branch lengths not to scale):

------------+ [EU] Y. sedellus

----------I+

---------II+ ------------+ [EU] Y. plumbellus

: :

--------III+ -----------------------+ [AS] Y. sedellus J

: :

: ----------------------------------+ [AS] Y. yanagawanus

:

: ------------------+ [NA] Y. multipunctellus

: ---------------IV+

: : ------------------+ [AS] Y. griseatus

: :

: : -------------+ [EU] Y. irrorellus

: : -----------V+

: : : -------------+ [EU+AS] Y. evonymellus

: : :

: : ---XI+ ----------+ [EU] Y. cagnagellus

-XXII+ --XIX+ : : --------VI+

: : : : : : : ----------+ [EU] Y. malinellus

: : : : : : :

: : : : : ----X+ -----+ [EU] Y. rorrellus

: : : : : : --VII+

: : : : : : -VIII+ -----+ [AF] Y. gigas

: : : : : : : :

: : : : : ---IX+ ----------+ [EU] Y. mahalebellus

: : : : : :

: : : XVIII+ ---------------+ [EU] Y. padellus

: : : :

: : : : -----+ [AS] Y. spodocrossus

: : : : --XII+

: --XXI+ : -XIII+ -----+ [AS] Y. sociatus

-XIII+ : : : :

: : : : --XIV+ ----------+ [AS] Y. polystigmellus

: : : : : :

: : : : ---XV+ ---------------+ [AS] Y. polystictus

: : : : : :

: : : -XVII+ --------------------+ [AS] Y. tokyonellus

: : : :

: : : : -------------+ [AS] Y. kanaiellus

XIV+ : : ---------XVI+

: : : -------------+ [AS] Y. menkeni

: : :

: : : --------------------+ [AS] Y. meguronis

: : ------------------XX+

: : --------------------+ [AS] Y. eurinellus

: :

: --------------------------------------------------+ [AS] Euhyponomeutoides

: trachydeltus

-------------------------------------------------------+ [AS] Xyrosaris lichneuta

Global ML at root node:

-lnL = 26.71

dispersal = 0.01269

extinction = 4.285e-09

Ancestral range subdivision/inheritance scenarios ('splits') at

internal nodes.

* Split format: [left|right], where 'left' and 'right' are the ranges

inherited by each descendant branch (on the printed tree, 'left' is

the upper branch, and 'right' the lower branch).

* Only splits within 2 log-likelihood units of the maximum for each

node are shown. 'Rel.Prob' is the relative probability (fraction of

the global likelihood) of a split.

At node XIV:

split lnL Rel.Prob

[AS|AS] -26.75 0.9585

At node XIII:

split lnL Rel.Prob

[AS|AS] -26.76 0.9548

At node XXII:

split lnL Rel.Prob

[AS|AS] -26.78 0.9342

At node III:

split lnL Rel.Prob

[EU+AS|AS] -27.28 0.5629

[AS|AS] -27.56 0.4287

At node II:

split lnL Rel.Prob

[EU|AS] -26.71 1

At node I:

split lnL Rel.Prob

[EU|EU] -26.71 1

At node XXI:

split lnL Rel.Prob

[AS|AS] -26.73 0.9789

At node XIX:

split lnL Rel.Prob

[AS|AS] -26.8 0.9099

At node IV:

split lnL Rel.Prob

[NA|AS] -26.71 1

At node XVIII:

split lnL Rel.Prob

[AS|AS] -27.02 0.7315

[EU+AS|AS] -28.56 0.1564

[EU|AS] -28.9 0.1119

At node XI:

split lnL Rel.Prob

[EU+AS|EU] -26.88 0.8432

At node V:

split lnL Rel.Prob

[EU|EU+AS] -26.79 0.9239

At node X:

split lnL Rel.Prob

[EU|EU] -26.71 0.995

At node VI:

split lnL Rel.Prob

[EU|EU] -26.71 1

At node IX:

split lnL Rel.Prob

[EU|EU] -26.74 0.9692

At node VIII:

split lnL Rel.Prob

[EU|EU] -26.9 0.8234

[EU+AF|EU] -28.46 0.174

At node VII:

split lnL Rel.Prob

[EU|AF] -26.71 1

At node XVII:

split lnL Rel.Prob

[AS|AS] -26.71 1

At node XV:

split lnL Rel.Prob

[AS|AS] -26.71 1

At node XIV:

split lnL Rel.Prob

[AS|AS] -26.71 1

At node XIII:

split lnL Rel.Prob

[AS|AS] -26.71 1

At node XII:

split lnL Rel.Prob

[AS|AS] -26.71 1

At node XVI:

split lnL Rel.Prob

[AS|AS] -26.71 1

At node XX:

split lnL Rel.Prob

[AS|AS] -26.71 1
